# *In Silico* Investigation of Amidine-Based BACE-1 Inhibitors Against Alzheimer’s Disease: SAR, Pharmacokinetics, Molecular Docking and Dynamic Simulations

**DOI:** 10.3390/ph19010005

**Published:** 2025-12-19

**Authors:** Vaibhav Gandhi, Varun Dewaker, Uma Agarwal, Vaishali M. Patil, Sung Taek Park, Hyeong Su Kim, Saroj Verma

**Affiliations:** 1 Department of Pharmacy, School of Medical and Allied Sciences, K.R. Mangalam University, Gurugram 122103, Haryana, India; vaibhav.gandhi157@gmail.com; 2 Institute of New Frontier Research Team, Hallym University, Chuncheon-si 24252, Gangwon-do, Republic of Korea; varun_dewaker1986@yahoo.com (V.D.); parkst96@naver.com (S.T.P.); 3 EIONCELL Inc., Chuncheon-si 24252, Gangwon-do, Republic of Korea; 4 Department of Pharmaceutical Chemistry, Delhi Pharmaceutical Sciences & Research University, New Delhi 110017, Delhi, India; uma5194@gmail.com; 5 Charak School of Pharmacy, Chaudhary Charan Singh University, Meerut 250004, Uttar Pradesh, India; vaishalimpatil91@gmail.com; 6 Department of Obstetrics and Gynecology, Kangnam Sacred-Heart Hospital, Hallym University Medical Center, Hallym University College of Medicine, Yeongdeungpo-gu 07441, Seoul, Republic of Korea; 7 Division of Hemato-Oncology, Department of Internal Medicine, Kangnam Sacred-Heart Hospital, Hallym University Medical Center, Hallym University College of Medicine, Yeongdeungpo-gu 07441, Seoul, Republic of Korea

**Keywords:** Alzheimer, BACE-1 inhibitors, SAR, ADME, Molecular Dynamic Simulations

## Abstract

**Background/Objective:** Alzheimer’s disease (AD) is characterized by the accumulation of amyloid-β plaques, derived from the amyloid precursor protein through sequential cleavage by β-secretase 1 (BACE-1) and γ-secretase. BACE-1 is therefore a key drug target for designing of selective inhibitors to avoid off-target effects associated with BACE-2 inhibition. The objective of this study was to design novel BACE-1 inhibitors using a structure-based drug design approach. **Methods:** A focused compound library was designed based on the SAR of N-(4-fluorophenyl)formamide derivatives. *In silico* ADME predictions were performed to assess pharmacokinetic suitability. Compounds showing favorable ADME profiles were subjected to molecular docking against the BACE-1 enzyme. The top-scoring hit, compound **9.7** (−5.48 (kcal/mol), was further evaluated using a 200 ns MD simulation to assess the stability of its binding interactions with BACE-1. **Results:** Designed compounds indicated acceptable physicochemical and ADME characteristics. Molecular docking identified compound **9.7** as exhibiting favorable binding interactions with binding pocket residues of BACE-1. The 200 ns MD simulation further confirmed the stability of the docked complex. MD simulations confirmed that 9.7 forms stable interactions with the catalytic residue ASP32 and key hydrophobic residues TRP115 and PHE108 of BACE-1. These important interactions are absent in the reference compound verubecestat. **Conclusions:** The multi-step computational analysis suggests that compound **9.7** is a promising and selective BACE-1 inhibitor. Its favorable ADME profile, favorable docking interactions, and stable MD simulation behavior highlight its potential as a hit compound for further optimization in the development of anti-Alzheimer’s agents.

## 1. Introduction

Alzheimer’s disease (AD), a progressive neurodegenerative disorder, is the leading cause of dementia globally. The main pathological marker of AD is the accumulation of oligomerized and clumped amyloid-beta (Aβ) plaques in the brain. The basic model of AD, the amyloidogenic pathway, is still controversial [1]. Various enzymes involved in pathogenesis of AD. Among those, BACE-1 (β-site APP-cleaving enzyme 1) is a crucial enzyme involved in Aβ production, making it a prime target for therapeutic intervention [2].

Early clinical setbacks with BACE inhibitors—including liver toxicity and retinal changes—highlight the complexities of targeting BACE-1 in AD. These adverse effects may be partly attributed to the dual-isoform nature of BACE, wherein unintended BACE-2 inhibition can interfere with physiological substrates. Specifically, BACE-2 inhibition disrupts two key peripheral processes: in pancreatic β-cells, it prevents TMEM27 shedding, leading to its accumulation and contributing to metabolic dysfunction and insulin resistance; in melanocytes, it impairs the processing of premelanosome protein (PMEL17), resulting in hypopigmentation [3]. Therefore, achieving higher selectivity for BACE-1 over BACE-2 is crucial for minimizing these specific mechanism-based adverse effects and improving the long-term safety profile of BACE-1 inhibitor therapy [4].

Most BACE inhibitors have shown marginal selectivity between the BACE-1 and BACE-2 isoforms, which could explain some of the observed adverse effects. Furthermore, many of these compounds belong to similar structural classes, particularly containing a cyclic amidine and a P1-P2 fluorobenzene scaffold (P1 and P2 refer to specific positions on the peptide/small-molecule inhibitor that interact with subsites (S1 and S2) in the active site of the enzyme. This structural similarity suggests that the development of more structurally diverse and highly selective BACE-1 inhibitors could provide insights into the role of BACE-1 as a drug target and mitigate off-target effects. The co-crystal (PDB; 1FKN) structure of BACE-1 and β-secretase-peptide inhibitor was introduced in year 2000 works as roadmap for exploration of inhibitors against AD [5]. Successively, number of crystal/co-crystal structure of BACE-1 were developed for exploration of 3D structure, function, and inhibition of target. Several potent BACE-1 inhibitors have been developed; however, many failed at various stages of clinical trials due to issues related to specificity, selectivity, and toxicity. CTS-21166 was among the first compounds to enter clinical trials, but it was rapidly discontinued because of inadequate CNS penetration and limited efficacy. Subsequently, numerous inhibitors—including lanabecestat, LY2811376, LY2886721, LY3202626, PF-06751979, RG7129, umibecestat, verubecestat (MK-8931), atabecestat, elenbecestat, and BI1181181 were developed but later withdrawn primarily due to safety and toxicity concerns [6].

Among the BACE-1 inhibitors developed, verubecestat exhibited potent inhibitory activity against BACE1, successfully achieving a significant ≈60% reduction in Aβ level. However, its development was terminated due to lack of cognitive efficacy and an unfavorable safety profile. This safety issue was linked to its dual inhibition of BACE1 and BACE2 [7].

In this backdrop, SAR for BACE-1 and BACE-2 selectivity has been studies and proposed a prototype SAR to develop BACE-1 selective molecules and created a library (180 compounds) of side chains for the main moiety present in verubecestat. Furthermore, ADME of designed compounds have been predicted to find out the physicochemical properties of the molecules. Based on the physicochemical profile of designed compounds, some compounds were subjected for molecular docking studies. Based on the interaction profile and binding affinity, the best docked molecule was subjected for molecular dynamic simulation study. Molecular dynamic trajectory profile showed better selectivity of designed molecule over verubecestat.

## 2. Result and Discussion

### 2.1. Rational Behind Dataset Collection of BACE-1 Inhibitors

**Figure 1:**
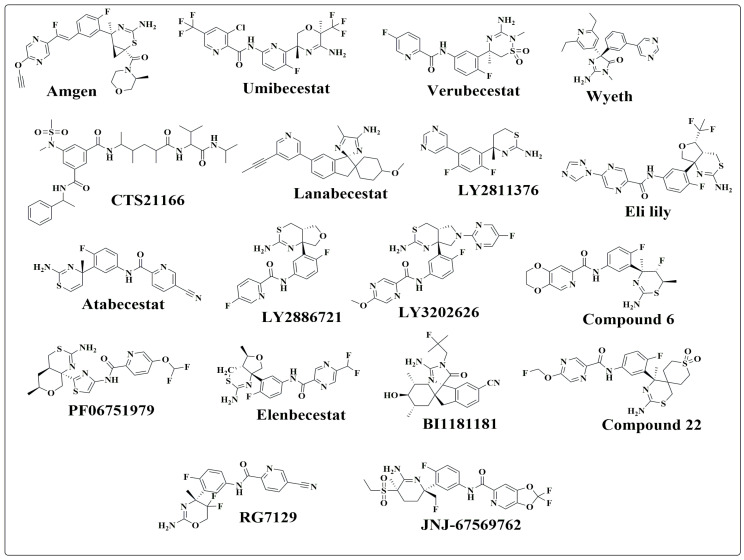
Structures of BACE-1 inhibitors.

The collection of appropriate datasets of BACE-1 inhibitors (Figure 1) is critical for understanding their therapeutic potential and addressing challenges in designing of selective and effective inhibitors. BACE-1 plays a pivotal role in the amyloidogenic pathway, catalyzing amyloid-beta (Aβ) production, a key factor in AD pathology. While targeting BACE-1 offers a promising strategy to mitigate AD progression, clinical failures due to limited efficacy, off-target effects, and adverse events highlight the need for comprehensive dataset evaluation.

Past failures have revealed significant issues, including poor CNS penetration, low oral bioavailability, rapid clearance, and adverse effects such as retinal and liver toxicity. Examples include poor blood–brain barrier (BBB) permeability with CTS-21166, retinal toxicity with LY2811376 and LY3202626, liver toxicity with RG7129 and atabecestat, and brain volume reduction with lanabecestat [8]. Off-target interactions, structural rigidity, and dysregulated neuregulin-1 processing have also been implicated in these clinical failures. Furthermore, inhibitors such as verubecestat and lanabecestat demonstrated robust Aβ-lowering effects, these molecules did not translate into cognitive improvement, highlighting the need for more selective and physiologically compatible BACE-1 inhibitors [6].

### 2.2. Analysis of the Binding Pocket of BACE-1 Through Co-Crystal (PDB) Structures and Molecular Docking Generated Docked Structures

**Figure 2:**
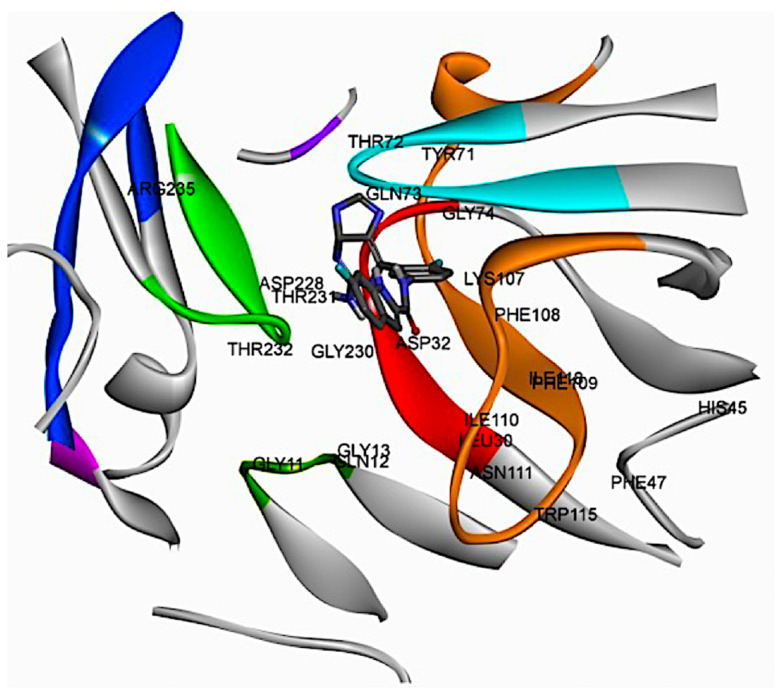
Docking Interaction diagram of verubecestat (−5.87 kcal/mol) with BACE-1 (PDB; 1FKN).

**Figure 3:**
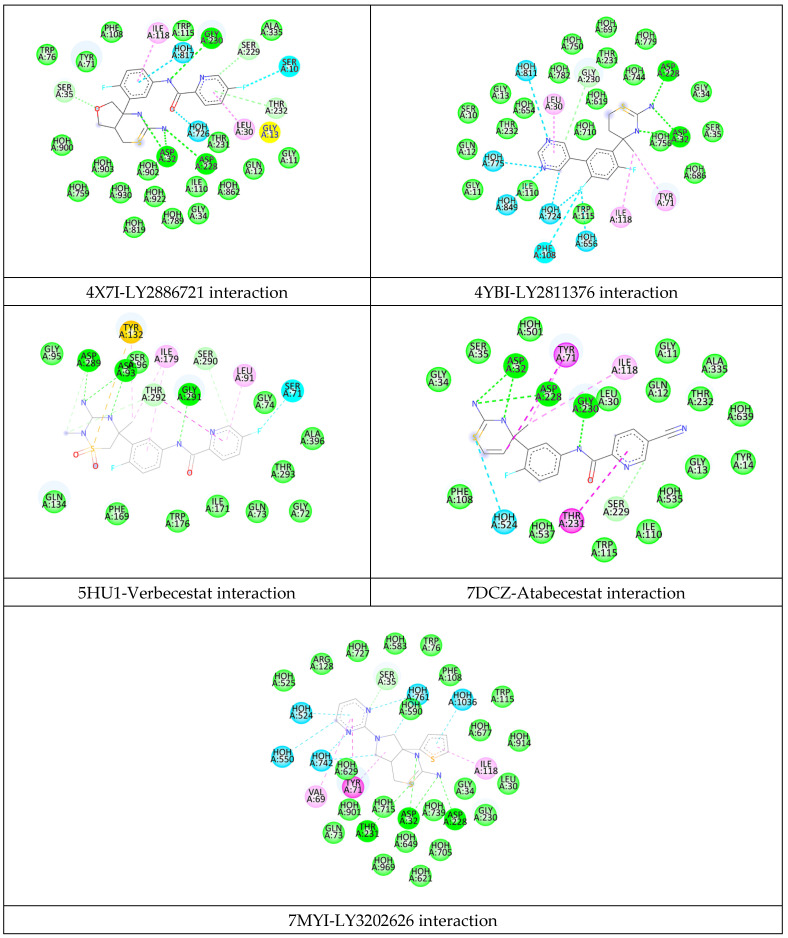
Two-dimensional interaction of crystallographic complexes (4X7I, 4YBI, 5HU1, 7DCZ, 7MYI) of BACE-1.

The co-crystal structure analysis and docking studies of several molecules, including those in clinical trials, revealed critical residues and sub-pockets within BACE-1 [9]. The residues interacting with inhibitors are distributed across various regions of the protein. The10S loop (dark green) comprises residues SER10, GLY11, GLN12, and GLY13. The red region includes LEU30, ASP32, THR33, GLY34, and SER35. The flap region (blue) features PRO70, TYR71, THR72, GLN73, GLY74, LYS75, and TRP76, while the 113S loop (brown) contains SER105, ASP106, LYS107, PHE108, PHE109, ILE110, ASN111, TRP115, ILE118, ILE125, ILE126, and ARG128. Additionally, the purple loop includes TYR198, and the green region consists of LYS224, ILE226, VAL227, ASP228, GLY230, THR231, THR232, and ASN233. The pink region contains ARG307, and the opposite loop (dark blue) includes LYS321, THR329, and VAL332 (Figure 2). Different inhibitor bound PDB structures (4X7I, 4YBI, 5HU1, 7DCZ, 7MYI) were analyzed for retrieval of important catalytic residues of BACE-1 binding pocket (Figure 3).

**Figure 4:**
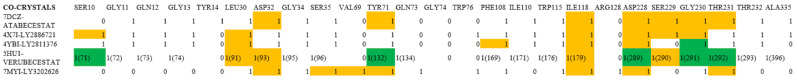
Interaction fingerprinting of BACE-1 residues with various ligands, based on complexed protein–ligand structures reported in the PDB. The residues are highlighted as green (<4 Å) and yellow (>4 Å).

**Figure 5:**
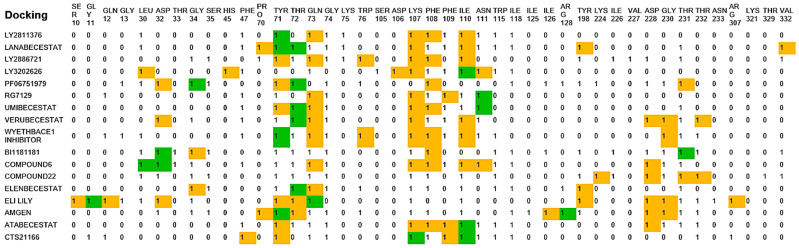
Interaction fingerprinting of BACE-1 residues with various reported ligands, based on molecular docking experiment. The residues are highlighted as green (<4 Å) and yellow (>4 Å).

In molecular docking study, the docked inhibitors (reported) of BACE-1 showed interaction residues in agreement with the co-crystal inhibitors interacting residues (Figure 4 and Figure 5, Table S3).

### 2.3. Comparison of Binding of Pocket BACE-1 and BACE-2 for Designing of Selective Inhibitors

The differences in the residues between BACE-1 (PDB: 1FKN) and BACE-2 (PDB: 2EWY) are fundamental for the design of selective BACE-1 inhibitors [5,9]. The spatial arrangement of loop regions and catalytic residues in BACE-1 differs from BACE-2, creating unique binding pockets that can be targeted for selective inhibition. By learning these structural variations, researchers can design inhibitors that specifically interact with BACE-1 binding pocket residues, improving potency, selectivity, and pharmacokinetics, while avoiding off-target effects of BACE-2. The comparison between BACE-1 and BACE-2 residues highlights crucial differences in their catalytic function, substrate binding pockets, and conformational flexibility, which are important for selective drug targeting. Both enzymes share conserved catalytic aspartates-ASP32/ASP228 in BACE-1 and ASP48/ASP241 in BACE-2 that are essential for proteolytic activity. Additionally, TYR71 (BACE-1) and TYR87 (BACE-2) are conserved within the flap region and play a significant role in substrate binding and flap dynamics. However, divergence begins in the substrate-binding subsites. For example, S1 and S2 subsites involve residues like ILE110, ILE126, and TRP115 in BACE-1, which differ structurally from their counterparts in BACE-2 (LEU126, LEU142, TRP131), altering hydrophobicity and topology, thus influencing substrate specificity and inhibitor interactions.

The presence of ASN233 in BACE-1 introduces a polar character, which may favor hydrogen bonding with inhibitors, while the corresponding LEU246 in BACE-2 maintains a more hydrophobic environment. This flexibility in BACE-1 loop could be utilized for designing selective inhibitors. Similarly, PRO70 in BACE-1 adds rigidity to the flap region, while the positively charged LYS86 in BACE-2 may alter local interactions. Collectively, these structural and chemical differences between BACE-1 and BACE-2 residues provide valuable insights for designing selective inhibitors, particularly important for targeting BACE-1 in AD while minimizing off-target effects on BACE-2 (Table 1) [5,10,11]. Molecular docking analyses of compound **9.7** with BACE-1 (PDB ID: 1FKN) and BACE-2 (PDB ID: 7N4N) indicated reasonable differences in the binding-pocket interactions of the two enzymes, as detailed in Table 1 (Table S4).

### 2.4. SAR and Rationale Design of BACE-1 Selective Inhibitor

**Figure 6:**
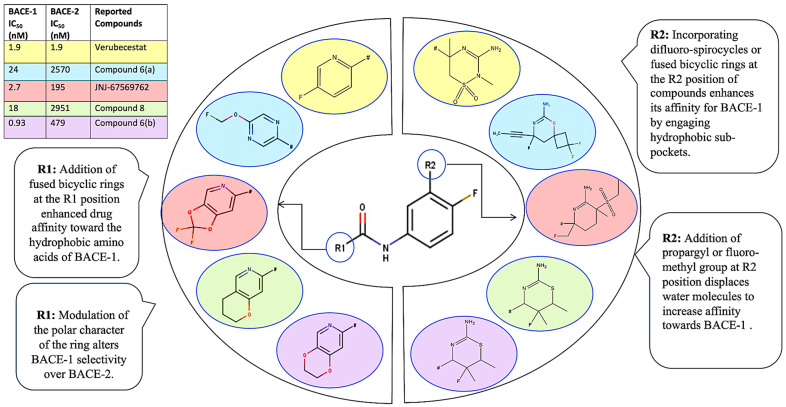
Proposed SAR graphical representation for design of new compounds. Biological activity of reported verubecestat [15], compound **6**(**a**) [15], JNJ67569762 [12], compounds **8** and **6**(**b**) [16] considered for understanding of SAR.

**Figure 7:**
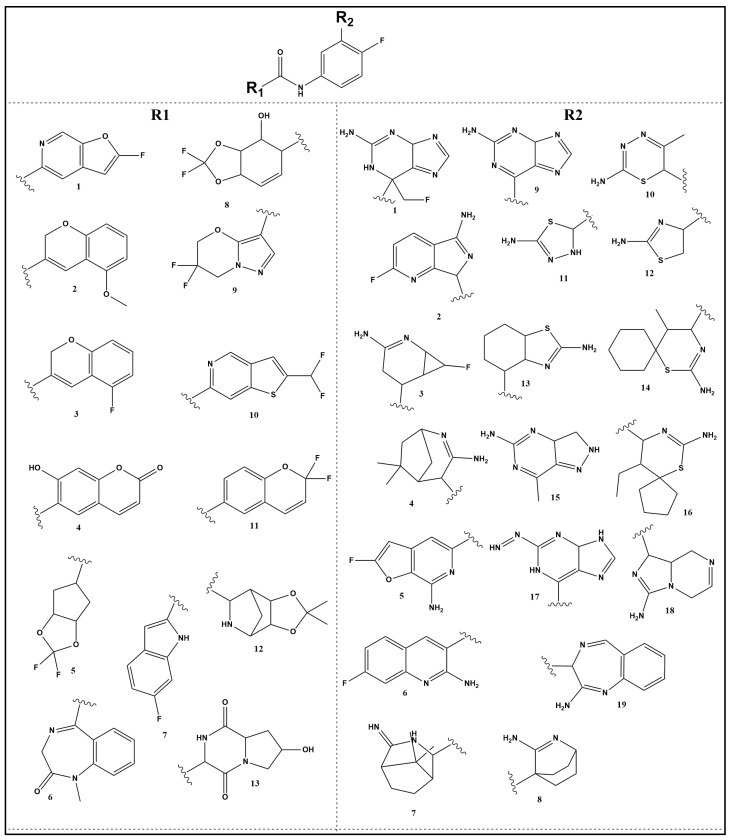
Library of designed compounds generated by systematically varying R1 and R2 substituents. Each compound was named using the (R2.R1) format, where the R1 and R2 were combined (e.g., a compound with R1 = 1 and R2 = 1 was named 1.1, while R1 = 7 and R2 = 9 was named 9.7). A total of 180 compounds were designed through these combinations: 1.1, 1.2, 1.3, 1.4, 1.5, 1.6, 1.7, 1.8, 1.9, 1.10, 1.11, 1.12, 2.1, 2.2, 2.3, 2.4, 2.5, 2.6, 2.7, 2.8, 2.9, 2.10, 2.11, 2.12, 3.1, 3.2, 3.3, 3.4, 3.5, 3.6, 3.7, 3.8, 3.9, 3.10, 3.11, 3.12, 4.1, 4.2, 4.3, 4.4, 4.5, 4.6, 4.7, 4.8, 4.9, 4.10, 4.11, 4.12, 5.1, 5.2, 5.3, 5.4, 5.5, 5.6, 5.7, 5.8, 5.9, 5.10, 5.11, 5.12, 6.1, 6.2, 6.3, 6.4, 6.5, 6.6, 6.7, 6.8, 6.9, 6.10, 6.11, 6.12, 7.1, 7.2, 7.3, 7.4, 7.5, 7.6, 7.7, 7.8, 7.9, 7.10, 7.11, 7.12, 8.1, 8.2, 8.3, 8.4, 8.5, 8.6, 8.7, 8.8, 8.9, 8.10, 8.11, 8.12, 9.1, 9.2, 9.3, 9.4, 9.5, 9.6, 9.7, 9.8, 9.9, 9.10, 9.11, 9.12, 10.1, 10.2, 10.3, 10.4, 10.5, 10.6, 10.7, 10.8, 10.9, 10.10, 10.11, 10.12, 11.1, 11.2, 11.3, 11.4, 11.5, 11.6, 11.7, 11.8, 11.9, 11.10, 11.11, 11.12, 12.1, 12.2, 12.3, 12.4, 12.5, 12.6, 12.7, 12.8, 12.9, 12.10, 12.11, 12.12, 13.1, 13.2, 13.3, 13.4, 13.5, 13.6, 13.7, 13.8, 13.9, 13.10, 13.11, 13.12, 14.1, 14.2, 14.3, 14.4, 14.5, 14.6, 14.7, 14.8, 14.9, 14.10, 14.11, 14.12, 15.1, 15.2, 15.3, 15.4, 15.5, 15.6, 15.7, 15.8, 15.9, 15.10, 15.11, and 15.12.

The development of BACE-1 inhibitors is constrained by the necessity for high selectivity. Although verubecestat successfully reduced brain amyloid load and altered CSF biomarkers, it lacked cognitive efficacy in dementia patients. This failure is hypothesized to be due to BACE-1 role in normal synaptic function and the potential for adverse outcomes from excessive Aβ suppression [6]. Furthermore, off-target BACE-2 inhibition by verubecestat likely contributed to adverse effects. Consequently, these findings mandated the development of highly selective BACE-1 inhibitors, directly leading to the design of JNJ-65212173, which was derived from the verubecestat scaffold [12]. A key strategy for improving BACE-1 selectivity over BACE-2 involved targeting explicit water molecules in the S2′ pocket of the enzyme. Fujimoto et al. incorporated a 1,4-oxazine ring in place of unsaturated 1,3-thiazine moiety and further added a propynyl group which successfully displaced one of these water molecules in BACE-1, enhancing selectivity. The introduction of fluoromethyl group in place of propynyl in new molecules retained this selective displacement. Additionally, modifying the head group conformation, such as incorporating spirocycles or difluorocyclobutyl groups, improved potency and selectivity. These modifications led to reduction in P-gp efflux and hERG inhibition. The head group variations provided insights into stabilizing the bioactive conformation of the inhibitor [13,14]. Fujimoto et al. explored tail variations, such as replacing methoxy groups with 1,4-dioxane in heterocyclic rings further contributed to improving BACE-1 selectivity but increased chances of drug-induced liver injury. The 2,2-difluoro-1,3-dioxolane derivative demonstrated high potency, selectivity, and reduced reactive metabolites. Deuterating the ethylene bridge or fluorinating the bridge further improved metabolic stability. Despite the promising pharmacological profile of JNJ-65212173, single-dose escalation studies revealed delayed-onset CNS toxicity in dogs, suggesting that the core structure may contribute to the adverse effects [15]. The selectivity of BACE-1 inhibitors is also influenced by the 10S loop conformation. Ueno et al. explored bulky tail groups like trifluoroethoxy moiety or bicyclic tails like annulated pyridines to achieve higher selectivity towards BACE-1 to access 10S loop. In BACE-1, 10S loop is in an “open” conformation, allowing for favorable interactions with inhibitors. However, in BACE-2, the 10S loop is in a “closed” conformation preventing the inhibitor reach S3 subsite. Structural analysis has shown that compounds targeting the 10S loop in BACE-1 can improve selectivity by destabilizing BACE-2 [16]. Prati et al. explained the importance of aminohydantoin and aminoquinoline moieties as tail-group features contributing to BACE inhibitory activity [17]. Considering the important roles of head groups (R2, e.g., amidine-containing spirocycles or heterocycles) and tail groups (R1, e.g., annulated pyridines, dioxolanes, or other fluoro containing bulky heterocycles) in determining binding pocket selectivity, we propose a SAR for designing BACE-1 selective inhibitors through variations in both R 2 and R 1 functional groups (Figure 6). Based on knowledge, we designed list of new inhibitors for further study (Figure 7).

### 2.5. In Silico Study of Designed Molecules

An *in silico* study was performed using the SwissADME web tool to identify drug candidates. Comprehensive filtering was involved against established criteria, including the rules defined by Lipinski, Veber, Muegge, Ghose, and Egan. Additionally, to favor compounds with the potential to penetrate the central nervous system, a specific filter based on lipophilicity—a property known to influence BBB permeation was applied. Specifically, only compounds exhibiting a consensus logP value within the range of 2.0–3.5 were retained, as this range is often associated with the balanced solubility required for CNS activity. Additionally, compounds were filtered by considering Pgp substrate status, CYP inhibition profile, bioavailability score, and synthetic accessibility (Table 2). Following this approach 7 compounds were selected for the subsequent molecular docking study.

### 2.6. Molecular Docking

In molecular docking, compounds **3.4**, **3.11**, **6.8**, **9.11**, **9.7**, **10.1**, and **11.11** were found to exhibit good binding scores and favorable binding-pocket interactions.

For molecule 3.4, hydrogen bonds were formed with LYS107 through its amidine group and with THR72 via the amide and hydroxyl groups of the chromen-2-one moiety. The fluoro-phenyl group was stabilized through interaction with GLY230. Hydrophobic π–π interactions with TYR198 and π-alkyl interactions with ILE126 were observed for the chromen-2-one ring. A π-anion interaction between ASP32 and the fluoro-phenyl moiety was also detected. For molecule 3.11, hydrogen bonds were formed with LYS107 through the amidine group and with THR72 through the amide group. Hydrophobic π-alkyl interactions were observed between LEU30 and the 7-fluoro-2-azabicyclo[4.1.0]hept-2-en-3-amine moiety. The 2,2-difluoro-2H-chromene moiety was engaged in π–π T-shaped interactions with TYR198 and π-alkyl interactions with ILE126. The catalytic residue ASP32 formed a π-anion interaction with the fluoro-phenyl ring. Halogen bonding with GLY230 was also observed. For molecule 6.8, hydrogen bonding was observed through THR72 and GLY34 with the amide linkage. The 2,2-difluoro-3a,4,5,7a-tetrahydrobenzo[d][1,3]dioxol-4-ol moiety was bound to ARG128 and PRO70. Hydrophobic π-alkyl interaction was formed between ILE110 and the 7-fluoroquinolin-2-amine moiety. Halogen bonding was observed between GLY230 and the fluoro-phenyl group, and an interaction of the 7-fluoroquinolin-2-amine moiety with LYS107 was also noted. For molecule 9.7, hydrogen bonds were formed with THR231 and ASP228 through the amidine group of the purine moiety. π-Alkyl interactions with LYS107 were observed for the 6-fluoro-1H-indole moiety, while TYR71 exhibited π–π stacking with the fluoro-phenyl ring. GLN73 formed hydrogen bonds with both the indole moiety and the amide linkage. The inhibitor was found to anchor ASP228 along with flap residues. For molecule 9.11, hydrogen bonds were formed with GLN73, LYS107, and PHE108 through the amidine-containing purine moiety. THR72 and GLY34 formed hydrogen bonds with the amide linkage. Additional interactions included hydrogen bonding with ARG128, π-alkyl interactions with ILE126, and π–π T-shaped interactions with TYR198 involving the 2,2-difluoro-2H-chromene ring. GLY230 showed interaction with fluoro-phenyl moiety.

**Figure 8:**
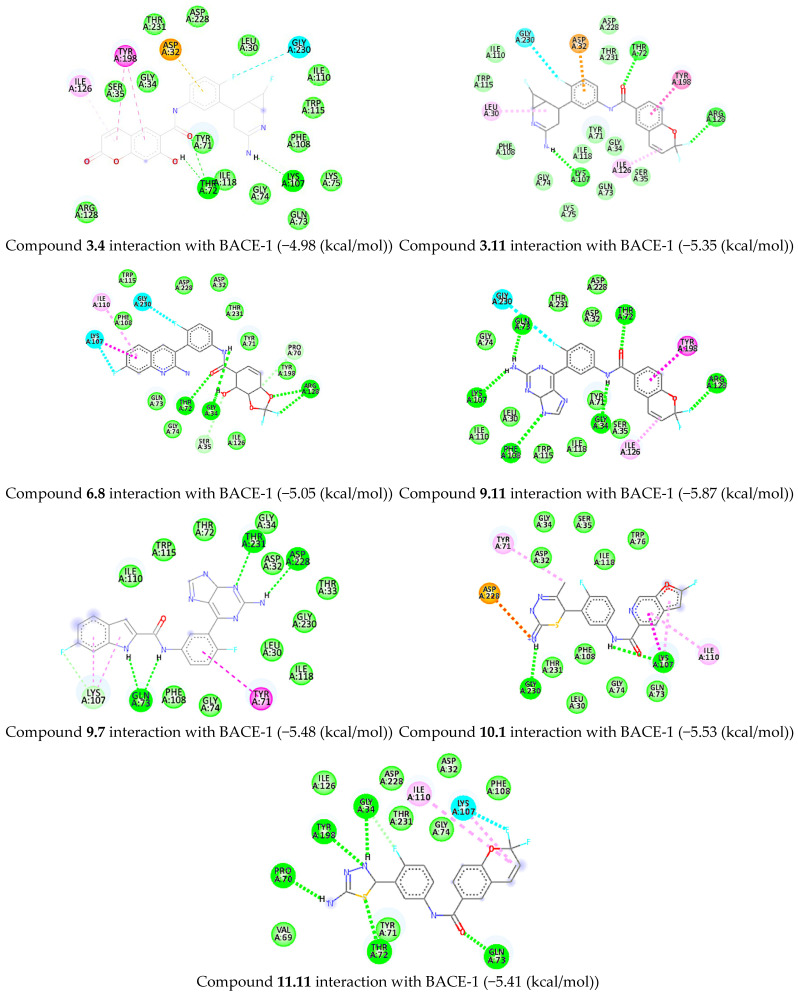
Binding pocket interactions of docked complexes of selected molecules.

For molecule 10.1, hydrogen bonding with GLY230 and π-alkyl interactions with TYR71 were observed through the 5-methyl-6H-1,3,4-thiadiazin-2-amine moiety. Hydrophobic π-alkyl interactions between LYS107 and ILE110 with the 2-fluorofuro[2,3-c]pyridine moiety were detected. An ionic dipole interaction was formed with ASP228 by the thiadiazine ring, and hydrogen bonding between LYS107 and the amide group was noted. This compound predominantly anchored the 113s-loop residues. For molecule 11.11, hydrogen bonds were formed with PRO70, TYR198, GLY34, and THR72 through the 4,5-dihydro-1,3,4-thiadiazol-2-amine moiety, while GLN73 formed a hydrogen bond with the amide linkage. Halogen bonding occurred between LYS107 and the 2,2-difluoro-2H-chromene moiety, and between GLY34 and the thiadiazole moiety. Alkyl interactions with ILE110 and LYS107 further stabilized the chromene ring, enabling the anchoring of flap and 113s-loop residues. Collectively, these interactions highlighted the crucial roles of hydrogen bonding, hydrophobic interactions, and halogen bonding in stabilizing the inhibitors within the active site (Figure 8).

Among the docked compounds, compound **9.7** was selected for molecular dynamics simulation studies due to its better ADME profile and favorable interactions with key catalytic-site residues (Asp32, Tyr71, Gln73, Lys107, and Asp228) of BACE-1.

### 2.7. Molecular Dynamic Studies

**Figure 9:**
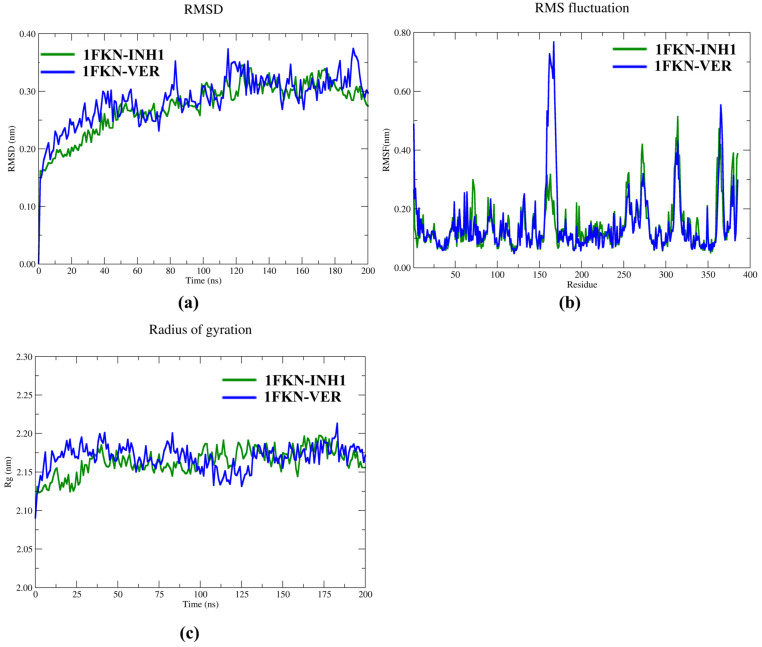
Graphical representation of the 200 ns MD simulation data: (**a**) RMSD of Cα atoms, (**b**) RMSF profile of protein residues, and (**c**) radius of gyration (Rg) plot.

The structural stability of the 1FKN-INH1 (9.7) and 1FKN-VER (verubecestat) protein–ligand complexes (Figure 9a) was assessed over a 200 ns MD simulation by analyzing the root mean square deviation (RMSD) of both the protein backbone and the corresponding bound ligands (INH1 and VER). Prior to RMSD analysis, all protein trajectories were aligned to their initial backbone conformation using least-squares fitting to eliminate global translational and rotational motions. The average protein RMSD for the 1FKN-INH1 system was 0.273 ± 0.048 nm, while the 1FKN-VER protein exhibited a slightly higher average deviation of 0.286 ± 0.045 nm. These values indicate that both protein systems maintained consistent structural integrity throughout the simulation, with moderate flexibility and no signs of unfolding or instability. The corresponding ligand RMSD profiles also reflected strong conformational stability within the binding pockets. The 1FKN-bound inhibitor (1FKN-INH1) maintained an average RMSD of 0.118 ± 0.029 nm, whereas VER in the 1FKN-VER complex remained even more stable with an average RMSD of 0.103 ± 0.022 nm. These low RMSD values suggest tight ligand binding and minimal positional drift from the initial docked conformations. Collectively, the results indicate that both proteins and ligands undergo rapid convergence and achieve the structural stability.

The global compactness and conformational dynamics of the 1FKN-INH1 and 1FKN-VER protein–ligand complexes were assessed by monitoring the radius of gyration (Rg) of both protein backbones and their respective ligands over a 200 ns MD simulation (Figure 9c). For the protein components, the average Rg of 1FKN-INH1 was 2.164 ± 0.017 nm, while 1FKN-VER exhibited a similar profile with an average Rg of 2.169 ± 0.017 nm. The low standard deviation in Rg across the simulation indicates consistent tertiary structural integrity, with no significant unfolding or collapse events. These stable Rg profiles, corroborated by RMSD trends, reflect the preservation of global folding and support the reliability of these trajectories for downstream energetic and interaction analysis. For the ligands, the radius of gyration further confirmed their stable and compact behavior within the binding pockets. The 1FKN-bound inhibitor (INH1) maintained an average Rg of 0.510 ± 0.007 nm, while VER, bound to 1FKN, displayed slightly greater compactness with an average Rg of 0.479 ± 0.005 nm. These low Rg values reflect well-packed and rigid ligand conformations, with no evidence of conformational expansion or strain within the binding sites.

Collectively, the Rg analyses confirm that both protein and ligand components maintained structurally compact and dynamically stable profiles throughout the 200 ns simulations.

#### 2.7.1. Global Flexibility Trends

**Figure 10:**
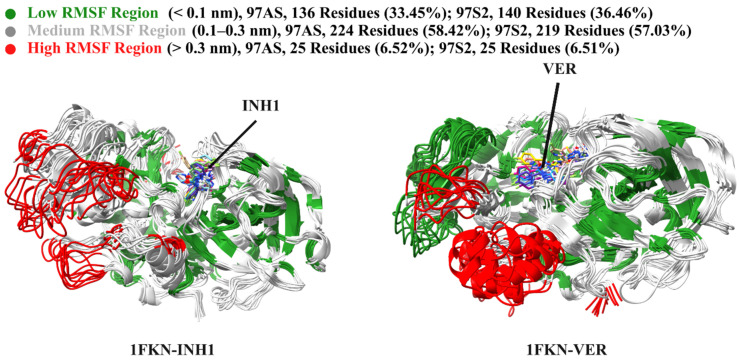
Superposition of 11 representative frames extracted every 20 ns from the 200 ns MD simulation, illustrating the RMSF distribution of protein residues in the presence of their respective inhibitors, INH1 (9.7) and VER. Throughout the simulation, both ligands remained stably bound within the binding pocket. Residue-wise fluctuations are color-coded: green indicates low flexibility (RMSF < 0.1 nm), gray represents medium flexibility (0.1–0.3 nm), and red highlights high flexibility (RMSF > 0.3 nm).

The root mean square fluctuation (RMSF) analysis was conducted to evaluate residue-level flexibility in the 1FKN-INH1 and 1FKN-VER protein–ligand complexes, respectively (Figure 9b). Both systems displayed broadly similar flexibility profiles, with most residues falling into low to medium flexibility categories, indicative of overall structural stability (Figure 10). In 1FKN-INH1, 136 residues (35.45%) exhibited low flexibility (<0.1 nm), 224 residues (58.42%) showed medium flexibility (0.1–0.3 nm), and 25 residues (6.52%) were highly flexible (>0.3 nm). Similarly, 1FKN-VER had 140 low-flexibility residues (36.46%), 219 with medium flexibility (57.03%), and 25 highly flexible residues (6.51%) (Figure 10). These results suggest that both inhibitors maintain comparable residue dynamics, with VER inducing a slightly higher degree of stabilization in structured regions. Low-flexibility residues likely reside in secondary structure elements such as α-helices and β-sheets, exhibiting minimal movement. Medium-flexibility residues, which form the majority in both systems, are typically located in surface loops or connecting regions and reflect moderate backbone mobility that supports structural adaptability. In contrast, highly flexible residues (≈6% of residues, ~25 residues in each protein) are generally confined to terminal regions or solvent-exposed loops and contribute to localized conformational fluctuations.

**Figure 11:**
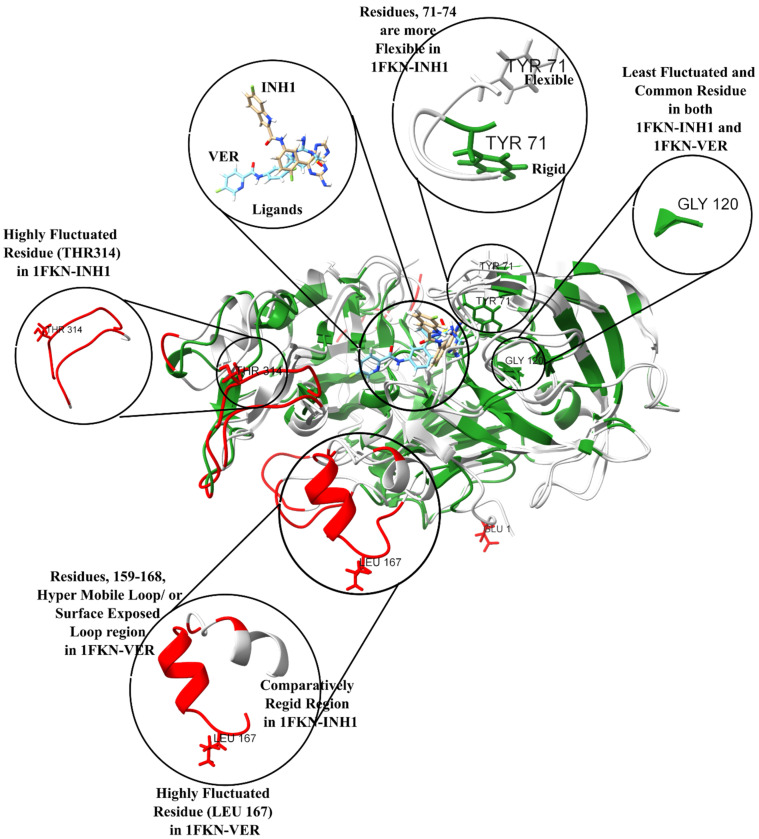
Key RMSFs highlighting the minimum and maximum fluctuating residues, as well as highly mobile regions mapped onto the protein structure in the presence of different ligands (INH1 and VER). The orientation of the inhibitors within the binding pocket is also shown, as observed at the end of the 200 ns MD simulation. The color codes are same as in Figure 10.

The most fluctuating residue in 1FKN-INH1 was residue 314, with an RMSF of 0.5143 nm, whereas the least fluctuating was residue 120, at 0.0497 nm (Figure 11). In 1FKN-VER, the maximum fluctuation was observed at residue 167, reaching 0.7678 nm, while the least fluctuating residue remained residue 120, with a slightly lower RMSF of 0.0488 nm, suggesting a conserved core region (Figure 11). The RMSF analysis revealed distinct flexibility patterns across the N-terminal, mid-region, and C-terminal domains of the protein, highlighting the influence of ligand binding on structural dynamics. In the N-terminal region (residues 1–20), the 1FKN-VER complex exhibited noticeably higher fluctuations compared to 1FKN-INH1, with several residues shifting from medium to high flexibility. For instance, residue 1 showed an RMSF increase from 0.2642 nm in 1FKN-INH1 to 0.4877 nm in 1FKN-VER, indicating ligand-induced destabilization in solvent-exposed or loop regions. The mid-region (residues 21–365), which constitutes the structural core, was largely conserved between the two systems, though residues 162 to 167 showed substantial increases in flexibility in 1FKN-VER —most notably residue 167, where the RMSF rose by 0.5402 nm. This suggests a local effect of the ligand on dynamic hotspots likely associated with functional loop regions. Overall, the distribution of flexibility classes showed a slight increase in low-flexibility residues in 1FKN-VER and a minor decrease in medium-flexibility residues, while the count of highly flexible residues remained stable. In contrast, the C-terminal region (residues 366–385) exhibited a dual response: some residues, such as 366 and 367, became more flexible in 1FKN-VER, shifting to high RMSF values, while others like residues 378 to 385 demonstrated reduced flexibility, transitioning from high to medium. These changes suggest localized allosteric stabilization or destabilization induced by ligand binding. Notably, residue 120 remained the most rigid in both systems, reflecting a conserved structural role (Figure 11). Collectively, these findings demonstrate that the ligand in 1FKN-VER modulates the protein’s dynamic landscape, enhancing flexibility in specific loop and terminal regions while preserving core structural stability, which may have implications for ligand-induced functional regulation or allosteric signaling. The comparison revealed that 1FKN-VER exhibited a higher proportion of highly fluctuating residues, likely reflecting enhanced loop dynamics or domain mobility induced by ligand binding (Figure 11). These flexible regions may be associated with functionally relevant motions or binding-induced conformational changes and warrant further structural investigation.

While the overall distribution is alike, certain regions differ markedly in flexibility between 1FKN-INH1 and 1FKN-VER. The RMSF comparison between 1FKN-INH1 and 1FKN-VER revealed several structurally important segments with pronounced differences in flexibility, highlighting ligand-induced modulation of protein dynamics. In the N-terminal region, residues 1–5 exhibited markedly increased flexibility in 1FKN-VER, with RMSF values ranging from 0.183 to 0.488 nm, compared to 0.069 to 0.264 nm in 1FKN-INH1. This suggests enhanced mobility of the N-terminal loop in the presence of the ligand bound to 1FKN-VER. A particularly striking observation was made in the segment spanning residues 159–168 (Figure 11), which demonstrated a consistent and substantial increase in flexibility in 1FKN-VER (RMSF ~0.48–0.77 nm in 1FKN-VER vs. only ~0.18–0.32 nm in 1FKN-INH1). This differential flexibility strongly indicates the presence of a hyper-mobile loop or surface-exposed segment in 1FKN-VER that remains comparatively rigid in 1FKN-INH1 (Figure 11). These observations were identified using a sliding window analysis (window size = 5 residues), in which the maximum RMSF values for each segment were compared. Only segments with an absolute difference of ≥ 0.2 nm between 1FKN-VER and 1FKN-INH1 were retained, ensuring that only biologically meaningful deviations were considered. Such ligand-specific enhancements in local dynamics may be functionally relevant, potentially contributing to altered binding interactions, loop rearrangements, or allosteric signaling pathways triggered by ligand engagement. These dynamic shifts warrant further structural and energetic analysis to understand their role in functional divergence between the two complexes. Conversely, a loop around residues 71–74 is notably more flexible in 1FKN-INH1 than in 1FKN-VER (Figure 11). In 1FKN-INH1, this loop fluctuates in the medium-to-high range (RMSF 0.16–0.30 nm, with Tyr71 reaching ~0.30 nm), whereas the same residues in 1FKN-VER are much more rigid (0.095–0.17 nm) (Figure 11). Additionally, residues, 68–74, 195–200, 269–272, and 307–310, also showed ligand-sensitive flexibility. These data highlight that ligand-specific effects on protein dynamics are concentrated in particular loop regions, where one ligand induces enhanced flexibility while the other promotes local stabilization—despite the protein sequence being identical. Aside from such regions, the two systems maintain a similar backbone stability elsewhere (most structured regions have RMSF <0.1 nm in both). Overall, 1FKN-INH1 than in 1FKN-VER share a stable core and moderately flexible surface, punctuated by a few distinct highly mobile loops unique to each (Tables S6 and S7).

#### 2.7.2. Active Site Residue Dynamics

The initial superposition of the docked structures of 1FKN-INH1 and 1FKN-VER yielded an RMSD of 0.00 Å across both 385 pruned atom pairs and the complete set of 385 atom pairs, indicating perfect alignment. After 200 ns of molecular dynamics simulation, the RMSD between the two trajectories increased to 1.049 Å (pruned) and 2.361 Å (all atom pairs), reflecting conformational divergence over time. For 1FKN-INH1, comparison between the initial docked pose and the final structure at 200 ns showed an RMSD of 1.112 Å over 298 pruned atoms, and 2.228 Å across all 385 atom pairs. Similarly, for 1FKN-VER, the RMSD values were 1.118 Å over 284 pruned atoms and 2.693 Å across all 385 atom pairs. These results suggest that both complexes underwent moderate structural rearrangements during the simulation, with slightly higher divergence observed in 1FKN-VER.

**Figure 12:**
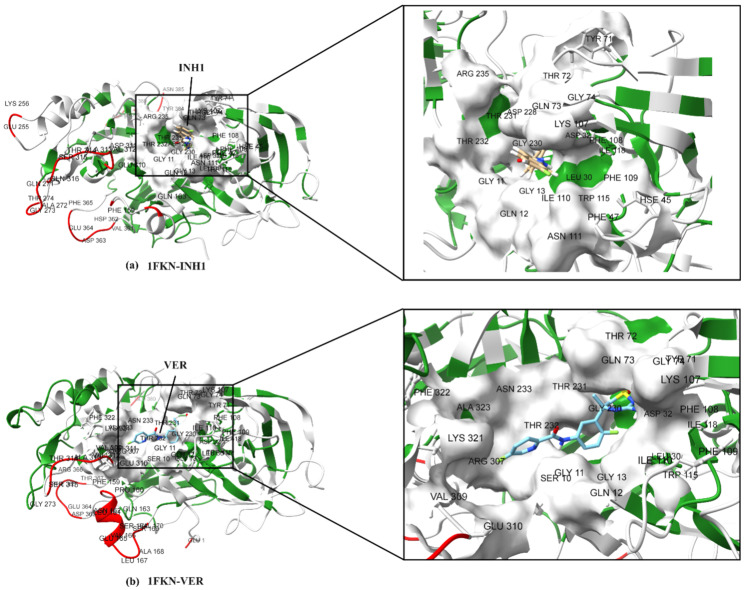
RMSF distribution mapped onto the final frame of the 200 ns MD simulation. The color scheme follows that of Figure 10: green indicates low fluctuations (RMSF < 0.1 nm), gray represents medium fluctuations (0.1–0.3 nm), and red highlights high fluctuations (RMSF > 0.3 nm). (**a**,**b**) depict the RMSF distribution alongside the active site residues (identified using ProLIF), where the ligands remained stably bound throughout the simulation. These residues predominantly exhibit low to medium flexibility. To enhance clarity, residue labels are shown only for regions of high flexibility and for residues within the active site. A close-up inset is provided to illustrate the positioning of the ligands within the active site pocket.

**Figure 13:**
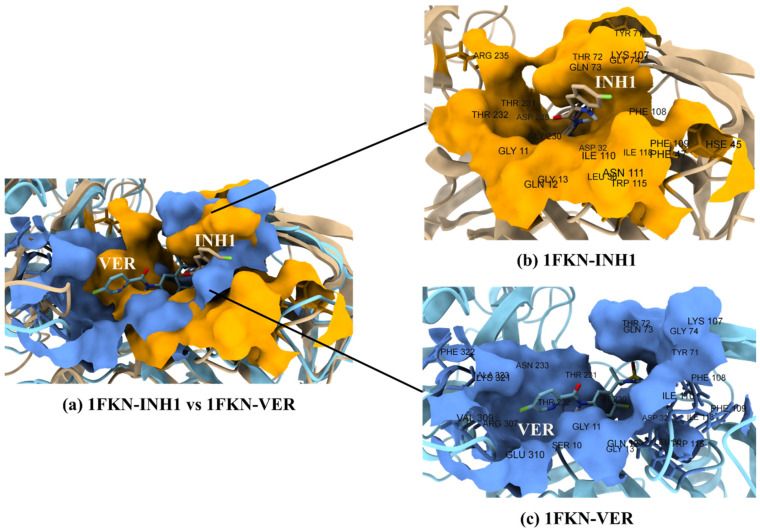
(**a**) Superimposition and surface representation of the protein–ligand complexes 1FKN–INH1 (yellow) and 1FKN–VER (blue), highlighting the active site pocket in surface mode (snapshot from the final frame of the 200 ns MD simulation). This comparison illustrates conformational differences and residue behavior within the binding site. (**b**) Close-up view of the binding pocket residues interacting with the ligand INH1. (**c**) Close-up view of the pocket residues interacting with the inhibitor VER.

The active site residues show flexibility patterns that correlate with their structural roles (loop vs. core) and differ between 1FKN-INH1 and 1FKN-VER in insightful ways (Figure 12 and Figure 13). Both proteins share a common set of ~18 active-site residues, i.e., GLY11, GLN12, GLY13, LEU30, ASP32, TYR71, THR72, GLN73, GLY74, LYS107, PHE108, PHE109, ILE110, TRP115, ILE118, GLY230, THR231, and THR232, with several additional unique active-site residues in both (1FKN-INH1, HIS45, PHE47, ASN111, ASP228, ARG235; 1FKN-VER, SER10, ASN233, ARG307, VAL309, GLU310, LYS321, PHE322, and ALA323). We examined each active-site residue’s RMSF, its fluctuation category (low/medium/high), and structural localization.

N-terminal active-site loop (residues SER10–GLY13): This segment contributes to the active site in both proteins (except SER10, which is active-site in 1FKN-VER only). These loop residues display low-to-moderate flexibility in both 1FKN-INH1 and 1FKN-VER (Figure 12 and Figure 13). For instance, GLY11 and GLN12 (loop) fluctuate by ~0.12–0.18 nm (medium) in 1FKN-INH1 and ~0.12–0.15 nm in 1FKN-VER. GLY13, at the junction of a β-sheet and a loop, is even more rigid (RMSF 0.107 nm in 1FKN-INH1, 0.091 nm in 1FKN-VER—low category). The similarity in this region suggests a comparably stable N-terminal loop in both systems. SER10 (active in 1FKN-VER, loop) shows a modest 0.145 nm fluctuation in 1FKN-VER (low-medium), virtually the same as in 1FKN-INH1 (0.165 nm, where it is not part of the active site), indicating that inclusion in the active site did not significantly alter its mobility.

Residues LEU30 and ASP32 play critical roles in maintaining the structural integrity of the active site in both 1FKN-INH1 and 1FKN-VER. Both residues are located within a β-sheet region, contributing to the rigid core of the protein. In 1FKN-INH1, LEU30 exhibits a very low RMSF value of 0.0786 nm, while Asp32 shows an even slightly lower RMSF of 0.0692 nm, indicating minimal flexibility. Similarly, in 1FKN-VER, LEU30 and ASP32 maintain comparable rigidity, with RMSF values of 0.0757 nm and 0.0734 nm, respectively. This consistent low flexibility across both protein variants highlights their role as structural anchors, ensuring that the catalytic geometry remains stable and properly aligned (Figure 12 and Figure 13). The preservation of rigidity at these positions suggests that, despite other dynamic differences between 1FKN-INH1 and 1FKN-VER, the fundamental framework supporting enzymatic activity is conserved. Thus, LEU30 and ASP32 likely contribute to the overall catalytic efficiency and structural robustness of the active site architecture in both protein forms.

Mid-protein active-site loop (residues HIS45 and PHE47 in 1FKN-INH1): 1FKN-INH1 uniquely includes HIS45 (HSE45) and PHE47 as active-site residues, located in a loop. They exhibit moderate flexibility (RMSF ~0.13–0.17 nm). In 1FKN-VER, these residues are not considered part of the active site and have nearly identical RMSF values (~0.12–0.16 nm) (Figure 12 and Figure 13). Despite this functional difference, their flexibility stays about the same in both proteins. This suggests that even though they are removed from the active site in 1FKN-VER, they do not become more or less mobile. The loop stays moderately flexible and might help shape the active-site pocket in 1FKN-INH1 without playing a major dynamic role.

Active-site loop around TYR71–GLY74: This loop is part of the active site in both proteins and shows one of the clearest differences in flexibility (Figure 12 and Figure 13). In 1FKN-INH1, TYR71, THR72, GLN73, and GLY74 (all in a surface-exposed loop) are quite flexible, with RMSF values in the upper-medium range (0.16–0.30 nm). TYR71 in 1FKN-INH1 reaches ~0.300 nm (borderline high flexibility), indicating this loop can move substantially. In contrast, in 1FKN-VER the same residues are much more constrained (RMSF ~0.095–0.174 nm), all falling in the low or low-medium category. This dramatic reduction (e.g., TYR71 from 0.300 nm in 1FKN-INH1 to 0.095 nm in 1FKN-VER) points to a stabilization of the active-site loop in 1FKN-VER. Structurally, this loop likely acts as a lid or flexible binding region over the active site. The 1FKN-INH1 variant’s “lid” is highly flexible (potentially allowing greater conformational sampling for substrate binding or product release), whereas 1FKN-VER’s lid is comparatively rigid, which might maintain a more closed and stable active-site conformation during the simulation.

Active-site segment LYS107–ASN111: This region (a loop in both proteins) contains LYS107, PHE108, PHE109, ILE110 (active in both), and ASN111 (active in 1FKN-INH1 only). All these residues show low-to-moderate fluctuations, indicating a moderately flexible loop that is similar in both systems (Figure 12 and Figure 13). LYS107 and ILE110 have RMSF around 0.13–0.19 nm (medium flexibility), whereas the two phenylalanine (PHE108, PHE109) are more rigid (≈0.09–0.10 nm, low), likely due to packing or aromatic stacking that dampens their motion. Notably, ASN111 (loop) has identical moderate flexibility in both 1FKN-INH1 and 1FKN-VER (~0.149 nm), even though it is only annotated as an active-site residue in 1FKN-INH1. The consistency of the 107–111 loop’s dynamics between the proteins suggests that this loop remains a semi-flexible part of the active-site region in either context. It may allow slight adjustments during ligand binding, but its baseline mobility does not drastically change between 1FKN-INH1 and 1FKN-VER.

TRP115 and ILE118: These residues form part of the active site in both proteins, but they reside in very different structural environments (TRP115 in a loop, ILE118 in a β-sheet). Interestingly, both are very rigid. TRP115, despite being in a loop, has one of the lowest RMSF values among active-site loops (0.078 nm in 1FKN-INH1, 0.088 nm in 1FKN-VER—firmly low). This implies TRP115 is likely anchored by stable interactions (pi-stacking and hydrophobic interaction in case of INH1, while VER only showed hydrophobic interaction) that restrict its movement (Figure 12 and Figure 13). ILE118 is located in a beta-sheet region of the active site and accordingly shows low flexibility as well (0.080 nm in 1FKN-INH1, 0.065 nm in 1FKN-VER). As expected for a β-sheet residue, ILE118 acts as a rigid scaffold point within the active-site pocket. The concordant rigidity of these residues in both proteins underscores that certain active-site positions serve as structural anchors (maintaining the active-site architecture), and their dynamics are inherently limited by their environment.

Active-site region ASP228-ARG235: This segment includes both loop and sheet elements and has mixed active-site involvement: ASP228 and ARG235 are active-site residues in 1FKN-INH1 only, GLY230–THR232 in both, and ASN233 in 1FKN-VER only (Figure 12 and Figure 13). All of these residues fall in the low-to-moderate RMSF range, indicating a relatively stable region. GLY230, THR231, and THR232 (loop) fluctuate by only ~0.11–0.15 nm in both proteins, which is surprisingly low for a loop—suggesting this loop might be stabilized by the surrounding structure (perhaps forming part of a binding pocket). The β-sheet residue ARG235 (active in 1FKN-INH1) has a modest RMSF (~0.14 nm), similar to its value in 1FKN-VER (~0.13 nm despite not being active-site there). Likewise, ASN233’s flexibility is ~0.11–0.13 nm in both contexts. These observations show that the 228–235 region is fairly rigid in both proteins, and even where active-site designation shifts (e.g., ASN233 or ARG235 swapping roles), the backbone mobility remains unchanged. This rigidity could mean this region forms part of the core active-site scaffold that is structurally conserved between 1FKN-INH1 and 1FKN-VER, providing a stable framework for catalytic function.

Active-site loop around ARG307–GLU310 (1FKN-VER-specific): Residues ARG307, VAL309, and GLU310 are annotated as active-site in 1FKN-VER but not in 1FKN-INH1. In 1FKN-INH1, this part of the protein behaves as a flexible surface loop, whereas in 1FKN-VER it appears more engaged (and thus stabilized) in the active site. The RMSF show a clear reduction in flexibility for these residues in 1FKN-VER (Figure 12 and Figure 13). For example, ARG307 (which is in a β-sheet region) has RMSF 0.129 nm in 1FKN-VER (low) compared to 0.215 nm in 1FKN-INH1. VAL309 (loop) drops from 0.203 nm in 1FKN-INH1 to 0.148 nm in 1FKN-VER. The most striking is GLU310 (loop): in 1FKN-INH1 it is highly flexible (0.336 nm, high category) but in 1FKN-VER it is much more restrained (0.228 nm, medium). This inversion of flexibility (a floppy loop in 1FKN-INH1 becoming a steadier active component in 1FKN-VER) strongly suggests that 1FKN-VER’s active site incorporates this loop into its functional architecture. The engagement of ARG307–GLU310 in 1FKN-VER likely introduces hydrophobic interactions (with substrate or other parts of the protein) that dampen their motions. In 1FKN-INH1, lacking those interactions, the same residues freely fluctuate. This difference highlights how the active-site definition in 1FKN-VER extends into a region that is peripheral in 1FKN-INH1, conferring local rigidity to 1FKN-VER’s structure that 1FKN-INH1 does not share.

Active-site region LYS321–ALA323 (1FKN-VER-specific): Later in the sequence, 1FKN-VER includes LYS321 and PHE322 (in a β-sheet) and ALA323 (loop) as part of its active site, whereas 1FKN-INH1 does not (Figure 12 and Figure 13). These residues in 1FKN-VER show low flexibility (RMSF ~0.09–0.16 nm), consistent with their secondary structure (the two in β-sheet are especially rigid). In 1FKN-INH1, the same positions are slightly more flexible (e.g., LYS321 0.192 nm, PHE322 0.108 nm, ALA323 0.128 nm), though still not very high. The modest decrease in mobility in 1FKN-VER suggests these residues become more functionally pinned when part of the active site. LYS321 in 1FKN-VER (0.16 nm) is a bit less flexible than in 1FKN-INH1 (0.19 nm), and PHE322—already quite immobile in 1FKN-INH1—remains so in 1FKN-VER (~0.10 nm). This indicates that 1FKN-VER’s active site extends into a structured region of the protein, incorporating additional rigid elements into the active-site architecture. The presence of these extra active-site residues in 1FKN-VER may contribute to a more pre-organized active-site and structurally stable geometry.

In summary, active-site residues in both proteins generally mirror the expected relationship between structure and flexibility: those in loops or surface-exposed turns have medium flexibility, whereas those embedded in β-sheets or other ordered secondary structures are relatively inflexible. Many active-site residues in 1FKN-INH1 and 1FKN-VER are located on loops (e.g., GLY11–GLN13, TYR71–GLY74, LYS107–ILE110, etc.), which give the active site necessary conformational freedom (medium RMSF) for function. By contrast, a few key catalytic residues reside in the protein core or sheet (e.g., LEU30, ASP32, ILE118, ARG235), showing RMSF on the order of only 0.05–0.14 these likely form an immutable structural core of the active site, ensuring that critical catalytic geometry is maintained. Differences between 1FKN-INH1 and 1FKN-VER active-site dynamics tend to occur in the loop regions at the periphery of the active site: one protein’s active site engages a particular loop that the other does not, leading to that loop being less flexible in the former and more flexible in the latter. Such is the case for the 71–74 loop (highly mobile in 1FKN-INH1, stabilized in 1FKN-VER) and the 307–310 loop (mobile in 1FKN-INH1, stabilized in 1FKN-VER). Meanwhile, shared active-site loops (like 107–111) show similar behavior across both systems, underlining their conserved functional role.

#### 2.7.3. Functional Implications of Flexibility Differences

The comparative flexibility analysis of 1FKN-INH1 and 1FKN-VER highlights subtle yet functionally meaningful differences in their active-site dynamics. In 1FKN-INH1, the active-site loop (TYR71–GLY74) is highly flexible, suggesting a dynamic “lid” that could facilitate substrate access, enhance conformational sampling, and support broader substrate compatibility. In contrast, the same loop in 1FKN-VER is more rigid, indicating a pre-formed binding pocket that may favor substrate specificity and catalytic precision. Despite these differences, both proteins share a rigid catalytic core (e.g., LEU30, ASP32, ILE118), implying that the fundamental catalytic geometry is conserved. Notably, 1FKN-VER includes additional active-site residues (e.g., GLU310, LYS321, PHE322) that are more rigid than in 1FKN-INH1, likely reflecting functional adaptation or engineering toward enhanced stability or specificity. Some residues gain or lose active-site status between variants without major changes in flexibility, suggesting they may play secondary or redundant roles. Additionally, 1FKN-VER features a unique highly flexible segment (residues 159–168) not seen in 1FKN-INH1, possibly representing a mobile tail or surface loop with potential roles in interaction or regulation. Overall, while both proteins appear to preserve core enzymatic function, the observed differences in local flexibility—especially in regions recruited into or excluded from the active site—point to distinct dynamic strategies: 1FKN-INH1 favors adaptability, while 1FKN-VER prioritizes pre-organization and rigidity (Figure 12 and Figure 13). These differences could influence substrate binding kinetics, stability, and specificity, illustrating how dynamic tuning of structural elements supports functional optimization in enzyme variants.

#### 2.7.4. Insights from Inhibitors Interaction Within the Pocket

**Figure 14:**
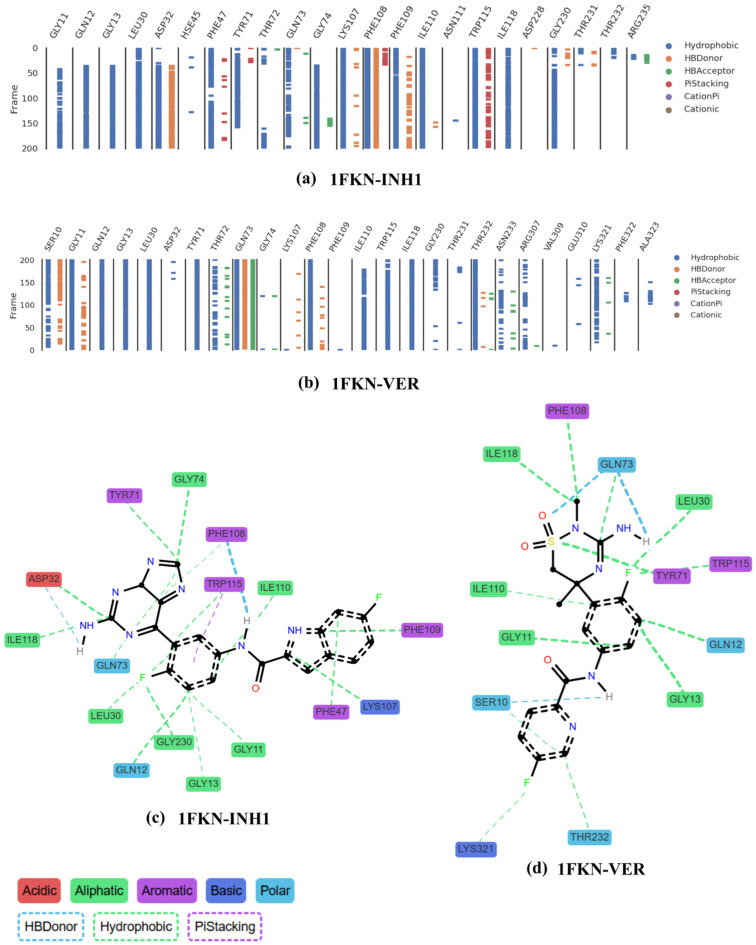
Ligand interactions within the binding pocket over the course of the 200 ns MD simulation. Panels (**a**,**b**) illustrate the types and frequencies of interactions that contribute to the retention of the ligands in the pocket, highlighting both continuous and transient contacts. Panels (**c**,**d**) present 2D interaction plots depicting the interaction profiles between ligand atom moieties and various classes of protein residues, including acidic, aliphatic, aromatic, basic, polar, hydrogen bond donors, hydrophobic regions, and π-stacking interactions. Interaction data were derived from the full 200 ns MD simulation trajectories.

The combined analysis of the 2D interaction diagram and 200 ns MD simulation of the 1FKN-INH1 complex reveals a rich and stable network of non-covalent interactions that contribute to the strong retention and specificity of the inhibitor within the binding pocket (Figure 14). The 2D interaction map highlights the presence of hydrophobic contacts, hydrogen bonding, and π–π stacking interactions, which are further supported by time-resolved interaction data from the simulation trajectory (Figure 14a–d). In 1FKN-INH1, hydrophobic interactions dominate the binding landscape and are consistently observed across residues such as GLY11, GLN12, GLY13, LEU30, ASP32, PHE47, TYR71, GLN73, GLY74, LYS107, PHE108, PHE109, ILE110, TRP115, ILE118, and GLY230 (Figure 14a). These interactions persist throughout the simulation, stabilizing the ligand through van der Waals and nonpolar packing forces.

Hydrogen bonds are formed with key polar residues including ASP32, GLN73, LYS107, PHE108, PHE109, THR72, GLY230, THR231, and ARG235 (Figure 14a, Table 3). Notably, ASP32, PHE108, and PHE109 exhibit highly persistent hydrogen bond donor activity, anchoring the ligand through polar interactions (Figure 14a). PHE108 plays a particularly central role by engaging in multi-modal interactions—acting as a hydrogen bond donor, forming π-stacking interactions, and contributing to the hydrophobic core. TRP115 and PHE47 are also involved in strong π–π stacking interactions with the ligand’s aromatic rings, with TRP115 maintaining these contacts throughout most of the simulation, reinforcing the ligand’s orientation and depth of binding (Figure 14a). Additional hydrogen bond acceptors, such as GLN73, THR72, GLY74, and ARG235, support specificity and ligand positioning. Several aliphatic (ILE110, LEU30) and polar residues (GLN12, GLN73) are spatially positioned to interact with the ligand, further enhancing its stability. While the 2D interaction map (Figure 14c) shows no evidence of cationic or cation–π interactions, the combination of hydrophobic, hydrogen bonding, and π-stacking interactions defines a robust and favorable binding mode. Overall, the cooperative engagement of residues such as ASP32, PHE108, TRP115, and THR231 ensures a tightly bound, conformationally stable ligand, underlining the therapeutic potential of INH1 as a selective binder to the 1FKN-INH1 pocket (Figure 14a,c).

The binding analysis of the 1FKN-VER complex, integrating both static 2D interaction mapping and dynamic MD trajectory data over 200 ns, reveals a well-orchestrated network of hydrophobic contacts, hydrogen bonding, and limited aromatic interactions that stabilize the ligand within the 1FKN-VER binding pocket (Figure 14b). The 2D interaction diagram highlights widespread hydrophobic interactions involving residues such as SER10, GLY11, GLN12, GLY13, LEU30, TYR71, THR72, GLN73, PHE108, ILE110, TRP115, ILE118, THR232, ASN233, ARG307, and LYS321, forming a nonpolar shell around the ligand (Figure 14b,d). These interactions are strongly supported by the MD data, which show persistent hydrophobic engagement particularly with GLY11, GLN12, GLY13, LEU30, TYR71, GLN73, PHE108, ILE118, and THR232 throughout the simulation (Figure 14b).

Crucially, GLN73 serves as a polar anchor, forming two distinct and stable hydrogen bonds with the ligand via its backbone and side chain, corroborated by continuous hydrogen bond acceptor signals in the trajectory. Additional hydrogen bond donors such as SER10, LYS107, PHE108, and THR232 contribute to ligand orientation and stabilization, with GLY11 showing the most persistent donor activity in the time-resolved analysis (Table 4). While π-stacking interactions are limited, TRP115 may provide subtle aromatic contributions (Figure 14b,d). The absence of cationic or cation–π interactions confirms that the binding mechanism is primarily driven by hydrophobic packing and polar contacts rather than electrostatic stabilization. Overall, the ligand VER is stabilized within the 1FKN-VER pocket through a highly cooperative network of nonpolar residues, directional hydrogen bonding, and subtle aromatic engagement, with GLN73, GLY11, ILE110, and TRP115 emerging as key contributors to its binding affinity and pose retention.

The comparative analysis of the 1FKN-INH1 and 1FKN-VER complexes strongly supports the conclusion that INH1 is the superior binder, based on both the quantity and quality of its interactions within the 1FKN-INH1 binding pocket. INH1 engages a greater number of residues through a diverse array of non-covalent forces, including hydrophobic contacts, hydrogen bonds, and π–π stacking interactions, whereas VER primarily relies on hydrophobic and limited polar interactions. Notably, INH1 exhibits frequent and persistent π–stacking with TRP115 and PHE47, which helps lock the ligand into a defined and stable orientation—an advantage not observed with VER, which lacks robust π-stacking altogether. The hydrogen bonding network of INH1 is also significantly more complex, involving critical residues such as ASP32, GLN73, PHE108, PHE109, and ARG235, providing a stabilizing polar framework that is more redundant and thus more resistant to fluctuations. In contrast, VER’s hydrogen bonding is largely dependent on GLN73 and SER10, making it more susceptible to instability. Moreover, PHE108 in the INH1 complex serves as a multi-functional anchor, simultaneously participating in hydrophobic, π-stacking, and hydrogen bond interactions—no comparable multifunctional residue is evident in the 1FKN-VER complex. Structurally, INH1 is deeply embedded within a well-defined binding cavity, surrounded by aromatic and polar residues that enhance encapsulation and shield the ligand from solvent exposure, while VER appears more superficially bound, which may compromise its binding affinity. Altogether, the interaction density, persistence, and structural embedding seen with 1FKN-INH1 suggest a more stable and higher-affinity binding mode, establishing INH1 as the more promising candidate for further optimization and therapeutic development.

To complement the 200 ns ProLIF interaction analysis, we also examined the last snapshot of 200 ns trajectory to check the H-bonds present in the last trajectory. This analysis indicated that 1FKN-INH1 protein complex identified three H-bonds, two salt-bridge interactions, and one π-stacking contact. A H-bond was observed with Gly74, where the protein donates to the ligand (Hydrogen (H)–Acceptor (A) distance = 2.96 Å; Donor (D)–A = 3.94 Å). Two additional H-bonds were detected near Phe108, both involving the ligand as donor toward the same protein acceptor atom, with H–A distances of 1.79 Å and 2.33 Å, D–A distances of 2.72 Å and 3.22 Å, respectively. Two salt-bridge–type contacts were recorded between the ligand’s guanidine group and Asp32 and Asp228, with distances of 4.19 Å and 5.26 Å. A single π-stacking interaction was identified with Trp115, characterized by a centroid distance of 5.37 Å, and an offset of 0.63 Å. For the 1FKN-VER protein complex, two H-bonds were observed. The first interaction involves Ser10, where VER donates a H-bond to the backbone oxygen, with an H–A distance of 2.03 Å, a D–A distance of 2.97 Å. The second H-bond is formed with Gln73, where the protein donates to the ligand’s O2 atom (H–A = 2.52 Å; D–A = 3.49 Å). These two contacts constitute the complete set of H-bonding interactions identified for VER based strictly on the measured geometric parameters.

Thus, conclusively, INH1 forms a more extensive interaction network than VER, including three H-bonds, two salt-bridge contacts, and a Trp-mediated π-stacking interaction, whereas VER engages the protein through only two H-bonds. This richer combination of polar, electrostatic, and aromatic contacts indicates that INH1 is likely to exhibit stronger and more stable binding compared to VER. In addition, we also performed relative binding free energy (RBFE) calculations using the Molecular Mechanics/Poisson–Boltzmann Surface Area (MM/PBSA) method, which showed that VER binds slightly more favorably to BACE-1 than INH1. VER exhibited a total binding free energy of −18.58 ± 3.69 kcal/mol, compared to −16.24 ± 4.31 kcal/mol for INH1. The component-wise energetic terms explain this difference. For VER, the favorable van der Waals energy (VDWAALS = −32.28 ± 3.39 kcal/mol) and electrostatic energy (EEL = −16.49 ± 4.60 kcal/mol) were offset by a moderate solvation free energy (GSOLV = +30.20 ± 4.94 kcal/mol), driven largely by the Poisson–Boltzmann polar solvation term (EPB = +33.99 ± 5.07 kcal/mol) with a smaller nonpolar solvation contribution (ENPOLAR = −3.79 ± 0.28 kcal/mol). For INH1, although the electrostatic energy was stronger (EEL = −20.46 ± 10.44 kcal/mol), the ligand incurred a substantially higher polar solvation penalty (EPB = +44.40 ± 7.52 kcal/mol, total GSOLV = +40.59 ± 7.58 kcal/mol), which reduced its overall binding affinity despite favorable gas-phase interactions (GGAS = −56.83 ± 8.88 kcal/mol).

These energetic trends are consistent with the MD simulations, where VER maintained a more stable pose in the BACE-1 binding pocket over 200 ns. Nevertheless, the ~2.3 kcal/mol difference is modest, indicating that both ligands remain compatible with the same active site, with INH1 retaining several strong electrostatic contacts that support its role as a competitive binder.

## 3. Material and Method

### 3.1. Dataset Collection

A systematic literature review was conducted using PubMed database focusing on keywords like BACE-1 inhibitors and anti-Alzheimer compounds. The database provided data on clinical trials, SARs and biological activity of query compounds. Protein Data Bank (PDB) provided crystal structures of BACE-1 bound to inhibitors for molecular docking studies.

### 3.2. Validation of the Binding Pocket Through Co-Crystal Analysis and Molecular Docking

The co-crystal structures (PDB codes: 4X7I [8], 4YBI [18], 5HU1 [19], 7DCZ [20] and 7MYI [21] of BACE-1 and 7D5B [15] of BACE-2 were retrieved from the PDB and analyzed to identify favorable binding pocket for the design of new inhibitors. The analysis involved the use of PyMOL3.1.6.1 and MOE2008 (Molecular Operating Environment) softwares. These tools were employed to visualize the structures, inspect the conformations and highlight key residues involved in polar/nonpolar interactions between inhibitors and the binding site of targets (Table S3). This integrated approach was also employed for analysis of interaction of docked ligands. It provided a comprehensive understanding of the BACE-1 active site, helping in the identification of favorable binding pockets to guide the rational design and optimization of potential inhibitors.

### 3.3. SAR Study and Design of New BACE-1 Inhibitors

The SAR study and design of new BACE-1 inhibitors were conducted based on literature review [12,13,14,15,16,17] and computational analysis [22,23,24,25]. Published data on known BACE-1 inhibitors were reviewed to identify key structural features and functional groups important for activity. The SAR analysis revealed essential pharmacophoric group, such as hydrogen bond donors/acceptors, hydrophobic interactions, and aromatic regions, that contribute to potent and selective BACE-1 inhibition.

### 3.4. In Silico Study of Designed Molecules Using SwissADME Web Tool 

The precision SAR analysis help to design compounds, which were subsequently evaluated for their toxicity and pharmacokinetic properties, including absorption, distribution, metabolism, and excretion (ADME). The assessment of ADME characteristics was conducted using the SwissADME server, which provided insights into drug-likeness and pharmacokinetic behavior [25].

### 3.5. Molecular Docking Experiment

The protein structure of 1FKN, complexed with an inhibitor molecule, was obtained from the PDB at a resolution of 1.90 Å and prepared using the BIOVIA Discovery Studio Visualizer. Preparation involved the removal of the co-crystallized ligand, unnecessary water molecules, and side chains. The structure was further processed using AutoDock Tools (version 1.5.7) by adding hydrogen atoms, assigning atom types, and calculating Gasteiger charges. Ligands and macromolecules were energy-minimized to ensure stable 3D conformations, and ligands were converted to PDBQT format using AutoDock Tools for molecular docking studies in AutoDock (version 4.2). The binding pocket set using the BIOVIA Discovery Studio Visualizer, and two grid boxes were designed to enclose this site. The grid dimensions were set at 40 × 40 × 40 points in the X, Y, and Z axes, with a spacing of 0.608 Å and center coordinates at X: 27.323, Y: 8.312, Z: 22.755. Screening was performed using AutoDock, and ligands with binding energies below −5 kcal/mol were selected for further analysis. The docking process utilized the Lamarckian Genetic Algorithm (LGA) with 100 genetic algorithm (GA) runs per docking stage, and the results were analyzed to identify ligands with favorable binding affinities and interactions [22]. The predicted docked conformation was compared to the protein-inhibitor co-crystal by calculating the root mean square deviation (RMSD), with values below 2.0 Å considered acceptable. Alignment of co-crystal and docked complexes with PyMOL3.1.6.1 software showed RMSD of 0.864 Å confirming validation of molecular docking protocol.

### 3.6. Molecular Dynamic Simulations

Molecular dynamics (MD) simulations were performed to investigate the structural dynamics and binding stability of protein–small-molecule complexes, which are critical for rational drug design. BACE-1 catalytic domain features a more open and less hydrophobic active site than other human aspartic proteases [26]. The crystal structure used here corresponds to the soluble protease domain (PDB ID: 1FKN; residues 1–385), which lacks C-terminal transmembrane helix yet preserves all key active-site elements (Asp32/Asp228, flap 67–77, loops 8–14 and 154–169). Although membrane interactions contribute to the behavior of full-length BACE-1, the absent helix lies outside the catalytic region, and this soluble domain remains the standard construct for structural and computational inhibitor studies. Thus, we did not include an explicit membrane, allowing a more efficient simulation setup while using the standard soluble BACE-1 construct.

System preparation was carried out using the CHARMM-GUI web server, which streamlines input file generation and system setup [27,28]. Histidine protonation is highly environment-dependent, and its pKa can shift significantly due to local hydrogen-bond networks and electrostatics. To avoid incorrect tautomer assignments, we used the pKa-based H++ webserver at pH 7.4, which evaluates microenvironments using Poisson–Boltzmann electrostatics [29]. Histidine’s predicted to favor Nε-protonation were assigned as HSE, while residues with elevated pKa values or strongly polar surroundings were modeled as the doubly protonated HSP form. Accordingly, HIS45, HIS49, HIS89, HIS145, HIS181, HIS360, and HIS362 were assigned as HSE45, HSE49, HSP89, HSE145, HSE181, HSE360, and HSP362, respectively. This pKa-guided approach is widely used in MD studies [30] and provides a physics-based assignment of histidine protonation states through Poisson–Boltzmann electrostatics.

Disulphide bonds were preserved as indicated in the original PDB structure, including CYS35-CYS155, CYS217–CYS382, and CYS269–CYS319. Ligands were protonated in UCSF Chimera [31] using its built-in AddH algorithm, which assigns hydrogens based on AMBER-style atom typing and idealized valence geometry, with manual inspection to ensure correctness. This procedure satisfies all valences and generates a chemically neutral ligand using standard pKa heuristics. The finalized ligand structures were exported in MOL2 format and processed through CHARMM-GUI’s Ligand Reader & Modeler to produce force-field–compatible topology and parameter files [32].

All simulations were conducted using the CHARMM36 force field [32] and GROMACS 2022.4 [33,34]. Systems were solvated in a TIP3P water box with a 15 Å padding and neutralized with 0.15 M NaCl [35]. The MD simulation workflow included three stages: energy minimization, NVT equilibration, and NPT production. Energy minimization was performed using the steepest descent algorithm for 5000 steps or until the maximum force dropped below 1000 kJ/mol/nm (GROMACS default), which is sufficient to remove steric clashes before thermal and pressure equilibration. Position restraints were applied to both backbone and side chains during this stage. The Verlet cut off scheme was used, with 1.2 nm cut offs for both van der Waals and Coulomb interactions. Long-range electrostatics were calculated using the Particle Mesh Ewald (PME) method [36], and all hydrogen-involving bonds were constrained using the LINCS algorithm [37].

The system was equilibrated under NVT conditions for 125 ps using a 1 fs timestep, maintaining the temperature at 310 K via the Nosé–Hoover thermostat applied separately to solute and solvent groups. Initial velocities were generated from a Maxwell distribution, and protein restraints were maintained during this short NVT phase, which serves as a controlled temperature-relaxation step commonly used in MD workflows. Although brief, the 125 ps NVT period was sufficient to stabilize kinetic energy and temperature, after which pressure and density were equilibrated during the subsequent NPT stage. Following equilibration, the production MD simulation was carried out under NPT conditions for 200 ns using a 2 fs timestep. Temperature and pressure were controlled using the Nosé–Hoover thermostat and the Parrinello–Rahman barostat, respectively [38,39,40]. All position restraints were removed during production, allowing full atomic flexibility. Non-bonded interaction settings and constraints were maintained as in the equilibration stage. Trajectories were saved every 1 ns, and system stability and dynamics were assessed using GROMACS tools by calculating root mean square deviation (RMSD), root mean square fluctuation (RMSF), radius of gyration (Rg), and other structural parameters.

### 3.7. ProLIF’s Interaction Fingerprint, Hydrogen Bonds, and Relative Binding Free Energy (RBFE)

We quantified residue-level interaction patterns using ProLIF, which extracts molecular fingerprints from MD trajectories via MDAnalysis and RDKit. Following standard guidelines, this provided a consistent description of hydrogen bonds, hydrophobic contacts, π-interactions, and electrostatic features relevant for understanding the protein–ligand interaction phenomenon [41]. Additionally, GROMACS hydrogen-bond analysis (gmx hbond) was used to quantify donor–acceptor distances and compute mean H-bond geometries across the trajectories, providing an independent validation of the fingerprint-derived interactions. Furthermore, to compare thermodynamic stability across the ligand, we calculated Relative Binding Free Energies (RBFE) with gmx_MMPBSA [42,43] using the single-trajectory protocol. For calculation, parameters were adapted from the official protein–ligand example tutorial. These RBFE values offer a reliable measure of relative affinity and binding favorability [44].

## 4. Conclusions

BACE-1 is a key drug target for AD. However, BACE-2, a related enzyme, shares similar structure, and its inhibition causes adverse effects. Therefore, selective BACE-1 inhibition is essential. This study focused on structural differences between BACE-1 and BACE-2 active sites. Specifically, targeted hydrophobic regions in BACE-1 using fused and bridged ring systems to enhance binding. A library of 180 compounds was designed and screened for ADME profile. Compounds were filtered based on favorable physicochemical parameters. Molecular docking was used to predict binding affinity to BACE-1. Compound **9.7** (INH1) showed strong binding interactions with key binding residues (ASP 32, Gln 73, ASP228) and hydrophobic residues (Tyr 71, Lys 107, PHE108, TRP115). Molecular dynamics (MD) simulations have validated that the designed inhibitor INH1 showed stable interactions with key binding pocket residues of the BACE-1. Specifically, INH1 maintained stable interactions with the catalytic residue ASP32, which is crucial for BACE-1 enzymatic activity. Additionally, INH1 showed significant hydrophobic interactions with TRP115 and PHE108, residues that contribute to the stability of the ligand-enzyme complex. These interactions were not observed with the reference compound VER during the MD simulations. Moreover, both VER and INH1 maintained favorable interactions with selective BACE-1 residues GLY11 and ILE110.

These findings suggest that compound **9.7** acts as a BACE-1 hit molecule and may be further explored as a potential therapeutic agent for AD.

## Figures and Tables

**Table 1:** Binding pocket residues differences between BACE-1 and BACE-2.

BACE-1 Residue	BACE-2 Residue	Conservation Status	Reported/Possible Effects on Target Activity
ASP32	ASP48	Conserved	Essential for proteolytic activity.
ASP228	ASP241	Conserved	Essential for proteolytic activity.
TYR71	TYR87	Conserved	Involved in substrate binding; contributes to flap dynamics.
ILE110	LEU126	Non-conserved	Ile side chain in BACE-1 has different spatial configuration, affecting the shape and hydrophobicity of the pocket.
ILE126	LEU142	Non-conserved	Alters the topology of the subsite, impacting substrate specificity.
TRP115	TRP131	Conserved	Contributes to the hydrophobic environment; differences may influence inhibitor design.
PHE108	PHE124	Conserved	Both residues are aromatic, but their spatial orientation may differ, affecting interactions with inhibitors.
ASN233	LEU246	Non-conserved	Asn is polar while Leu is nonpolar. Asn may influence hydrogen bonding in BACE-1
PRO70	LYS86	Non-conserved	Pro70 in BACE-1 imparts rigidity to the flap region. In contrast, Lys86 in BACE-2 introduces a positive charge, altering local interactions.

**Table 2:** ADME analysis of filtered compounds using Swiss-ADME tool.

Molecule (R1.R2)	3.4	3.11	6.8	9.11	9.7	10.1	11.11
Consensus Log P	2.76	3.83	3.44	2.88	2.51	2.81	3.2
GI absorption	High	High	High	High	High	High	High
Pgp substrate	Yes	Yes	Yes	No	No	No	No
CYP1A2 inhibitor	No	No	No	No	Yes	Yes	Yes
CYP2C19 inhibitor	No	No	No	No	No	No	No
CYP2C9 inhibitor	No	No	No	Yes	No	Yes	Yes
CYP2D6 inhibitor	No	Yes	Yes	No	No	No	No
CYP3A4 inhibitor	No	Yes	No	Yes	No	No	Yes
Lipinski #violations	0	0	0	0	0	0	0
Ghose #violations	0	0	0	0	0	0	0
Veber #violations	0	0	0	0	0	0	0
Egan #violations	0	0	0	0	0	0	0
Muegge #violations	0	0	0	0	0	0	0
Bioavailability Score	0.55	0.55	0.55	0.55	0.55	0.55	0.55
Synthetic Accessibility	4.66	4.8	4.81	4.25	4.04	4.06	3.91

**Table 3:** Hydrogen Bond Interactions for Ligand INH1 Across 200ns Trajectories.

Pair Id	Donor Residue	Donor Atom	Acceptor Residue	Acceptor Atom	Avg. Distance (nm)
1	THR72	OG1	INH1386	N26	0.91
2	GLN73	NE2	INH1386	O10	0.84
3	GLY74	N	INH1386	N24	0.52
4	ARG235	NH1	INH1386	N25	1.06
5	ARG235	NH1	INH1386	N26	1.06
6	ARG235	NH2	INH1386	N26	1.02
7	INH1386	N8	GLN73	O	0.72
8	INH1386	N8	LYS107	O	0.41
9	INH1386	N8	PHE108	O	0.30
10	INH1386	N17	GLN73	O	0.80
11	INH1386	N17	LYS107	O	0.34
12	INH1386	N17	PHE108	O	0.32
13	INH1386	N17	PHE109	O	0.35
14	INH1386	N17	ILE110	N	0.43
15	INH1386	N29	ASP32	OD1	0.38
16	INH1386	N29	ASP32	OD2	0.39
17	INH1386	N29	ASP228	OD2	0.58
18	INH1386	N29	GLY230	O	0.57
19	INH1386	N29	THR231	OG1	0.68

**Table 4:** Hydrogen Bond Interactions for Ligand VER Across 200ns Trajectories.

Pair Id	Donor Residue	Donor Atom	Acceptor Residue	Acceptor Atom	Avg. Distance (nm)
1	THR72	N	VER386	O13	0.36
2	THR72	OG1	VER386	O13	0.44
3	GLN73	N	VER386	O12	0.56
4	GLN73	N	VER386	O13	0.33
5	GLN73	NE2	VER386	O21	0.8
6	GLY74	N	VER386	O12	0.75
7	THR232	OG1	VER386	N19	0.52
8	THR232	OG1	VER386	N23	0.43
9	ASN233	ND2	VER386	N23	0.71
10	VER386	N18	GLN73	O	0.31
11	VER386	N18	LYS107	O	0.44
12	VER386	N18	PHE108	O	0.38
13	VER386	N19	SER10	O	0.38
14	VER386	N19	GLY11	O	0.37

## Data Availability

Data presented in this study is contained within the article and supplementary material. Further inquiries can be directed to the corresponding author.

## Supplementary Materials

The following supporting information can be downloaded at: https://www.mdpi.com/article/10.3390/ph19010005/s1, Supplementary Information S1 contain Table S1: Physicochemical Properties of reported BACE1 inhibitors; Table S2: Physicochemical Properties of designed BACE1 inhibitors, Table S3: Molecular Docking interactions of reported compounds, Table S4: Molecular docking of 9.7 with BACE-1 and BACE-2. Table S5: Binding Energy of reported BACE-1 compounds, Table S6: Minimum and maximum fluctuating residues range in 1FKN-INH1 and 1FKN-VER, Table S7: Minimum and maximum fluctuating residues in 1FKN-INH1 and 1FKN-VER, Supplementary Information S2: MD simulation video of BACE1-INH1 complex; Supplementary Information S3: MD simulation video of BACE1-VER complex.
