# Supplementary material for: In Silico Investigation of Amidine-Based BACE-1 Inhibitors Against Alzheimer’s Disease: SAR, Pharmacokinetics, Molecular Docking and Dynamic Simulations
Source: Pharmaceuticals (Basel). 2025 Dec 19;19(1):5. doi: 10.3390/ph19010005 (PMC12845190; doi:10.3390/ph19010005)
Supplement: Supplementary file 1 [file pharmaceuticals-19-00005-s001.zip › Supplementary Information S1.pdf]

In Silico Investigation of Amidine based BACE-1 Inhibitors against Alzheimer's Disease: SAR, Pharmacokinetics, Molecular Docking and Dynamic Simulations

Table S1. Physicochemical Properties of reported BACE1 inhibitors

**Compound**	**LY2886721**	**LY2811376**	**Atabecestat**	**Lanabecestat**	**RG7129**	**Elenbecestat**	**Umibecestat**	**LY3202626**	**PF-06751979**	**compound 6**	**compound 22**	**WYETH**	**AMGEN**	**ELI LILY**	**CTS21166**	**BI1181181**	**VERUBECESTAT**
Formula	C18H17F3N4O2S	C15H14F2N4S	C18H14FN5OS	C26H28N4O	C18H14F3N5O2	C19H18F3N5O2S	C19H15ClF7N5O2	C22H20F2N8O2S	C18H19F2N5O3S2	C20H20F2N4O3S	C21H23F2N5O4S2	C23H24N6O	C25H25F2N5O3S	C21H19F3N8O2S	C34H51N5O6S	C23H29FN4O2	C17H17F2N5O3S
Molecular weight	410.41 g/mol	320.36 g/mol	367.40 g/mol	412.53 g/mol	389.33 g/mol	437.44 g/mol	513.80 g/mol	498.51 g/mol	455.50 g/mol	434.46 g/mol	511.57 g/mol	400.48 g/mol	513.56 g/mol	504.49 g/mol	657.86 g/mol	412.50 g/mol	409.41 g/mol
Num. heavy atoms	28	22	26	31	28	30	34	35	30	30	34	30	36	35	46	30	28
Num. arom. heavy atoms	12	12	12	12	12	12	12	18	11	12	12	18	12	17	12	6	12
Fraction Csp3	0.28	0.27	0.11	0.42	0.22	0.37	0.37	0.27	0.44	0.35	0.43	0.26	0.36	0.33	0.53	0.61	0.24
Num. rotatable bonds	4	2	4	2	4	5	6	6	6	4	6	5	6	6	19	2	4
Num. H-bond acceptors	7	5	5	4	8	8	12	9	8	7	9	5	8	10	6	5	7
Num. H-bond donors	3	1	2	1	2	2	2	2	2	2	2	1	1	2	4	2	2
Molar Refractivity	103.64	86.66	101.92	131.51	96.03	109.13	108.83	132.07	113.38	113.41	127.84	123.18	140.97	123.93	183.01	119.65	105.82
TPSA	114.90 Å²	89.46 Å²	129.46 Å²	72.86 Å²	113.39 Å²	127.79 Å²	102.49 Å²	156.81 Å²	165.26 Å²	124.13 Å²	170.31 Å²	97.36 Å²	128.23 Å²	158.50 Å²	162.16 Å²	102.71 Å²	126.13 Å²

**Lipophilicity**																	
Log $P_{o/w}$ (iLOGP)	0	2.28	1.68	3.64	2.35	2.07	2.74	3.16	2.32	2.57	2.37	3.17	3.17	2.29	4.13	2.82	2.13
Log $P_{o/w}$ (XLOGP3)	2.02	2.12	1.97	2.98	1.7	1.39	2.94	1.28	2.38	2.41	1.51	2.49	2.47	1.48	4.43	2.62	0.59
Log $P_{o/w}$ (WLOGP	3.63	3.44	2.88	3.45	3.12	3.35	6.62	1.98	3.23	3.41	3.89	1.82	3.28	2.96	4.78	2.18	2.28
Log $P_{o/w}$ (MLOGP)	2.11	2.47	1.47	3.85	1.38	1.54	2.19	1.28	0.59	1.92	1.37	1.6	1.64	1.48	2.19	2.48	0.84
Log $P_{o/w}$ (SILICOS-IT)	3.52	4.29	3.16	5.94	3.42	3.57	5.35	2.52	3.57	3.74	3.16	4.22	4.38	2.18	4.24	3.27	1.7
Consensus Log $P_{o/w}$	2.26	2.92	2.23	3.97	2.39	2.38	3.97	2.04	2.42	2.81	2.46	2.66	2.99	2.08	3.95	2.67	1.51
**Water Solubility**																	
Log *S* (ESOL)	-3.71	-3.43	-3.44	-4.43	-3.38	-3.39	-4.74	-3.72	-4.04	-4.08	-3.83	-4.01	-4.43	-3.86	-5.65	-4.06	-2.8
Solubility	8.00e-02 mg/ml ; 1.95e-04 mol/l	1.18e-01 mg/ml ; 3.69e-04 mol/l	1.34e-01 mg/ml ; 3.66e-04 mol/l	1.53e-02 mg/ml ; 3.72e-05 mol/l	1.63e-01 mg/ml ; 4.19e-04 mol/l	1.77e-01 mg/ml ; 4.04e-04 mol/l	9.29e-03 mg/ml ; 1.81e-05 mol/l	9.46e-02 mg/ml ; 1.90e-04 mol/l	4.17e-02 mg/ml ; 9.14e-05 mol/l	3.58e-02 mg/ml ; 8.24e-05 mol/l	7.60e-02 mg/ml ; 1.49e-04 mol/l	3.95e-02 mg/ml ; 9.87e-05 mol/l	1.90e-02 mg/ml ; 3.71e-05 mol/l	6.91e-02 mg/ml ; 1.37e-04 mol/l	1.48e-03 mg/ml ; 2.25e-06 mol/l	3.56e-02 mg/ml ; 8.63e-05 mol/l	6.44e-01 mg/ml ; 1.57e-03 mol/l
Class	soluble	Soluble	Soluble	Moderately Soluble	Soluble	Soluble	Moderately Soluble	Soluble	Moderately Soluble	Moderately Soluble	Soluble	Moderately Soluble	Moderately Soluble	Soluble	Moderately soluble	Moderately soluble	Soluble
Log *S* (Ali)	-4.06	-3.63	-4.31	-4.17	-3.7	-3.68	-4.75	-4.17	-5.49	-4.66	-4.69	-4.18	-4.81	-4.42	-7.55	-4.43	-2.81
Solubility	3.57e-02 mg/ml ; 8.70e-05 mol/l	7.51e-02 mg/ml ; 2.35e-04 mol/l	1.78e-02 mg/ml ; 4.85e-05 mol/l	2.77e-02 mg/ml ; 6.70e-05 mol/l	7.83e-02 mg/ml ; 2.01e-04 mol/l	9.20e-02 mg/ml ; 2.10e-04 mol/l	9.05e-03 mg/ml ; 1.76e-05 mol/l	3.35e-02 mg/ml ; 6.72e-05 mol/	1.47e-03 mg/ml ; 3.23e-06 mol/l	9.53e-03 mg/ml ; 2.19e-05 mol/l	1.03e-02 mg/ml ; 2.02e-05 mol/l	2.65e-02 mg/ml ; 6.61e-05 mol/l	8.01e-03 mg/ml ; 1.56e-05 mol/l	1.94e-02 mg/ml ; 3.84e-05 mol/l	1.84e-05 mg/ml ; 2.80e-08 mol/l	1.54e-02 mg/ml ; 3.74e-05 mol/l	6.31e-01 mg/ml ; 1.54e-03 mol/l
Class	Moderately soluble	Soluble	Moderately Soluble	Moderately Soluble	Soluble	Soluble	Moderately Soluble	Moderately Soluble	Moderately Soluble	Moderately Soluble	Moderately Soluble	Moderately Soluble	Moderately Soluble	Moderately Soluble	Poorly soluble	Moderately soluble	Soluble
Log *S* (SILICOS-IT)	-5.97	-5.98	-5.56	-7.66	-6.29	-6.04	-7.92	-6.99	-5.04	-6.22	-6.97	-7.92	-5.78	-6.13	-8.41	-5.05	-5.54
Solubility	4.35e-04	3.38e-04	8.07e-04	9.00e-06	2.02e-04	3.95e-04	6.18e-06 mg/ml ;	5.08e-05 mg/ml ;	4.19e-03	2.62e-04	5.42e-05	4.76e-06	8.56e-04 mg/ml ;	Poorly Soluble	2.55e-06 mg/ml ;	3.63e-03	1.17e-03

	mg/ml ; 1.06e-06 mol/l	mg/ml ; 1.05e-06 mol/l	mg/ml ; 2.20e-06 mol/l	mg/ml ; 2.18e-08 mol/l	mg/ml ; 5.18e-07 mol/l	mg/ml ; 9.04e-07 mol/l	1.20e-08 mol/l	1.02e-07 mol/l	mg/ml ; 9.20e-06 mol/l	mg/ml ; 6.03e-07 mol/l	mg/ml ; 1.06e-07 mol/l	mg/ml ; 1.19e-08 mol/l	1.67e-06 mol/l		3.87e-09 mol/l	mg/ml ; 8.81e-06 mol/l	mg/ml ; 2.86e-06 mol/l
Class	Moderately soluble	Moderately Soluble	Moderately Soluble	Poorly Soluble	Poorly soluble	Poorly Soluble	Poorly Soluble	Poorly Soluble	Moderately Soluble	Poorly Soluble	Poorly Soluble	Poorly Soluble	Moderately Soluble		Poorly soluble	Moderately soluble	Moderately soluble
**Pharmacokinetics**																	
GI absorption	High	High	High	High	High	High	Low	Low	Low	HIGH	Low	High	High	Low	Low	High	High
BBB permeant	No	No	No	Yes	No	No	No	No	No	No	No	No	No	No	No	No	No
P-gp substrate	Yes	Yes	No	Yes	Yes	Yes	Yes	Yes	Yes	Yes	Yes	Yes	Yes	Yes	Yes	Yes	Yes
CYP1A2 inhibitor	No	No	No	No	No	No	No	No	No	No	No	No	No	No	No	No	No
CYP2C19 inhibitor	No	Yes	No	No	Yes	Yes	Yes	No	Yes	Yes	No	No	No	No	No	No	No
CYP2C9 inhibitor	No	No	Yes	No	No	No	No	No	No	No	No	Yes	Yes	No	Yes	No	No
CYP2D6 inhibitor	Yes	No	No	Yes	No	Yes	No	Yes	No	Yes	No	No	No	No	No	Yes	No
CYP3A4 inhibitor	No	No	Yes	Yes	Yes	Yes	Yes	Yes	Yes	Yes	Yes	Yes	Yes	Yes	No	Yes	No
Log Kp (skin permeation)	-7.37 cm/s	-6.75 cm/s	-7.14 cm/s	-6.70 cm/s	-7.47 cm/s	-7.98 cm/s	-7.35 cm/s	-8.43 cm/s	-7.39 cm/s	-7.24 cm/s	8.35 cm/s	-6.98 cm/s	-7.68 cm/s	-8.33 cm/s	-7.17 cm/s	-6.96 cm/s	-8.38 cm/s
**Drug Likeness**																	
Lipinski	Yes;0 violation	Yes; 0 violation	Yes;0 violation	Yes: 0 violation	Yes ; 0 violation	Yes ; 0 violation	Yes; 1 violation: MW>500	Yes; 0 violation	Yes ; 0 violation	Yes; 0 violation	Yes; 1 violation: MW>500	Yes 0 violation	Yes; 1 violation : MW>500	Yes; 1 violation: MW>500	No; 2 violations: MW>500, NorO>10	Yes; 0 violation	Yes; 0 violation
Ghose	Yes	Yes	Yes	No; 1 violation:	Yes	Yes	No; 2 violations: MW>480,	No; 2 violations:	Yes	Yes	No; 1 violation:	Yes	No; 2 violations:	No; 1 violation:	No; 3 violations: MW>480,	Yes	Yes

				MR>130			WLOGP>5.6	MW>480, MR>130			MW>480		MW>480, MR>130	MW>480	MR>130, #atoms>70		
Veber	Yes	Yes	Yes	Yes	Yes	Yes	Yes	No; 1 violation: TPSA>140	No; 1 violation: TPSA>140	Yes	No; 1 violation: TPSA>140	Yes	Yes	No; 1 violation: TPSA>140	No; 2 violations: Rotors>10, TPSA>140	Yes	Yes
Eagan	Yes	Yes	Yes	Yes	Yes	Yes	No; 1 violation: WLOGP>5.88	No; 1 violation: TPSA>131.6	No; 1 violation: TPSA>131.6	Yes	No; 1 violation: TPSA>131.6	Yes	Yes	No; 1 violation: TPSA>131.6	No; 1 violation: TPSA>131.6	Yes	Yes
Muegge	Yes	Yes	Yes	Yes	Yes	Yes	No; 1 violation: H-acc>10	No; 1 violation: TPSA>150	No; 1 violation: TPSA>150	Yes	No; 1 violation: TPSA>150	Yes	Yes	No; 1 violation: TPSA>150	No; 3 violations: MW>600, TPSA>150, Rotors>15	Yes	Yes
Bioavailability Score	0.55	0.55	0.55	0.55	0.55	0.55	0.55	0.55	0.55	0.55	0.55	0.55	0.55	0.55	0.17	0.55	0.55
**Medicinal Chemistry**																	
PAINS	0 alert	0 alert	0 alert	0 alert	0 alert	0 alert	0 alert	0 alert	0 alert	0 alert	0 alert	0 alert	0 alert	O alert	0 alert	0 alert	0 alert
Brenk	0 alert	0 alert	0 alert	1 alert ; triple_bond	0 alert	0 alert	0 alert	0 alert	0 alert	0 alert	0 alert	0 alert	1 alert: triple_bond	0 alert	0 alert	0 alert	0 alert
Leadlikeness	No; 1 violation: MW>350	Yes	No; 1 violation: MW>350	No; 1 violation: MW>350	No; 1 violation: MW>350	No; 1 violation: MW>350	No; 1 violation: MW>350	No; 1 violation: MW>350	No; 1 violation: MW>350	No; 1 violation: MW>350	No; 1 violation: MW>350	No; 1 violation: MW>350	No; 1 violation: MW>350	No; 1 violation: MW>350	No; 3 violations: MW>350, Rotors>7, XLOGP3>3.5	No; 1 violation: MW>350	No; 1 violation: MW>350
Synthetic accessibility	4.3	3.66	3.9	5.85	3.76	4.59	4.55	4.57	4.83	4.61	4.92	4.1	5.2	4.88	5.74	5.87	3.87

Table S2. Physicochemical Properties of designed BACE1 inhibitors

Compound	Canonical SMILES	Formula	MW	#Heavyato	#Aromatic	FractionC	#Rotatabl	#H-bondac	#H-bonddo	MR	TPSA	iLOGP	XLOGP3	WLOGP	MLOGP	Silicos-I	Consensus	ESOLLogS	ESOLSolub	ESOLSolub	ESOLClass	AliLogS	AliSolubi	AliSolubi	AliClass	Silicos-I	Silicos-I	Silicos-I	Silicos-I	GIabsorpt	BBBpermea	Pgpsubstr	CYP1A2inh	CYP2C19in	CYP2C9inh	CYP2D6inh	CYP3A4inh	logKp(cm/	Lipinski#	Ghose#vio	Veber#vio	Egan#viol	Muegge#vi	Bioavaila	PAINS#alle	Brenk#alen	Leadliken	Syntheti c

				ms	heavyatoms	sp3	ebonds	ceptors	nors							TLogP	LogP		ility(mg/ml)	ility(mol/l)			lity(mg/ml)	lity(mol/l)		TLogSw	TSolubility(mg/ml)	TSolubility(mol/l)	TClass	ion	nt	ate	ibitor	hibitor	ibitor	ibitor	ibitor	s)	violations	lations	lations	ations	olations	bilityScore	rts	rts	ess#violations	Accessibility
1.1	FC(C1NCC21CN(C2)[C@@H](c1cc(ccc1F)NC(=O)c1ncc2c(c1)cc(o2)F)N)F	C21H19F4N5O2	449.4	32	15	0.33	6	10	3	113.8	96.42	2.51	2.14	3.26	2.14	3	2.61	-3.93	5.34E-02	1.19E-04	Soluble	-3.8	7.18E-02	1.60E-04	Soluble	-6.69	9.18E-05	2.04E-07	Poorly soluble	High	No	Yes	No	No	No	Yes	Yes	-7.52	0	0	0	0	0	0.55	0	0	1	4.7
1.2	COc1cccc2c1C=C(CO2)C(=O)Nc1ccc(c(c1)[C@H](N1CC2(C1)CNC2C(F)F)N)F	C24H25F3N4O3	474.48	34	12	0.38	7	9	3	126.85	88.85	3.48	2.06	2.62	2.2	3.1	2.69	-3.88	6.27E-02	1.32E-04	Soluble	-3.55	1.32E-01	2.79E-04	Soluble	-6.32	2.29E-04	4.82E-07	Poorly soluble	High	No	Yes	No	No	No	Yes	Yes	-7.73	0	0	0	0	0	0.55	0	0	1	5.16
1.3	FC(C1NCC21CN(C2)[C@@H](c1cc(ccc1F)NC(=O)C1=Cc2c(OC1)ccc(c2)F)N)F	C23H22F4N4O2	462.44	33	12	0.35	6	9	3	120.32	79.62	3.24	2.19	3.17	3.16	3.46	3.04	-3.96	5.07E-02	1.10E-04	Soluble	-3.5	1.48E-01	3.19E-04	Soluble	-6.48	1.53E-04	3.30E-07	Poorly soluble	High	No	Yes	No	No	No	Yes	Yes	-7.57	0	0	0	0	0	0.55	0	0	1	5.01

1.4	FC(C1NCC21CN(C2)[C@@H](c1cc(ccc1F)NC(=O)c1cc2ccc(=O)oc2cc1O)N)F	C23H21F3N4O4	474.43	34	16	0.3	6	10	4	124.34	120.83	2.03	2.22	2.37	2.37	2.73	2.34	-4.13	3.50E-02	7.37E-05	Moderately soluble	-4.39	1.92E-02	4.05E-05	Moderately soluble	-6.51	1.48E-04	3.11E-07	Poorly soluble	High	No	Yes	No	No	No	Yes	Yes	-7.62	0	0	0	0	0	0.55	0	1	1	4.69
1.5	O=C(C1CC2C(C1)OC(O2)(F)F)Nc1ccc(c(c1)[C@H](N1CC2(C1)CNC2C(F)F)N)F	C20H23F5N4O3	462.41	32	6	0.65	6	11	3	108.49	88.85	3.01	1.31	2.83	2.03	2.05	2.25	-3.28	2.45E-01	5.31E-04	Soluble	-2.78	7.74E-01	1.67E-03	Soluble	-4.36	2.02E-02	4.37E-05	Moderately soluble	High	No	Yes	No	No	No	Yes	Yes	-8.19	0	0	0	0	1	0.55	0	0	1	4.99
1.6	FC(C1NCC21CN(C2)[C@@H](c1cc(ccc1F)NC(=O)C1=NC(=O)N(c2c1ccc(c2)F)C)N)F	C24H24F4N6O2	504.48	36	12	0.38	6	10	3	137.86	103.06	2.86	1.26	1.51	1.84	3.15	2.12	-3.61	1.23E-01	2.44E-04	Soluble	-3.02	4.78E-01	9.49E-04	Soluble	-6.59	1.30E-04	2.57E-07	Poorly soluble	High	No	Yes	No	No	No	Yes	Yes	-8.48	1	2	0	0	0	0.55	0	0	1	5.37
1.7	FC(C1NCC21CN(C2)[C@@H](c1cc(ccc1F)NC(=O)c1cc2c([nH]1)cc(cc2)F)N)F	C22H21F4N5O	447.43	32	15	0.32	6	8	4	118.09	86.18	2.84	2.6	3.6	3.15	3.69	3.18	-4.2	2.80E-02	6.27E-05	Moderatelyso	-4.06	3.91E-02	8.73E-05	Moderatelyso	-7.06	3.89E-05	8.69E-08	Poorlysolub	High	No	Yes	No	No	No	Yes	Yes	-7.18	0	0	0	0	0	0.55	0	0	1	4.41

																					luble				luble				le																			
1.8	O=C(C1C=CC2C(C1O)OC(O2)(F)F)Nc1ccc(c(c1)[C@H](N1CC2(C1)CNC2C(F)F)N)F	C21H23F5N4O4	490.42	34	6	0.57	6	12	4	113.98	109.08	2.82	1.12	1.97	1.37	0.99	1.65	-3.32	2.34E-01	4.78E-04	Soluble	-3	4.86E-01	9.91E-04	Soluble	-3.34	2.26E-01	4.61E-04	Soluble	High	No	Yes	No	No	No	Yes	No	-8.5	0	1	0	0	1	0.55	0	1	1	5.69
1.9	FC(C1NCC21CN(C2)[C@@H](c1cc(ccc1F)NC(=O)c1cnn2c1OCC(C2)(F)F)N)F	C20H21F5N6O2	472.41	33	11	0.5	6	11	3	112.64	97.44	2.82	1.03	2.62	2.39	1.79	2.13	-3.27	2.55E-01	5.39E-04	Soluble	-2.67	1.02E+00	2.16E-03	Soluble	-5	4.72E-03	1.00E-05	Moderately soluble	High	No	Yes	No	No	No	Yes	No	-8.45	0	0	0	0	1	0.55	0	0	1	4.89
1.10	FC(c1sc2c(c1)cnc(c2)C(=O)Nc1ccc(c(c1)[C@H](N1CC2(C1)CNC2C(F)F)N)F)F	C22H20F5N5OS	497.48	34	15	0.36	7	10	3	124.52	111.52	3.47	2.97	4.62	3.25	4.77	3.82	-4.66	1.09E-02	2.19E-05	Moderately soluble	-4.98	5.27E-03	1.06E-05	Moderately soluble	-7.02	4.73E-05	9.51E-08	Poorly soluble	High	No	Yes	No	No	No	Yes	Yes	-7.23	0	1	0	0	0	0.55	0	0	1	4.72
1.11	FC(C1NCC21CN(C2)[C@@H](c1cc(ccc1F)NC(=O)c1ccc2c	C23H21F5	480.43	34	12	0.35	6	10	3	120.88	79.62	3.13	3.11	4.29	3.27	3.47	3.45	-4.64	1.09E-02	2.27E-05	Moderate	-4.45	1.70E-02	3.54E-05	Moderate	-6.51	1.48E-04	3.08E-07	Poorlyso	High	No	Yes	No	No	No	Yes	Yes	-7.02	0	1	0	0	0	0.55	0	0	1	5.04

	(c1)C=CC(O2)(F)F)N)F	N4O2																			lysoluble				lysoluble				luble																			
1.12	O=C(C1NC2CC1C1C2OC(O1)(C)C)Nc1ccc(c(c1)[C@H](N1CC2(C1)CNC2C(F)F)N)F	C23H30F3N5O3	481.51	34	6	0.7	6	10	4	127.41	100.88	2.79	0.57	0.74	1.67	1.35	1.42	-2.92	5.80E-01	1.20E-03	Soluble	-2.26	2.64E+00	5.48E-03	Soluble	-4.42	1.84E-02	3.82E-05	Moderately soluble	High	No	Yes	No	No	No	Yes	No	-8.83	0	1	0	0	0	0.55	0	0	1	5.64
2.1	FCC1(N=C(c2c1nc(F)cc2)N)c1cc(ccc1F)NC(=O)c1ncc2c(c1)cc(o2)F	C22H13F4N5O2	455.36	33	21	0.09	5	9	2	112.66	106.4	2.03	2.75	4.83	2.74	5.19	3.51	-4.54	1.32E-02	2.91E-05	Moderately soluble	-4.64	1.04E-02	2.29E-05	Moderately soluble	-9.18	2.99E-07	6.58E-10	Poorly soluble	High	No	Yes	Yes	No	Yes	Yes	Yes	-7.13	0	0	0	0	0	0.55	0	1	1	4.24
2.2	COc1cccc2c1C=C(CO2)C(=O)Nc1ccc(c(c1)C1(CF)N=C(c2c1nc(F)cc2)N)F	C25H19F3N4O3	480.44	35	18	0.16	6	8	2	125.71	98.83	2.87	2.67	4.19	2.78	5.29	3.56	-4.49	1.57E-02	3.27E-05	Moderately solub	-4.4	1.92E-02	4.01E-05	Moderately solub	-8.81	7.46E-07	1.55E-09	Poorly soluble	High	No	Yes	Yes	Yes	Yes	Yes	Yes	-7.33	0	1	0	0	0	0.55	0	1	1	4.56

2.3	FCC1(N=C(c2c1nc(F)cc2)N)c1cc(ccc1F)NC(=O)C1=Cc2c(OC1)ccc(c2)F	C24H16F4N4O2	468.4	34	18	0.12	5	8	2	119.18	89.6	2.74	2.8	4.74	3.75	5.65	3.94	-4.57	1.26E-02	2.69E-05	Moderately soluble	-4.34	2.15E-02	4.59E-05	Moderately soluble	-8.97	4.98E-07	1.06E-09	Poorly soluble	High	No	Yes	Yes	Yes	Yes	Yes	Yes	-7.17	0	0	0	0	0	0.55	0	1	1	4.41
2.4	FCC1(N=C(c2c1nc(F)cc2)N)c1cc(ccc1F)NC(=O)c1cc2ccc(=O)oc2cc1O	C24H15F3N4O4	480.4	35	22	0.08	5	9	3	123.2	130.81	1.82	2.83	3.94	2.97	4.92	3.29	-4.74	8.81E-03	1.83E-05	Moderately soluble	-5.23	2.80E-03	5.82E-06	Moderately soluble	-9	4.82E-07	1.00E-09	Poorly soluble	Low	No	Yes	No	No	Yes	No	No	-7.22	0	1	0	0	0	0.55	0	2	1	4.3
2.5	FCC1(N=C(c2c1nc(F)cc2)N)c1cc(ccc1F)NC(=O)C1CC2C(C1)OC(O2)(F)F	C21H17F5N4O3	468.38	33	12	0.38	5	10	2	107.35	98.83	2.53	1.92	4.4	2.6	4.24	3.14	-3.89	6.00E-02	1.28E-04	Soluble	-3.62	1.13E-01	2.40E-04	Soluble	-6.85	6.59E-05	1.41E-07	Poorly soluble	High	No	Yes	No	No	No	Yes	Yes	-7.79	0	0	0	0	0	0.55	0	1	1	4.9
2.6	FCC1(N=C(c2c1nc(F)cc2)N)c1cc(ccc1F)NC(=O)C1=NCC(=O)N(c2	C25H18F4N6	510.44	37	18	0.16	5	9	2	136.72	113.04	2.31	1.87	3.07	2.43	5.33	3	-4.21	3.13E-02	6.13E-05	Moderately	-3.87	6.96E-02	1.36E-04	Soluble	-9.08	4.24E-07	8.30E-10	Poorly solu	High	No	Yes	No	Yes	Yes	No	Yes	-8.09	1	2	0	0	0	0.55	0	1	1	4.69

	c1ccc(c2)F)C	O2																			soluble								ble																			
2.7	FCC1(N=C(c2c1nc(F)cc2)N)c1cc(ccc1F)NC(=O)c1[nH]c2c(c1)ccc(c2)F	C23H15F4N5O	453.39	33	21	0.09	5	7	3	116.95	96.16	2.42	3.21	5.17	3.74	5.88	4.09	-4.81	6.95E-03	1.53E-05	Moderately soluble	-4.9	5.69E-03	1.25E-05	Moderately soluble	-9.55	1.27E-07	2.80E-10	Poorly soluble	High	No	Yes	Yes	No	Yes	Yes	Yes	-6.79	0	0	0	0	0	0.55	0	1	1	4.2
2.8	FCC1(N=C(c2c1nc(F)cc2)N)c1cc(ccc1F)NC(=O)C1C=CC2C(C1O)OC(O2)(F)F	C22H17F5N4O4	496.39	35	12	0.32	5	11	3	112.84	119.06	2.22	1.73	3.53	1.94	3.18	2.52	-3.93	5.82E-02	1.17E-04	Soluble	-3.85	7.07E-02	1.42E-04	Soluble	-5.83	7.39E-04	1.49E-06	Moderately soluble	High	No	Yes	No	No	No	Yes	Yes	-8.1	0	1	0	0	1	0.55	0	2	1	5.29
2.9	FCC1(N=C(c2c1nc(F)cc2)N)c1cc(ccc1F)NC(=O)c1cnn2c1OCC(C2)(F)F	C21H15F5N6O2	478.37	34	17	0.24	5	10	2	111.5	107.42	2.26	1.64	4.19	2.97	3.98	3.01	-3.88	6.32E-02	1.32E-04	Soluble	-3.51	1.48E-01	3.10E-04	Soluble	-7.49	1.54E-05	3.22E-08	Poorly soluble	High	No	Yes	No	No	Yes	Yes	Yes	-8.05	0	0	0	0	0	0.55	0	1	1	4.32
2.10	FCC1(N=C(c2c1nc(F)cc2)N)c1cc(ccc	C23H1	503.	35	21	0.13	6	9	2	123.	121.5	2.32	3.58	6.19	3.58	6.96	4.52	-5.26	2.74E	5.44E	Moder	-5.82	7.66E	1.52E	Moder	-9.51	1.55E	3.07E	Poorl	Low	No	Yes	Yes	Yes	Yes	No	Yes	-6.83	1	2	0	1	0	0.55	0	1	2	4.3

	1F)NC(=O)c1ncc2c(c1)sc(c2)C(F)F	4F5N5OS	45							38									-03	-06	ately soluble		-04	-06	ately soluble		-07	-10	y soluble																			
2.11	FCC1(N=C(c2c1nc(F)cc2)N)c1cc(ccc1F)NC(=O)c1ccc2c(c1)C=CC(O2)(F)F	C24H15F5N4O2	486.39	35	18	0.12	5	9	2	119.74	89.6	2.65	3.72	5.85	3.85	5.66	4.35	-5.25	2.74E-03	5.63E-06	Moderately soluble	-5.29	2.48E-03	5.09E-06	Moderately soluble	-9	4.83E-07	9.93E-10	Poorly soluble	High	No	Yes	Yes	Yes	Yes	No	Yes	-6.63	0	2	0	0	0	0.55	0	1	2	4.4
2.12	O=C(C1NC2CC1C1C2OC(O1)(C)C)Nc1ccc(c(c1)C1N=C(c2c1nc(F)cc2)N)F	C23H23F2N5O3	455.46	33	12	0.43	4	8	3	121.53	110.86	2.51	1.01	1.55	1.91	2.74	1.94	-3.31	2.26E-01	4.95E-04	Soluble	-2.93	5.38E-01	1.18E-03	Soluble	-6.04	4.14E-04	9.10E-07	Poorly soluble	High	No	Yes	No	No	No	Yes	Yes	-8.36	0	0	0	0	0	0.55	0	1	1	5.3
3.1	FC1C2C1C(CC(=N2)N)c1cc(ccc1F)NC(=O)c1ncc2c(c1)cc(o2)F	C20H15F3N4O2	400.35	29	15	0.25	4	7	2	102.68	93.51	2.2	2.02	4.23	2.68	3.77	2.98	-3.71	7.74E-02	1.93E-04	Soluble	-3.61	9.80E-02	2.45E-04	Soluble	-6.6	9.96E-05	2.49E-07	Poorly soluble	High	No	Yes	No	No	No	Yes	Yes	-7.31	0	0	0	0	0	0.55	0	0	1	4.54
3.2	COc1ccc2c1C=C(CO2)C(=O	C23H	425.	31	12	0.3	5	6	2	115.	85.	2.97	1.94	3.59	3.02	3.86	3.08	-3.	9.39	2.21	Solu	-3.	1.82	4.27	Solu	-6.	2.48	5.83	Poor	High	No	Yes	No	No	No	Yes	Yes	-7.	0	0	0	0	0	0.55	0	0	1	4.98

	)Nc1ccc(c(c1)C1CC(=NC2C1C2F)N)F	21F2N3O3	43							74	94							66	E-02	E-04	ble	37	E-01	E-04	ble	23	E-04	E-07	lysoluble									52										
3.3	FC1C2C1C(CC(=N2)N)c1cc(ccc1F)NC(=O)C1=Cc2c(OC1)ccc(c2)F	C22H18F3N3O2	413.39	30	12	0.27	4	6	2	109.2	76.71	2.93	2.07	4.14	3.73	4.22	3.42	-3.74	7.54E-02	1.82E-04	Soluble	-3.31	2.02E-01	4.90E-04	Soluble	-6.4	1.66E-04	4.01E-07	Poorlysoluble	High	No	Yes	No	No	No	Yes	Yes	-7.35	0	0	0	0	0	0.55	0	0	1	4.83
3.4	FC1C2C1C(CC(=N2)N)c1cc(ccc1F)NC(=O)c1cc2ccc(=O)oc2cc1O	C22H17F2N3O4	425.38	31	16	0.23	4	7	3	113.22	117.92	1.97	2.1	3.34	2.91	3.49	2.76	-3.92	5.13E-02	1.21E-04	Soluble	-4.21	2.64E-02	6.21E-05	Moderatelysoluble	-6.42	1.60E-04	3.77E-07	Poorlysoluble	High	No	Yes	No	No	No	No	No	-7.4	0	0	0	0	0	0.55	0	1	1	4.66
3.5	O=C(C1CC2C(C1)OC(O2)(F)F)Nc1ccc(c(c1)C1CC(=NC2C1C2F)N)F	C19H19F4N3O3	413.37	29	6	0.58	4	8	2	97.37	85.94	2.47	1.19	3.8	2.56	2.81	2.57	-3.04	3.76E-01	9.09E-04	Soluble	-2.59	1.06E+00	2.57E-03	Soluble	-4.28	2.19E-02	5.31E-05	Moderatelysoluble	High	No	Yes	No	No	No	Yes	Yes	-7.98	0	0	0	0	0	0.55	0	0	1	5
3.6	FC1C2C1C(CC(=N2)	C23	455	33	12	0.3	4	7	2	126	100	2.	1.	2.	2.	3.9	2.	-3.	1.8	4.1	Sol	-2.	6.6	1.4	Sol	-6.	1.4	3.0	Poo	Hi	No	Yes	No	No	No	No	Yes	-8.	0	0	0	0	0	0.	0	0	1	5.

	N)c1cc(ccc1F)NC(=O)C1=NCC(=O)N(c2c1ccc(c2)F)C	H20F3N5O2	.43							.75	.15	28	14	48	66		49	39	7E-01	0E-04	uble	84	2E-01	5E-03	uble	51	0E-04	8E-07	rlysoluble	gh								27						55				12
3.7	FC1C2C1C(CC(=N2)N)c1cc(ccc1F)NC(=O)c1cc2c([nH]1)cc(cc2)F	C21H17F3N4O	398.38	29	15	0.24	4	5	3	106.97	83.27	2.64	2.48	4.57	3.71	4.46	3.57	-3.99	4.07E-02	1.02E-04	Soluble	-3.87	5.33E-02	1.34E-04	Soluble	-6.97	4.23E-05	1.06E-07	Poorlysoluble	High	No	Yes	Yes	No	No	Yes	Yes	-6.97	0	0	0	0	0	0.55	0	0	1	4.49
3.8	O=C(C1C=CC2C(C1O)OC(O2)(F)F)Nc1ccc(c(c1)C1CC(=NC2C1C2F)N)F	C20H19F4N3O4	441.38	31	6	0.5	4	9	3	102.87	106.17	2.33	1	2.94	1.9	1.75	1.98	-3.09	3.62E-01	8.21E-04	Soluble	-2.82	6.70E-01	1.52E-03	Soluble	-3.25	2.45E-01	5.56E-04	Soluble	High	No	Yes	No	No	No	Yes	Yes	-8.28	0	0	0	0	0	0.55	0	1	1	5.42
3.9	FC1C2C1C(CC(=N2)N)c1cc(ccc1F)NC(=O)c1cnn2c1OCC(C2)(F)F	C19H17F4N5O2	423.36	30	11	0.42	4	8	2	101.53	94.53	2.46	0.91	3.59	2.91	2.56	2.48	-3.05	3.81E-01	9.01E-04	Soluble	-2.48	1.40E+00	3.31E-03	Soluble	-4.92	5.13E-03	1.21E-05	Moderatelysoluble	High	No	Yes	No	No	No	Yes	No	-8.24	0	0	0	0	0	0.55	0	0	1	4.74
3.10	FC1C2C1C(CC(=N2)N)c1cc(ccc1F)NC(=O)c1ncc2c	C21H16F	448.44	31	15	0.29	5	7	2	113.4	108.61	2.37	2.85	5.59	3.81	5.53	4.03	-4.44	1.61E-	3.60E-	Moderat	-4.79	7.28E-	1.62E-	Moderat	-6.94	5.13E-	1.14E-	Poorlys	Low	No	Yes	No	No	No	Yes	Yes	-7.01	0	0	0	0	0	0.55	0	0	1	4.64

	(c1)sc(c2)C(F)F	4N4OS																	02	05	ely soluble		03	05	ely soluble		05	07	oluble																							
3.11	NC1=NC2C(C2C(C1)c1cc(ccc1F)NC(=O)c1ccc2c(c1)C=CC(O2)(F)F)F	C22H17F4N3O2	431.38	31	12	0.27	4	7	2	109.77	76.71	2.82	2.99	5.26	3.83	4.23	3.83	-4.42	1.64E-02	3.80E-05	Moderately soluble	-4.26	2.34E-02	5.43E-05	Moderately soluble	-6.43	1.61E-04	3.72E-07	Poorly soluble	High	No	Yes	No	No	No	Yes	Yes	-6.81	0	0	0	0	0	0.55	0	0	1	4.8
3.12	O=C(C1NC2CC1C1C2OC(O1)(C)C)Nc1ccc(c(c1)C1CC(=NC2C1C2F)N)F	C22H26F2N4O3	432.46	31	6	0.64	4	7	3	116.29	97.97	2.52	0.45	1.71	2.19	2.11	1.8	-2.68	8.95E-01	2.07E-03	Soluble	-2.08	3.63E+00	8.40E-03	Soluble	-4.34	2.00E-02	4.61E-05	Moderately soluble	High	No	Yes	No	No	No	Yes	No	-8.62	0	0	0	0	0	0.55	0	0	1	5.5
4.1	NC1=NC2CC(C1c1cc(ccc1F)NC(=O)c1ncc3c(c1)cc(o3)F)C(C2)(C)C	C23H22F2N4O2	424.44	31	15	0.35	4	6	2	116.79	93.51	2.6	3.46	4.89	3.21	4.6	3.75	-4.75	7.63E-03	1.80E-05	Moderately solub	-5.11	3.33E-03	7.85E-06	Moderately solub	-7.58	1.13E-05	2.66E-08	Poorly soluble	High	No	Yes	No	No	No	Yes	Yes	-6.43	0	0	0	0	0	0.55	0	0	1	5.34

																					le				le																								
4.2	COc1cccc2c1C=C(CO2)C(=O)Nc1ccc(c(c1)C1C(=NC2CC1C(C)(C)C2)N)F	C26H28FN3O3	449.52	33	12	0.38	5	5	2	129.85	85.94	3.72	3.38	4.25	3.53	4.71	3.92	-4.7	9.06E-03	2.02E-05	Moderately soluble	-4.86	6.16E-03	1.37E-05	Moderately soluble	-7.2	2.81E-05	6.25E-08	Poorly soluble	High	No	Yes	No	Yes	Yes	Yes	Yes	-6.64	0	0	0	0	0	0.55	0	0	1	5.78
4.3	Fc1ccc2c(c1)C=C(CO2)C(=O)Nc1ccc(c(c1)C1C(=NC2CC1C(C)(C)C2)N)F	C25H25F2N3O2	437.48	32	12	0.36	4	5	2	123.31	76.71	3.48	3.51	4.8	4.24	5.07	4.22	-4.78	7.31E-03	1.67E-05	Moderately soluble	-4.8	6.86E-03	1.57E-05	Moderately soluble	-7.37	1.88E-05	4.30E-08	Poorly soluble	High	No	Yes	No	No	Yes	Yes	Yes	-6.48	1	0	0	0	0	0.55	0	0	2	5.63
4.4	NC1=NC2CC(C1c1cc(ccc1F)NC(=O)c1cc3cc(=O)oc3cc1O)C(C2)(C)C	C25H24FN3O4	449.47	33	16	0.32	4	6	3	127.33	117.92	2.3	3.54	4	3.43	4.33	3.52	-4.95	5.02E-03	1.12E-05	Moderately soluble	-5.7	8.95E-04	1.99E-06	Moderately soluble	-7.39	1.81E-05	4.04E-08	Poorly soluble	High	No	Yes	No	No	No	No	No	-6.53	0	0	0	0	0	0.55	0	1	2	5.42
4.5	O=C(C1CC2C(C1)OC(O2)(F)F)Nc1ccc(c(c1)C	C22H26	437.46	31	6	0.64	4	7	2	111.48	85.94	3	2.63	4.46	3.1	3.64	3.36	-4.09	3.57E-	8.16E-	Modera	-4.09	3.60E-	8.22E-	Modera	-5.25	2.48E-	5.68E-	Modera	High	No	Yes	No	No	No	Yes	Yes	-7.1	0	0	0	0	0	0.55	0	0	1	5.83

	1C(=NC2CC1C(C)(C)C2)N)F	F3N3O3																	02	05	tely soluble		02	05	tely soluble		03	06	tely soluble																			
4.6	Fc1ccc2c(c1)N(C)C(=O)CN=C2C(=O)Nc1ccc(c(c1)C1C(=NC2CC1C(C)(C)C2)N)F	C26H27F2N5O2	479.52	35	12	0.38	4	6	2	140.86	100.15	2.79	2.58	3.14	3.16	4.75	3.28	-4.43	1.79E-02	3.73E-05	Moderately soluble	-4.33	2.23E-02	4.66E-05	Moderately soluble	-7.48	1.59E-05	3.32E-08	Poorly soluble	High	No	Yes	No	No	No	No	Yes	-7.39	0	1	0	0	0	0.55	0	0	1	5.96
4.7	Fc1ccc2c(c1)[nH]c(c2)C(=O)Nc1ccc(c(c1)C1C(=NC2CC1C(C)(C)C2)N)F	C24H24F2N4O	422.47	31	15	0.33	4	4	3	121.08	83.27	2.75	3.92	5.23	4.23	5.3	4.29	-5.02	4.01E-03	9.48E-06	Moderately soluble	-5.37	1.81E-03	4.29E-06	Moderately soluble	-7.95	4.78E-06	1.13E-08	Poorly soluble	High	No	Yes	Yes	No	Yes	Yes	Yes	-6.09	1	0	0	0	0	0.55	0	0	2	5.24
4.8	O=C(C1C=CC2C(C1O)OC(O2)(F)F)Nc1ccc(c(c1)C1C(=NC2CC1C(C)(C)C2)N)F	C23H26F3N3O4	465.47	33	6	0.57	4	8	3	116.98	106.17	2.74	2.44	3.59	2.43	2.58	2.76	-4.13	3.42E-02	7.35E-05	Moderately solu	-4.31	2.27E-02	4.87E-05	Moderately solu	-4.22	2.79E-02	5.98E-05	Moderately solu	High	No	Yes	No	No	No	Yes	Yes	-7.41	0	0	0	0	0	0.55	0	1	1	6.31

4.9	NC1=NC2CC(C1c1cc(ccc1F)NC(=O)c1cnn3c1OCC(C3)(F)F)C(C2)(C)C	$C_{22}H_{24}F_3N_5O_2$	447.45	32	11	0.5	4	7	2	115.64	94.53	2.72	2.35	4.25	3.45	3.38	3.23	-4.09	3.68E-02	8.22E-05	Moderately soluble	-3.97	4.74E-02	1.06E-04	Soluble	-5.89	5.80E-04	1.30E-06	Moderately soluble	High	No	Yes	No	No	No	Yes	Yes	-7.36	0	0	0	0	0	0.55	0	0	1	5.54
4.10	NC1=NC2CC(C1c1cc(ccc1F)NC(=O)c1ncc3c(c1)sc(c3)C(F)F)C(C2)(C)C	$C_{24}H_{23}F_3N_4OS$	472.53	33	15	0.38	5	6	2	127.51	108.61	2.64	4.29	6.25	4.34	6.36	4.78	-5.48	1.57E-03	3.32E-06	Moderately soluble	-6.28	2.46E-04	5.20E-07	Poorly soluble	-7.91	5.81E-06	1.23E-08	Poorly soluble	Low	No	Yes	No	No	Yes	No	Yes	-6.14	1	1	0	1	0	0.55	0	0	2	5.43
4.11	O=C(c1ccc2c(c1)C=CC(O2)(F)F)Nc1ccc(c(c1)C1C(=NC2CC1C(C)(C)C2)N)F	$C_{25}H_{24}F_3N_3O_2$	455.47	33	12	0.36	4	6	2	123.88	76.71	3.25	4.43	5.92	4.34	5.07	4.6	-5.46	1.58E-03	3.47E-06	Moderately soluble	-5.76	7.93E-04	1.74E-06	Moderately soluble	-7.4	1.82E-05	4.00E-08	Poorly soluble	High	No	Yes	No	No	Yes	No	Yes	-5.93	1	1	0	1	0	0.55	0	0	2	5.61
4.12	O=C(C1NC2CC1C1C2OC(O1)(C)C)	$C_{25}H_3$	456.	33	6	0.68	4	6	3	130.4	97.97	3.12	1.89	2.37	2.71	2.95	2.61	-3.73	8.46E	1.85E	Solub	-3.57	1.23E	2.69E	Solub	-5.31	2.26E	4.94E	Moder	High	No	Yes	No	No	No	Yes	Yes	-7.74	0	1	0	0	0	0.55	0	0	1	6.38

	Nc1ccc(c(c1)C1C(=NC2CC1C(C)(C)C2)N)F	3FN4O3	55																-02	-04	le		-01	-04	le		-03	-06	ately soluble																			
5.1	Fc1cc2c(o1)cnc(c2)C(=O)Nc1cc(c(c1)c1nc(N)c2c(c1)cc(o2)F)F	C21H11F3N4O3	424.33	31	24	0	4	8	2	105.5	107.18	3.24	4.04	5.97	1.94	4.39	3.92	-5.32	2.01E-03	4.73E-06	Moderately soluble	-5.99	4.30E-04	1.01E-06	Moderately soluble	-8.85	6.06E-07	1.43E-09	Poorly soluble	Low	No	Yes	Yes	No	Yes	Yes	Yes	-6.02	0	1	0	1	0	0.55	0	0	2	3.24
5.2	COc1cccc2c1C=C(CO2)C(=O)Nc1ccc(c(c1)c1nc(N)c2c(c1)cc(o2)F)F	C24H17F2N3O4	449.41	33	21	0.08	5	7	2	118.56	99.61	3.56	3.96	5.33	2.54	4.49	3.97	-5.26	2.46E-03	5.47E-06	Moderately soluble	-5.75	7.95E-04	1.77E-06	Moderately soluble	-8.47	1.51E-06	3.36E-09	Poorly soluble	High	No	Yes	Yes	Yes	Yes	Yes	Yes	-6.23	0	0	0	0	0	0.55	0	0	2	3.79
5.3	Fc1ccc2c(c1)C=C(CO2)C(=O)Nc1ccc(c(c1)c1nc(N)c2c(c1)cc(o2)F)F	C23H14F3N3O3	437.37	32	21	0.04	4	7	2	112.03	90.38	3.33	4.09	5.88	3.23	4.84	4.28	-5.35	1.95E-03	4.47E-06	Moderately sol	-5.69	8.86E-04	2.03E-06	Moderately sol	-8.64	1.01E-06	2.31E-09	Poorly soluble	High	No	Yes	Yes	Yes	Yes	No	Yes	-6.06	0	1	0	0	0	0.55	0	0	2	3.65

																					uble				uble																							
5.4	Fc1cc2c(o1)c(N)nc(c2)c1cc(ccc1F)NC(=O)c1cc2ccc(=O)oc2c1O	C23H13F2N3O5	449.36	33	25	0	4	8	3	116.04	131.59	2.43	4.12	5.08	2.18	4.12	3.58	-5.52	1.36E-03	3.03E-06	Moderately soluble	-6.59	1.16E-04	2.57E-07	Poorly soluble	-8.66	9.76E-07	2.17E-09	Poorly soluble	Low	No	No	No	No	Yes	No	No	-6.12	0	0	0	0	0	0.55	0	1	2	3.43
5.5	O=C(C1CC2C(C1)OC(O2)(F)F)Nc1ccc(c(c1)c1cc2cc(oc2c(n1)N)F)F	C20H15F4N3O4	437.34	31	15	0.3	4	9	2	100.2	99.61	2.94	3.21	5.54	2.06	3.44	3.44	-4.67	9.40E-03	2.15E-05	Moderately soluble	-4.97	4.64E-03	1.06E-05	Moderately soluble	-6.52	1.33E-04	3.05E-07	Poorly soluble	High	No	Yes	No	No	No	Yes	Yes	-6.69	0	0	0	0	0	0.55	0	0	1	4.35
5.6	Fc1ccc2c(c1)N(C)C(=O)CN=C2C(=O)Nc1ccc(c(c1)c1nc(N)c2c(c1)cc(o2)F)F	C24H16F3N5O3	479.41	35	21	0.08	4	8	2	129.57	113.82	2.48	3.16	4.21	2.19	4.52	3.31	-4.98	4.98E-03	1.04E-05	Moderately soluble	-5.22	2.89E-03	6.02E-06	Moderately soluble	-8.75	8.56E-07	1.79E-09	Poorly soluble	High	No	Yes	No	Yes	Yes	No	Yes	-6.98	0	0	0	0	0	0.55	0	0	1	3.96
5.7	Fc1ccc2c(c1)[nH]c(c2)C(=O)N	C22H	422.	31	24	0	4	6	3	109.	96.	2.45	4.5	6.31	2.95	5.08	4.26	-5.6	1.05	2.50	Mode	-6.	2.34	5.54	Poor	-9.	2.57	6.08	Poor	Low	No	Yes	Yes	Yes	No	Yes	Yes	-5.	0	1	0	1	0	0.55	0	0	2	3.36

	c1ccc(c(c1)c1nc(N)c2c(c1)cc(o2)F)F	13F3N4O2	36							79	94								E-03	E-06	rately soluble	26	E-04	E-07	ly soluble	22	E-07	E-10	ly soluble									68										
5.8	O=C(C1C=CC2C(C1O)OC(O2)(F)F)Nc1ccc(c(c1)c1cc2cc(oc2c(n1)N)F)F	C21H15F4N3O5	465.35	33	15	0.24	4	10	3	105.69	119.84	2.45	3.02	4.67	1.41	2.37	2.78	-4.7	9.28E-03	1.99E-05	Moderately soluble	-5.2	2.92E-03	6.29E-06	Moderately soluble	-5.49	1.50E-03	3.21E-06	Moderately soluble	Low	No	Yes	No	No	No	Yes	Yes	-6.99	0	0	0	0	0	0.55	0	1	1	4.84
5.9	Fc1oc2c(c1)cc(nc2N)c1cc(ccc1F)NC(=O)c1cnn2c1OCC(C2)(F)F	C20H13F4N5O3	447.34	32	20	0.15	4	9	2	104.35	108.2	2.74	2.93	5.33	2.17	3.18	3.27	-4.66	9.83E-03	2.20E-05	Moderately soluble	-4.86	6.12E-03	1.37E-05	Moderately soluble	-7.16	3.12E-05	6.97E-08	Poorly soluble	High	No	Yes	Yes	No	Yes	Yes	Yes	-6.95	0	0	0	0	0	0.55	0	0	1	3.53
5.10	Fc1oc2c(c1)cc(nc2N)c1cc(ccc1F)NC(=O)c1ncc2c(c1)sc(c2)C(F)F	C22H12F4N4O	472.41	33	24	0.05	5	8	2	116.23	122.28	2.69	4.88	7.33	2.79	6.15	4.77	-6.05	4.20E-04	8.88E-07	Poorly solub	-7.18	3.10E-05	6.56E-08	Poorly solub	-9.18	3.12E-07	6.61E-10	Poorly solub	Low	No	Yes	No	Yes	Yes	Yes	Yes	-5.72	0	1	0	1	0	0.55	0	0	2	3.5

		2S																			le				le				le																			
5.11	Fc1oc2c(c1)cc(nc2N)c1cc(ccc1F)NC(=O)c1ccc2c(c1)C=CC(O2)(F)F	C23H13F4N3O3	455.36	33	21	0.04	4	8	2	112.59	90.38	3.14	5.01	7	3.07	4.85	4.61	-6.03	4.28E-04	9.41E-07	Poorly soluble	-6.65	1.02E-04	2.25E-07	Poorly soluble	-8.67	9.78E-07	2.15E-09	Poorly soluble	Low	No	No	Yes	Yes	Yes	No	No	-5.52	0	1	0	1	1	0.55	0	0	2	3.63
5.12	O=C(C1NC2CC1C1C2OC(O1)(C)C)Nc1ccc(c(c1)c1cc2cc(oc2c(n1)N)F)F	C23H22F2N4O4	456.44	33	15	0.39	4	8	3	119.12	111.64	3.13	2.47	3.45	1.7	2.73	2.7	-4.3	2.30E-02	5.03E-05	Moderately soluble	-4.46	1.59E-02	3.48E-05	Moderately soluble	-6.58	1.21E-04	2.66E-07	Poorly soluble	High	No	Yes	No	No	No	Yes	Yes	-7.33	0	0	0	0	0	0.55	0	0	1	4.96
6.1	Fc1ccc2c(c1)nc(c(c2)c1cc(ccc1F)NC(=O)c1ncc2c(c1)cc(o2)F)N	C23H13F3N4O2	434.37	32	25	0	4	7	2	113.24	94.04	2.72	4.6	6.37	3.13	5	4.37	-5.75	7.81E-04	1.80E-06	Moderately soluble	-6.3	2.18E-04	5.02E-07	Poorly soluble	-9.62	1.04E-07	2.39E-10	Poorly soluble	Low	No	Yes	Yes	Yes	Yes	Yes	Yes	-5.68	0	1	0	1	0	0.55	0	0	2	3.36
6.2	COc1cccc2c1C=C(CO2)C(=O)Nc1ccc(c(c1)c1cc2ccc(cc2nc1N)F)F	C26H19F2N3	459.44	34	22	0.08	5	6	2	126.29	86.47	3.56	4.53	5.73	3.71	5.11	4.53	-5.69	9.35E-04	2.04E-06	Moderately	-6.07	3.93E-04	8.55E-07	Poorly solu	-9.25	2.59E-07	5.64E-10	Poorly solu	High	No	Yes	Yes	Yes	Yes	Yes	Yes	-5.89	0	1	0	0	0	0.55	0	0	2	3.74

		O3																			soluble				ble				ble																			
6.3	Fc1ccc2c(c1)C=C(CO2)C(=O)Nc1ccc(c(c1)c1cc2ccc(cc2nc1N)F)F	C25H16F3N3O2	447.41	33	22	0.04	4	6	2	119.76	77.24	3.53	4.66	6.28	4.42	5.46	4.87	-5.78	7.44E-04	1.66E-06	Moderately soluble	-6.01	4.38E-04	9.80E-07	Poorly soluble	-9.41	1.73E-07	3.86E-10	Poorly soluble	High	No	Yes	Yes	Yes	Yes	No	No	-5.72	1	1	0	1	0	0.55	0	0	2	3.6
6.4	Fc1ccc2c(c1)nc(c(c2)c1cc(ccc1F)NC(=O)c1cc2ccc(=O)oc2cc1O)N	C25H15F2N3O4	459.4	34	26	0	4	7	3	123.78	118.45	2.26	4.69	5.48	3.35	4.73	4.1	-5.94	5.22E-04	1.14E-06	Moderately soluble	-6.91	5.71E-05	1.24E-07	Poorly soluble	-9.44	1.67E-07	3.64E-10	Poorly soluble	Low	No	No	No	Yes	No	No	No	-5.77	0	0	0	0	0	0.55	0	1	2	3.33
6.5	O=C(C1CC2C(C1)OC(O2)(F)F)Nc1ccc(c(c1)c1cc2ccc(cc2nc1N)F)F	C22H17F4N3O3	447.38	32	16	0.27	4	8	2	107.93	86.47	3.05	3.78	5.94	3.25	4.04	4.01	-5.1	3.54E-03	7.92E-06	Moderately soluble	-5.29	2.30E-03	5.13E-06	Moderately soluble	-7.29	2.28E-05	5.11E-08	Poorly soluble	High	No	Yes	No	No	No	Yes	Yes	-6.35	0	1	0	1	0	0.55	0	0	2	4.16

6.6	Fc1ccc2c(c1)N(C)C(=O)CN=C2C(=O)Nc1ccc(c(c1)c1cc2ccc(cc2nc1N)F)F	C26H18F3N5O2	489.45	36	22	0.08	4	7	2	137.3	100.68	2.98	3.73	4.62	3.35	5.14	3.96	-5.41	1.89E-03	3.87E-06	Moderately soluble	-5.54	1.42E-03	2.91E-06	Moderately soluble	-9.52	1.47E-07	3.00E-10	Poorly soluble	High	No	Yes	No	Yes	Yes	No	Yes	-6.64	0	2	0	0	0	0.55	0	0	2	3.91
6.7	Fc1ccc2c(c1)nc(c(c2)c1cc(ccc1F)NC(=O)c1[nH]c2c(c1)ccc(c2)F)N	C24H15F3N4O	432.4	32	25	0	4	5	3	117.53	83.8	3.16	5.07	6.71	4.15	5.7	4.96	-6.03	4.04E-04	9.35E-07	Poorly soluble	-6.57	1.16E-04	2.68E-07	Poorly soluble	-9.99	4.40E-08	1.02E-10	Poorly soluble	Low	No	Yes	Yes	Yes	No	Yes	Yes	-5.34	0	1	0	1	1	0.55	0	0	2	3.04
6.8	O=C(C1C=CC2C(C1O)OC(O2)(F)F)Nc1ccc(c(c1)c1cc2ccc(cc2nc1N)F)F	C23H17F4N3O4	475.39	34	16	0.22	4	9	3	113.42	106.7	2.98	3.59	5.08	2.58	2.98	3.44	-5.13	3.50E-03	7.36E-06	Moderately soluble	-5.52	1.44E-03	3.04E-06	Moderately soluble	-6.27	2.56E-04	5.38E-07	Poorly soluble	High	No	Yes	No	No	No	Yes	No	-6.65	0	0	0	0	0	0.55	0	1	2	4.81
6.9	Fc1ccc2c(c1)nc(c(c2)c1cc(ccc1F)NC(=O)c1cnn2c1OCC(C2)(F)F)N	C22H15F4N5O2	457.38	33	21	0.14	4	8	2	112.09	95.06	3.01	3.5	5.74	3.35	3.78	3.88	-5.09	3.74E-03	8.17E-06	Moderatelyso	-5.18	3.03E-03	6.62E-06	Moderatelyso	-7.93	5.34E-06	1.17E-08	Poorlysolub	High	No	Yes	Yes	Yes	Yes	Yes	Yes	-6.61	0	1	0	0	0	0.55	0	0	1	3.44

																					luble				luble				le																			
6.10	Fc1ccc2c(c1)nc(c(c2)c1cc(ccc1F)NC(=O)c1ncc2c(c1)sc(c2)C(F)F)N	C24H14F4N4OS	482.45	34	25	0.04	5	7	2	123.96	109.14	3	5.44	7.74	3.98	6.76	5.38	-6.47	1.63E-04	3.37E-07	Poorly soluble	-7.49	1.57E-05	3.25E-08	Poorly soluble	-9.96	5.35E-08	1.11E-10	Poorly soluble	Low	No	Yes	Yes	Yes	No	Yes	Yes	-5.38	0	2	0	1	1	0.55	0	0	2	3.38
6.11	Fc1ccc2c(c1)nc(c(c2)c1cc(ccc1F)NC(=O)c1ccc2c(c1)C=CC(O2)(F)F)N	C25H15F4N3O2	465.4	34	22	0.04	4	7	2	120.32	77.24	3.44	5.58	7.4	4.25	5.47	5.23	-6.46	1.63E-04	3.50E-07	Poorly soluble	-6.96	5.06E-05	1.09E-07	Poorly soluble	-9.44	1.67E-07	3.60E-10	Poorly soluble	Low	No	No	Yes	Yes	No	No	No	-5.18	1	1	0	1	1	0.55	0	0	2	3.6
6.12	O=C(C1NC2CC1C1C2OC(O1)(C)C)Nc1ccc(c(c1)c1cc2ccc(cc2nc1N)F)F	C25H24F2N4O3	466.48	34	16	0.36	4	7	3	126.85	98.5	3.15	3.04	3.86	2.86	3.35	3.25	-4.73	8.65E-03	1.86E-05	Moderately soluble	-4.77	7.84E-03	1.68E-05	Moderately soluble	-7.35	2.08E-05	4.46E-08	Poorly soluble	High	No	Yes	No	No	No	Yes	Yes	-6.99	0	0	0	0	0	0.55	0	0	1	4.82
7.1	N=C1NC(c2cc(ccc2F)NC(=O)c2ncc3c(c2)cc(o3)F)C2C(C1CC2)(C)C	C23H22F2N4	424.44	31	15	0.35	4	6	3	117.38	91.01	2.8	3.87	4.98	3.21	4.49	3.87	-5	4.21E-03	9.91E-06	Moderately s	-5.48	1.41E-03	3.32E-06	Moderately s	-7.99	4.38E-06	1.03E-08	Poorly solub	High	No	Yes	No	No	No	Yes	Yes	-6.14	0	0	0	0	0	0.55	0	1	2	5.07

		O2																			oluble				oluble				le																			
7.2	COc1cccc2c1C=C(CO2)C(=O)Nc1ccc(c(c1)C1NC(=N)C2C(C1CC2)(C)C)F	C26H28FN3O3	449.52	33	12	0.38	5	5	3	130.44	83.44	3.67	3.79	4.34	3.53	4.6	3.99	-4.95	5.00E-03	1.11E-05	Moderately soluble	-5.24	2.61E-03	5.80E-06	Moderately soluble	-7.61	1.09E-05	2.43E-08	Poorly soluble	High	No	Yes	No	No	Yes	Yes	Yes	-6.35	0	1	0	0	0	0.55	0	1	2	5.5
7.3	N=C1NC(c2cc(ccc2F)NC(=O)C2=Cc3c(OC2)ccc(c3)F)C2C(C1CC2)(C)C	C25H25F2N3O2	437.48	32	12	0.36	4	5	3	123.9	74.21	3.51	3.92	4.89	4.24	4.95	4.3	-5.04	4.03E-03	9.22E-06	Moderately soluble	-5.18	2.91E-03	6.65E-06	Moderately soluble	-7.78	7.29E-06	1.67E-08	Poorly soluble	High	No	Yes	Yes	No	Yes	No	Yes	-6.19	1	0	0	0	0	0.55	0	1	2	5.36
7.4	N=C1NC(c2cc(ccc2F)NC(=O)c2cc3ccc(=O)oc3cc2O)C2C(C1CC2)(C)C	C25H24FN3O4	449.47	33	16	0.32	4	6	4	127.92	115.42	2.83	3.95	4.09	3.43	4.22	3.7	-5.21	2.77E-03	6.17E-06	Moderately soluble	-6.07	3.79E-04	8.43E-07	Poorly soluble	-7.8	7.06E-06	1.57E-08	Poorly soluble	High	No	Yes	Yes	No	No	No	No	-6.24	0	0	0	0	0	0.55	0	2	2	5.06

7.5	O=C(C1CC2C(C1)OC(O2)(F)F)Nc1ccc(c(c1)C1NC(=N)C2C(C1CC2)(C)C)F	C22H26F3N3O3	437.46	31	6	0.64	4	7	3	112.07	83.44	3.26	3.04	4.55	3.1	3.53	3.49	-4.35	1.97E-02	4.50E-05	Moderately soluble	-4.46	1.52E-02	3.48E-05	Moderately soluble	-5.66	9.64E-04	2.20E-06	Moderately soluble	High	No	Yes	No	No	No	Yes	Yes	-6.81	0	0	0	0	0	0.55	0	1	1	5.38
7.6	N=C1NC(c2cc(ccc2F)NC(=O)C2=NCC(=O)N(c3c2ccc(c3)F)C)C2C(C1CC2)(C)C	C26H27F2N5O2	479.52	35	12	0.38	4	6	3	141.45	97.65	3.06	2.99	3.23	3.16	4.64	3.42	-4.69	9.87E-03	2.06E-05	Moderately soluble	-4.7	9.47E-03	1.97E-05	Moderately soluble	-7.89	6.19E-06	1.29E-08	Poorly soluble	High	No	Yes	No	No	No	No	Yes	-7.1	0	1	0	0	0	0.55	0	1	1	5.7
7.7	N=C1NC(c2cc(ccc2F)NC(=O)c2cc3c([nH]2)cc(cc3)F)C2C(C1CC2)(C)C	C24H24F2N4O	422.47	31	15	0.33	4	4	4	121.67	80.77	3.14	4.33	5.32	4.23	5.19	4.44	-5.28	2.21E-03	5.23E-06	Moderately soluble	-5.74	7.68E-04	1.82E-06	Moderately soluble	-8.36	1.86E-06	4.40E-09	Poorly soluble	High	No	Yes	Yes	No	No	Yes	Yes	-5.8	1	0	0	0	0	0.55	0	1	2	4.82
7.8	O=C(C1C=CC2C(C1O)OC(O2)(F)F)Nc1ccc(c(c1)C1NC(=	C23H26F3	465.47	33	6	0.57	4	8	4	117.57	103.67	2.89	2.86	3.68	2.43	2.47	2.87	-4.4	1.86E-02	4.00E-05	Moderate	-4.7	9.37E-03	2.01E-05	Moderate	-4.63	1.08E-02	2.32E-05	Moderate	High	No	Yes	No	No	No	No	Yes	-7.11	0	0	0	0	0	0.55	0	2	1	6.03

	N)C2C(C1CC2)(C)C)F	N3O4																			ly soluble				ly soluble				ly soluble																			
7.9	N=C1NC(c2cc(ccc2F)NC(=O)c2cnn3c2OCC(C3)(F)F)C2C(C1CC2)(C)C	C22H24F3N5O2	447.45	32	11	0.5	4	7	3	116.23	92.03	2.95	2.76	4.34	3.45	3.27	3.35	-4.34	2.03E-02	4.54E-05	Moderately soluble	-4.35	2.01E-02	4.49E-05	Moderately soluble	-6.3	2.25E-04	5.04E-07	Poorly soluble	High	No	Yes	No	No	No	Yes	Yes	-7.07	0	0	0	0	0	0.55	0	1	1	5.26
7.10	N=C1NC(c2cc(ccc2F)NC(=O)c2ncc3c(c2)sc(c3)C(F)F)C2C(C1CC2)(C)C	C24H23F3N4OS	472.53	33	15	0.38	5	6	3	128.1	106.11	2.92	4.71	6.34	4.34	6.25	4.91	-5.74	8.53E-04	1.81E-06	Moderately soluble	-6.67	1.02E-04	2.15E-07	Poorly soluble	-8.32	2.26E-06	4.78E-09	Poorly soluble	Low	No	Yes	Yes	No	Yes	No	Yes	-5.84	1	1	0	1	0	0.55	0	1	2	5.12
7.11	O=C(c1ccc2c(c1)C=CC(O2)(F)F)Nc1ccc(c(c1)C1NC(=N)C2C(C1CC2)(C)C)F	C25H24F3N3O2	455.47	33	12	0.36	4	6	3	124.46	74.21	3.46	4.84	6.01	4.34	4.96	4.72	-5.72	8.71E-04	1.91E-06	Moderately solub	-6.13	3.36E-04	7.38E-07	Poorly soluble	-7.81	7.07E-06	1.55E-08	Poorly soluble	High	No	Yes	Yes	No	Yes	No	Yes	-5.64	1	1	0	1	0	0.55	0	1	2	5.39

																					le																											
7.12	O=C(C1NC2CC1C1C2OC(O1)(C)C)Nc1ccc(c(c1)C1NC(=N)C2C(C1CC2)(C)C)F	C25H33FN4O3	456.55	33	6	0.68	4	6	4	130.99	95.47	3.22	2.3	2.46	2.71	2.84	2.71	-3.99	4.67E-02	1.02E-04	Soluble	-3.94	5.21E-02	1.14E-04	Soluble	-5.72	8.78E-04	1.92E-06	Moderately soluble	High	No	Yes	No	No	No	Yes	No	-7.45	0	1	0	0	0	0.55	0	1	1	5.99
8.1	Fc1oc2c(c1)cc(nc2)C(=O)Nc1ccc(c(c1)C12CCC(CC1)N=C2N)F	C21H18F2N4O2	396.39	29	15	0.29	4	6	2	107.32	93.51	3.05	2.55	4.18	2.79	4.39	3.39	-4.02	3.76E-02	9.49E-05	Moderately soluble	-4.16	2.74E-02	6.90E-05	Moderately soluble	-7.29	2.05E-05	5.16E-08	Poorly soluble	High	No	Yes	Yes	No	Yes	Yes	Yes	-6.91	0	0	0	0	0	0.55	0	0	1	4.86
8.2	COc1cccc2c1C=C(CO2)C(=O)Nc1ccc(c(c1)C12CCC(CC1)N=C2N)F	C24H24FN3O3	421.46	31	12	0.33	5	5	2	120.38	85.94	3.2	2.47	3.54	3.12	4.49	3.36	-3.97	4.56E-02	1.08E-04	Soluble	-3.92	5.08E-02	1.20E-04	Soluble	-6.92	5.09E-05	1.21E-07	Poorly soluble	High	No	Yes	No	No	Yes	Yes	Yes	-7.12	0	0	0	0	0	0.55	0	0	1	5.31
8.3	Fc1ccc2c(c1)C=C(CO2)C(=O)Nc1ccc(c(c1)C12CCC(CC1)N=C2N)F	C23H21F2N3	409.43	30	12	0.3	4	5	2	113.84	76.71	3.03	2.6	4.09	3.83	4.85	3.68	-4.05	3.66E-02	8.94E-05	Moderately	-3.86	5.65E-02	1.38E-04	Soluble	-7.08	3.41E-05	8.32E-08	Poorly solu	High	No	Yes	Yes	No	Yes	Yes	Yes	-6.95	0	0	0	0	0	0.55	0	0	1	5.17

		O2																			soluble								ble																			
8.4	O=c1ccc2c(o1)cc(c(c2)C(=O)Nc1ccc(c(c1)C12CCC(CC1)N=C2N)F)O	C23H20FN3O4	421.42	31	16	0.26	4	6	3	117.86	117.92	2.7	2.63	3.29	3.01	4.11	3.15	-4.23	2.49E-02	5.92E-05	Moderately soluble	-4.76	7.38E-03	1.75E-05	Moderately soluble	-7.11	3.29E-05	7.82E-08	Poorly soluble	High	No	Yes	Yes	No	Yes	No	No	-7	0	0	0	0	0	0.55	0	1	1	4.92
8.5	O=C(C1CC2C(C1)OC(O2)(F)F)Nc1ccc(c(c1)C12CCC(CC1)N=C2N)F	C20H22F3N3O3	409.4	29	6	0.6	4	7	2	102.01	85.94	2.73	1.72	3.75	2.67	3.43	2.86	-3.35	1.82E-01	4.46E-04	Soluble	-3.14	2.96E-01	7.23E-04	Soluble	-4.96	4.50E-03	1.10E-05	Moderately soluble	High	No	Yes	No	No	No	Yes	Yes	-7.58	0	0	0	0	0	0.55	0	0	1	5.41
8.6	Fc1ccc2c(c1)N(C)C(=O)CN=C2C(=O)Nc1ccc(c(c1)C12CCC(CC1)N=C2N)F	C24H23F2N5O2	451.47	33	12	0.33	4	6	2	131.39	100.15	2.46	1.67	2.43	2.76	4.52	2.77	-3.7	9.09E-02	2.01E-04	Soluble	-3.39	1.85E-01	4.10E-04	Soluble	-7.19	2.89E-05	6.40E-08	Poorly soluble	High	No	Yes	No	No	No	No	Yes	-7.87	0	1	0	0	0	0.55	0	0	1	5.5
8.7	Fc1ccc2c(c1)[nH]c(c2)C(=O)Nc1ccc(c	C22H2	394.	29	15	0.27	4	4	3	111.	83.27	2.26	3.01	4.52	3.81	5.08	3.74	-4.3	1.97E	5.01E	Moder	-4.42	1.49E	3.77E	Moder	-7.66	8.69E	2.20E	Poorl	High	No	Yes	Yes	No	Yes	Yes	Yes	-6.57	0	0	0	0	0	0.55	0	0	1	4.73

	(c1)C12CCC(CC1)N=C2N)F	0F2N4O	42							61									-02	-05	ately soluble		-02	-05	ately soluble		-06	-08	y soluble																			
8.8	O=C(C1C=CC2C(C1O)OC(O2)(F)F)Nc1ccc(c(c1)C12CCC(CC1)N=C2N)F	C21H22F3N3O4	437.41	31	6	0.52	4	8	3	107.51	106.17	2.5	1.53	2.89	2	2.36	2.26	-3.4	1.76E-01	4.03E-04	Soluble	-3.37	1.87E-01	4.28E-04	Soluble	-3.94	5.05E-02	1.15E-04	Soluble	High	No	Yes	No	No	No	Yes	Yes	-7.88	0	0	0	0	0	0.55	0	1	1	5.93
8.9	NC1=NC2CCC1(CC2)c1cc(ccc1F)NC(=O)c1cnn2c1OCC(C2)(F)F	C20H20F3N5O2	419.4	30	11	0.45	4	7	2	106.17	94.53	2.53	1.44	3.54	3.02	3.17	2.74	-3.35	1.85E-01	4.42E-04	Soluble	-3.03	3.91E-01	9.32E-04	Soluble	-5.6	1.05E-03	2.51E-06	Moderately soluble	High	No	Yes	No	No	No	Yes	Yes	-7.84	0	0	0	0	0	0.55	0	0	1	5.05
8.10	NC1=NC2CCC1(CC2)c1cc(ccc1F)NC(=O)c1ncc2c(c1)sc(c2)C(F)F	C22H19F3N4OS	444.47	31	15	0.32	5	6	2	118.04	108.61	2.46	3.38	5.54	3.92	6.14	4.29	-4.75	7.85E-03	1.77E-05	Moderately soluble	-5.34	2.03E-03	4.58E-06	Moderately soluble	-7.63	1.05E-05	2.37E-08	Poorly soluble	Low	No	Yes	Yes	No	Yes	Yes	Yes	-6.61	0	0	0	0	0	0.55	0	0	1	4.94

8.11	O=C(c1ccc2c(c1)C=CC(O2)(F)F)Nc1ccc(c(c1)C12CCC(CC1)N=C2N)F	C23H20F3N3O2	427.42	31	12	0.3	4	6	2	114.41	76.71	2.96	3.52	5.21	3.94	4.85	4.1	-4.73	7.96E-03	1.86E-05	Moderately soluble	-4.81	6.55E-03	1.53E-05	Moderately soluble	-7.11	3.30E-05	7.73E-08	Poorly soluble	High	No	Yes	Yes	No	Yes	Yes	Yes	-6.41	0	0	0	0	0	0.55	0	0	2	5.15
8.12	O=C(C1NC2CC1C1C2OC(O1)(C)C)Nc1ccc(c(c1)C12CCC(CC1)N=C2N)F	C23H29FN4O3	428.5	31	6	0.65	4	6	3	120.93	97.97	2.85	0.98	1.66	2.3	2.73	2.1	-2.99	4.35E-01	1.02E-03	Soluble	-2.63	1.01E+00	2.37E-03	Soluble	-5.02	4.09E-03	9.55E-06	Moderately soluble	High	No	Yes	No	Yes	No	Yes	Yes	-8.22	0	0	0	0	0	0.55	0	0	1	5.98
9.1	NC1=NC2N=CN=C2C(=N1)c1cc(ccc1F)NC(=O)c1ncc2c(c1)cc(o2)F	C19H11F2N7O2	407.33	30	15	0.05	4	9	2	118.86	130.59	2	1.31	1.41	1.39	4.17	2.06	-3.3	2.06E-01	5.05E-04	Soluble	-3.65	9.06E-02	2.22E-04	Soluble	-6.61	9.95E-05	2.44E-07	Poorly soluble	High	No	No	No	No	No	No	No	-7.85	0	0	0	0	0	0.55	0	0	1	4.08
9.2	COc1cccc2c1C=C(CO2)C(=O)Nc1ccc(c(c1)C1=NC(=NC2C1=NC=N2)N)F	C22H17FN6O3	432.41	32	12	0.14	5	8	2	131.91	123.02	2.69	1.23	0.77	2.15	4.25	2.22	-3.24	2.47E-01	5.71E-04	Soluble	-3.41	1.68E-01	3.88E-04	Soluble	-6.24	2.48E-04	5.73E-07	Poorly solub	High	No	Yes	No	No	No	No	Yes	-8.06	0	1	0	0	0	0.55	0	0	1	4.43

																													le																			
9.3	Fc1ccc2c(c1)C=C(CO2)C(=O)Nc1ccc(c(c1)C1=NC(=NC2C1=NC=N2)N)F	C21H14F2N6O2	420.37	31	12	0.1	4	8	2	125.38	113.79	2.46	1.36	1.32	2.41	4.62	2.43	-3.33	1.99E-01	4.73E-04	Soluble	-3.35	1.87E-01	4.45E-04	Soluble	-6.41	1.65E-04	3.94E-07	Poorly soluble	High	No	No	No	No	No	No	Yes	-7.9	0	0	0	0	0	0.55	0	0	1	4.28
9.4	NC1=NC2N=CN=C2C(=N1)c1cc(ccc1F)NC(=O)c1cc2ccc(=O)oc2cc1O	C21H13FN6O4	432.36	32	16	0.05	4	9	3	129.4	155	1.42	1.39	0.52	1.66	3.89	1.78	-3.5	1.36E-01	3.15E-04	Soluble	-4.25	2.44E-02	5.64E-05	Moderately soluble	-6.43	1.60E-04	3.70E-07	Poorly soluble	Low	No	No	No	No	No	No	No	-7.95	0	0	1	1	1	0.55	0	1	1	4.15
9.5	O=C(C1CC2(C1)OC(O2)(F)F)Nc1ccc(c(c1)C1=NC(=NC2C1=NC=N2)N)F	C18H15F3N6O3	420.35	30	6	0.39	4	10	2	113.55	123.02	2.29	0.48	0.98	1.24	1.23	1.64	-2.63	9.80E-01	2.33E-03	Soluble	-2.63	9.79E-01	2.33E-03	Soluble	-4.28	2.19E-02	5.20E-05	Moderately soluble	High	No	No	No	No	No	No	Yes	-8.52	0	0	0	0	0	0.55	0	0	1	4.81
9.6	Fc1ccc2c(c1)N(C)C(=O)CN=C2C(=O)Nc1ccc(c(c1)C1=NC(=NC2C1	C22H16F2N8	462.41	34	12	0.14	4	9	2	142.92	137.23	1.94	0.43	-0.34	1.82	4.29	1.63	-2.98	4.90E-01	1.06E-03	Soluble	-2.88	6.11E-01	1.32E-03	Soluble	-6.52	1.40E-04	3.03E-07	Poorly solu	High	No	No	No	No	No	No	No	-8.82	0	1	0	1	0	0.55	0	0	1	4.58

	=NC=N2)N)F	O2																											ble																			
9.7	Fc1ccc2c(c1)[nH]c(c2)C(=O)Nc1ccc(c(c1)C1=NC(=NC2C1=NC=N2)N)F	C20H13F2N7O	405.36	30	15	0.05	4	7	3	123.15	120.35	1.76	1.77	1.75	2.39	4.86	2.51	-3.57	1.08E-01	2.66E-04	Soluble	-3.92	4.93E-02	1.22E-04	Soluble	-6.98	4.22E-05	1.04E-07	Poorly soluble	High	No	No	Yes	No	No	No	No	-7.52	0	0	0	0	0	0.55	0	0	1	4.04
9.8	O=C(C1C=CC2C(C1O)OC(O2)(F)F)Nc1ccc(c(c1)C1=NC(=NC2C1=NC=N2)N)F	C19H15F3N6O4	448.36	32	6	0.32	4	11	3	119.04	143.25	2.43	0.29	0.12	0.62	2.15	1.12	-2.68	9.43E-01	2.10E-03	Soluble	-2.86	6.18E-01	1.38E-03	Soluble	-3.26	2.45E-01	5.47E-04	Soluble	Low	No	No	No	No	No	No	No	-8.83	0	0	1	1	1	0.55	0	1	1	5.18
9.9	NC1=NC2N=CN=C2C(=N1)c1cc(ccc1F)NC(=O)c1cnn2c1OCC(C2)(F)F	C18H13F3N8O2	430.34	31	11	0.22	4	10	2	117.7	131.61	2.1	0.2	0.78	1.63	2.97	1.54	-2.63	1.00E+00	2.33E-03	Soluble	-2.52	1.29E+00	3.00E-03	Soluble	-4.92	5.11E-03	1.19E-05	Moderately soluble	High	No	No	No	No	No	No	No	-8.78	0	0	0	1	0	0.55	0	0	1	4.19
9.10	NC1=NC2N=CN=C2C(=N1)c1cc(ccc1F)NC(=O)c1ncc2c(c1)sc(c2)C(F)F	C20H12F3N7OS	455.42	32	15	0.1	5	9	2	129.58	145.69	1.97	2.14	2.77	2.5	5.93	3.06	-4.03	4.26E-02	9.36E-05	Moderately solu	-4.83	6.71E-03	1.47E-05	Moderately solu	-6.95	5.12E-05	1.12E-07	Poorly soluble	Low	No	No	Yes	No	No	No	No	-7.56	0	0	1	1	0	0.55	0	0	1	4.13

9.11	NC1=NC2N=CN=C2C(=N1)c1cc(ccc1F)NC(=O)c1ccc2c(c1)C=CC(O2)(F)F	C21H13F3N6O2	438.36	32	12	0.1	4	9	2	125.94	113.79	2.55	2.28	2.44	2.52	4.63	2.88	-4.01	4.31E-02	9.82E-05	ble Moderately soluble	-4.31	2.16E-02	4.93E-05	ble Moderately soluble	-6.44	1.61E-04	3.66E-07	Poorly soluble	High	No	No	No	No	Yes	No	Yes	-7.36	0	0	0	0	0	0.55	0	0	1	4.25
9.12	O=C(C1NC2CC1C1C2OC(O1)(C)C)Nc1ccc(c(c1)C1=NC(=NC2C1=NC=N2)N)F	C21H22FN7O3	439.44	32	6	0.48	4	9	3	132.47	135.05	2.17	-0.26	-1.1	1.32	2.5	0.93	-2.28	2.33E+00	5.30E-03	Soluble	-2.12	3.35E+00	7.63E-03	Soluble	-4.34	1.99E-02	4.53E-05	Moderately soluble	Low	No	No	No	No	No	No	Yes	-9.17	0	2	0	1	0	0.55	0	0	1	5.35
10.1	CC1=NN=C(SC1c1cc(ccc1F)NC(=O)c1ncc2c(c1)cc(o2)F)N	C18H13F2N5O2S	401.39	28	15	0.11	4	7	2	109.73	131.17	1.74	2.78	3.4	2.06	4.09	2.81	-4.21	2.46E-02	6.13E-05	Moderately soluble	-5.19	2.59E-03	6.45E-06	Moderately soluble	-6.72	7.70E-05	1.92E-07	Poorly soluble	High	No	No	Yes	No	Yes	No	No	-6.77	0	0	0	0	0	0.55	0	0	1	4.06
10.2	COc1ccc2c1C=C(CO2)C(=O)Nc1ccc	C21H1	426.	30	12	0.19	5	6	2	122.	123.6	2.67	2.7	2.76	2.42	4.17	2.95	-4.15	3.01E	7.06E	Moder	-4.95	4.80E	1.13E	Moder	-6.35	1.92E	4.50E	Poorl	High	No	Yes	Yes	Yes	Yes	No	Yes	-6.98	0	0	0	0	0	0.55	0	0	1	4.39

	(c(c1)C1SC(=NN=C1C)N)F	9FN4O3S	46							79									-02	-05	ately soluble		-03	-05	ately soluble		-04	-07	y soluble																			
10.3	Fc1ccc2c(c1)C=C(CO2)C(=O)Nc1ccc(c(c1)C1SC(=NN=C1C)N)F	C20H16F2N4O2S	414.43	29	12	0.15	4	6	2	116.25	114.37	2.64	2.83	3.31	3.11	4.53	3.29	-4.23	2.41E-02	5.83E-05	Moderately soluble	-4.89	5.34E-03	1.29E-05	Moderately soluble	-6.51	1.28E-04	3.10E-07	Poorly soluble	High	No	Yes	Yes	Yes	Yes	No	Yes	-6.82	0	0	0	0	0	0.55	0	0	1	4.23
10.4	CC1=NN=C(SC1c1cc(ccc1F)NC(=O)c1cc2ccc(=O)oc2cc1O)N	C20H15FN4O4S	426.42	30	16	0.1	4	7	3	120.27	155.58	1.68	2.86	2.51	2.31	3.81	2.63	-4.42	1.64E-02	3.83E-05	Moderately soluble	-5.79	6.98E-04	1.64E-06	Moderately soluble	-6.54	1.24E-04	2.90E-07	Poorly soluble	Low	No	No	No	No	No	No	No	-6.87	0	0	1	1	1	0.55	0	1	1	4.15
10.5	O=C(C1CC2C(C1)OC(O2)(F)F)Nc1ccc(c(c1)C1SC(=NN=C1C)N)F	C17H17F3N4O3S	414.4	28	6	0.47	4	8	2	104.42	123.6	2.18	1.95	2.97	1.92	3.14	2.43	-3.53	1.22E-01	2.94E-04	Soluble	-4.17	2.80E-02	6.76E-05	Moderately sol	-4.39	1.70E-02	4.09E-05	Moderately sol	High	No	Yes	No	Yes	No	No	Yes	-7.44	0	0	0	0	0	0.55	0	0	1	4.75

10.6	Fc1ccc2c(c1)N(C)C(=O)CN=C2C(=O)Nc1ccc(c(c1)C1SC(=NN=C1C)N)F	C21H18F2N6O2S	456.47	32	12	0.19	4	7	2	133.8	137.81	2.02	1.9	1.65	2.08	4.21	2.37	-3.88	6.01E-02	1.32E-04	Soluble	-4.42	1.75E-02	3.83E-05	Moderately soluble	-6.62	1.09E-04	2.38E-07	Poorly soluble	High	No	Yes	No	No	No	No	Yes	-7.74	0	1	0	1	0	0.55	0	0	1	4.57
10.7	Fc1ccc2c(c1)[nH]c(c2)C(=O)Nc1ccc(c(c1)C1SC(=NN=C1C)N)F	C19H15F2N5OS	399.42	28	15	0.11	4	5	3	114.02	120.93	1.69	3.24	3.74	3.08	4.71	3.31	-4.49	1.29E-02	3.24E-05	Moderately soluble	-5.45	1.41E-03	3.52E-06	Moderately soluble	-7.09	3.27E-05	8.18E-08	Poorly soluble	High	No	Yes	Yes	No	Yes	No	No	-6.44	0	0	0	0	0	0.55	0	0	1	4.01
10.8	O=C(C1C=CC2C(C1O)OC(O2)(F)F)Nc1ccc(c(c1)C1SC(=NN=C1C)N)F	C18H17F3N4O4S	442.41	30	6	0.39	4	9	3	109.92	143.83	1.98	1.76	2.11	1.29	2.07	1.84	-3.58	1.18E-01	2.66E-04	Soluble	-4.4	1.77E-02	4.00E-05	Moderately soluble	-3.37	1.90E-01	4.30E-04	Soluble	Low	No	Yes	No	No	No	No	No	-7.75	0	0	1	1	0	0.55	0	1	1	5.15
10.9	CC1=NN=C(SC1c1ccc(ccc1F)	C17H	424	29	11	0.29	4	8	2	108.	132.	2.14	1.67	2.76	2.29	2.89	2.35	-3.	1.22	2.88	Solu	-4.	3.70	8.71	Mode	-5.	3.96	9.33	Mode	High	No	Yes	No	No	No	No	No	-7.7	0	0	0	1	0	0.55	0	0	1	4.13

	NC(=O)c1cnn2c1OCC(C2)(F)F)N	15F3N6O2S	.4							58	19							54	E-01	E-04	ble	06	E-02	E-05	rately soluble	03	E-03	E-06	rately soluble																		
10.10	CC1=NN=C(SC1c1cc(ccc1F)NC(=O)c1ncc2c(c1)sc(c2)C(F)F)N	C19H14F3N5OS2	449.47	30	15	0.16	5	7	2	120.45	146.27	2.01	3.62	4.76	3.2	5.84	3.89	-4.95	5.07E-03	1.13E-05	Moderately soluble	-6.38	1.88E-04	4.17E-07	Poorly soluble	-7.05	3.96E-05	8.81E-08	Poorly soluble	Low	No	No	Yes	Yes	Yes	No	No	-6.47	0	0	1	1	0	0.55	0	0	2	4.09
10.11	NC1=NN=C(C(S1)c1cc(ccc1F)NC(=O)c1ccc2c(c1)C=CC(O2)(F)F)C	C20H15F3N4O2S	432.42	30	12	0.15	4	7	2	116.82	114.37	2.59	3.75	4.43	3.22	4.54	3.71	-4.92	5.25E-03	1.21E-05	Moderately soluble	-5.84	6.19E-04	1.43E-06	Moderately soluble	-6.54	1.24E-04	2.88E-07	Poorly soluble	High	No	No	Yes	Yes	Yes	No	Yes	-6.28	0	0	0	0	0	0.55	0	0	2	4.23
10.12	O=C(C1NC2CC1C1C2OC(O1)(C)C)Nc1ccc(c(c1)C1SC(=NN=C1C)N)F	C20H24FN5O3S	433.5	30	6	0.55	4	7	3	123.34	135.63	2.3	1.21	0.89	1.59	2.42	1.68	-3.17	2.90E-01	6.70E-04	Soluble	-3.66	9.59E-02	2.21E-04	Soluble	-4.45	1.54E-02	3.56E-05	Moderatelyso	High	No	Yes	No	No	No	Yes	Yes	-8.09	0	0	0	1	0	0.55	0	0	1	5.26

																													luble																			
11.1	NC1=NNC(S1)c1cc(ccc1F)NC(=O)c1ncc2c(c1)cc(o2)F	C16H11F2N5O2S	375.35	26	15	0.06	4	6	3	99.82	130.84	1.64	2.91	2.49	1.93	2.7	2.33	-4.16	2.58E-02	6.86E-05	Moderately soluble	-5.32	1.80E-03	4.80E-06	Moderately soluble	-6.09	3.05E-04	8.13E-07	Poorly soluble	High	No	No	Yes	No	No	No	No	-6.52	0	0	0	0	0	0.55	0	0	1	3.77
11.2	COc1cccc2c1C=C(CO2)C(=O)Nc1ccc(c(c1)C1NN=C(S1)N)F	C19H17FN4O3S	400.43	28	12	0.16	5	5	3	112.88	123.27	2.45	2.83	1.85	2.05	2.77	2.39	-4.09	3.23E-02	8.08E-05	Moderately soluble	-5.08	3.36E-03	8.38E-06	Moderately soluble	-5.72	7.59E-04	1.90E-06	Moderately soluble	High	No	Yes	Yes	No	Yes	Yes	Yes	-6.73	0	0	0	0	0	0.55	0	0	1	4.03
11.3	Fc1ccc2c(c1)C=C(CO2)C(=O)Nc1ccc(c(c1)C1NN=C(S1)N)F	C18H14F2N4O2S	388.39	27	12	0.11	4	5	3	106.35	114.04	2.36	2.96	2.4	3	3.14	2.77	-4.18	2.58E-02	6.64E-05	Moderately soluble	-5.02	3.73E-03	9.60E-06	Moderately soluble	-5.88	5.07E-04	1.31E-06	Moderately soluble	High	No	Yes	Yes	No	Yes	No	Yes	-6.57	0	0	0	0	0	0.55	0	0	1	3.89
11	NC1=NNC(S1)c1cc(cc	C18	400	28	16	0.	4	6	4	110	155	1.	3	1.6	2.2	2.	2.	-4.	1.7	4.2	Mod	-5.	4.7	1.1	Mod	-5.	4.9	1.2	Mod	Low	No	No	No	No	No	No	No	-6.	0	0	1	1	1	0.	0	1	1	3.

.4	c1F)NC(=O)c1cc2ccc(=O)oc2cc1O	H13FN4O4S	.38			06				.36	.25	61				41	16	37	0E-02	5E-05	erately soluble	92	6E-04	9E-06	erately soluble	91	0E-04	2E-06	erately soluble									61						55				79
11.5	O=C(C1CC2C(C1)OC(O2)(F)F)Nc1ccc(c(c1)C1NN=C(S1)N)F	C15H15F3N4O3S	388.36	26	6	0.47	4	7	3	94.51	123.27	2.22	2.09	2.06	1.8	1.76	1.99	-3.47	1.31E-01	3.38E-04	Soluble	-4.31	1.91E-02	4.91E-05	Moderately soluble	-3.76	6.70E-02	1.73E-04	Soluble	High	No	Yes	No	Yes	No	No	No	-7.19	0	0	0	0	0	0.55	0	0	1	4.32
11.6	Fc1ccc2c(c1)N(C)C(=O)CN=C2C(=O)Nc1ccc(c(c1)C1NN=C(S1)N)F	C19H16F2N6O2S	430.43	30	12	0.16	4	6	3	123.89	137.48	1.63	2.03	0.74	1.71	2.81	1.78	-3.82	6.52E-02	1.52E-04	Soluble	-4.54	1.23E-02	2.85E-05	Moderately soluble	-6	4.29E-04	9.98E-07	Poorly soluble	High	No	Yes	No	No	No	No	Yes	-7.48	0	0	0	1	0	0.55	0	0	1	4.25
11.7	Fc1ccc2c(c1)[nH]c(c2)C(=O)Nc1ccc(c(c1)C1NN=C(S1)N)F	C17H13F2N5	373.38	26	15	0.06	4	4	4	104.11	120.6	1.55	3.37	2.83	2.96	3.38	2.82	-4.44	1.35E-02	3.62E-05	Moderately s	-5.58	9.80E-04	2.63E-06	Moderately s	-6.46	1.29E-04	3.47E-07	Poorly solub	High	No	Yes	Yes	No	Yes	No	No	-6.18	0	0	0	0	0	0.55	0	0	1	3.64

		OS																			oluble				oluble				le																			
11.8	O=C(C1C=CC2C(C1O)OC(O2)(F)F)Nc1ccc(c(c1)C1NN=C(S1)N)F	$C_{16}H_{15}F_3N_4O_4S$	416.37	28	6	0.38	4	8	4	100.01	143.5	1.95	1.9	1.19	1.17	0.69	1.38	-3.51	1.28E-01	3.07E-04	Soluble	-4.54	1.21E-02	2.91E-05	Moderately soluble	-2.74	7.51E-01	1.80E-03	Soluble	Low	No	Yes	No	No	No	No	No	-7.49	0	0	1	1	0	0.55	0	1	1	4.8
11.9	NC1=NNC(S1)c1cc(cc1F)NC(=O)c1cnn2c1OCC(C2)(F)F	$C_{15}H_{13}F_3N_6O_2S$	398.36	27	11	0.27	4	7	3	98.67	131.86	1.89	1.81	1.85	2.16	1.51	1.84	-3.49	1.30E-01	3.25E-04	Soluble	-4.2	2.52E-02	6.33E-05	Moderately soluble	-4.41	1.57E-02	3.94E-05	Moderately soluble	High	No	Yes	No	No	No	No	No	-7.44	0	0	0	1	0	0.55	0	0	1	3.81
11.10	NC1=NNC(S1)c1cc(cc1F)NC(=O)c1ncc2c(c1)sc(c2)C(F)F	$C_{17}H_{12}F_3N_5OS_2$	423.44	28	15	0.12	5	6	3	110.55	145.94	2.33	3.75	3.85	3.08	4.45	3.49	-4.89	5.40E-03	1.28E-05	Moderately soluble	-6.51	1.32E-04	3.11E-07	Poorly soluble	-6.43	1.57E-04	3.70E-07	Poorly soluble	Low	No	No	Yes	No	Yes	No	Yes	-6.22	0	0	1	1	0	0.55	0	0	2	3.78

11.11	NC1=NNC(S1)c1cc(ccc1F)NC(=O)c1ccc2c(c1)C=CC(O2)(F)F	C18H13F3N4O2S	406.38	28	12	0.11	4	6	3	106.91	114.04	2.35	3.89	3.52	3.12	3.15	3.2	-4.86	5.57E-03	1.37E-05	Moderately soluble	-5.98	4.23E-04	1.04E-06	Moderately soluble	-5.92	4.92E-04	1.21E-06	Moderately soluble	High	No	No	Yes	No	Yes	No	Yes	-6.02	0	0	0	0	0	0.55	0	0	2	3.91
11.12	O=C(C1NC2CC1C1C2OC(O1)(C)C)Nc1ccc(c(c1)C1NN=C(S1)N)F	C18H22FN5O3S	407.46	28	6	0.56	4	6	4	113.44	135.3	2.21	1.34	-0.03	1.49	1.02	1.21	-3.11	3.20E-01	7.85E-04	Soluble	-3.78	6.72E-02	1.65E-04	Soluble	-3.83	6.10E-02	1.50E-04	Soluble	High	No	Yes	No	Yes	No	Yes	No	-7.83	0	0	0	1	0	0.55	0	0	1	4.87
12.1	NC1=NC(CS1)Cc1cc(ccc1F)NC(=O)c1ncc2c(c1)cc(o2)F	C18H14F2N4O2S	388.39	27	15	0.17	5	6	2	102.72	118.81	2.18	2.79	3.6	2.12	4.02	2.94	-4.09	3.18E-02	8.19E-05	Moderately soluble	-4.94	4.45E-03	1.14E-05	Moderately soluble	-6.71	7.54E-05	1.94E-07	Poorly soluble	High	No	Yes	Yes	Yes	Yes	Yes	Yes	-6.69	0	0	0	0	0	0.55	0	0	1	3.75
12.2	COc1cccc2c1C=C(CO2)C(=O)Nc1ccc(c(c1)CC1CSC(=N1)N)F	C21H20FN3O3S	413.47	29	12	0.24	6	5	2	115.78	111.24	3.29	2.72	2.96	2.48	4.11	3.11	-4.03	3.88E-02	9.39E-05	Moderately sol	-4.71	8.07E-03	1.95E-05	Moderately sol	-6.34	1.88E-04	4.54E-07	Poorly soluble	High	No	Yes	Yes	Yes	Yes	Yes	Yes	-6.89	0	0	0	0	0	0.55	0	0	1	4.16

																					uble				uble																							
12.3	Fc1ccc2c(c1)C=C(CO2)C(=O)Nc1ccc(c(c1)CC1CSC(=N1)N)F	C20H17F2N3O2S	401.43	28	12	0.2	5	5	2	109.24	102.01	2.96	2.85	3.51	3.19	4.47	3.4	-4.11	3.11E-02	7.74E-05	Moderatelysoluble	-4.65	8.97E-03	2.23E-05	Moderatelysoluble	-6.5	1.25E-04	3.13E-07	Poorlysoluble	High	No	Yes	Yes	Yes	Yes	Yes	Yes	-6.73	0	0	0	0	0	0.55	0	0	1	4.02
12.4	NC1=NC(CS1)Cc1cc(ccc1F)NC(=O)c1cc2ccc(=O)oc2cc1O	C20H16FN3O4S	413.42	29	16	0.15	5	6	3	113.26	143.22	2.09	2.88	2.71	2.36	3.74	2.76	-4.3	2.09E-02	5.06E-05	Moderatelysoluble	-5.55	1.17E-03	2.84E-06	Moderatelysoluble	-6.53	1.21E-04	2.93E-07	Poorlysoluble	Low	No	No	Yes	No	Yes	No	Yes	-6.78	0	0	1	1	0	0.55	0	1	1	3.8
12.5	O=C(C1CC2C(C1)OC(O2)(F)F)Nc1ccc(c(c1)CC1CSC(=N1)N)F	C17H18F3N3O3S	401.4	27	6	0.53	5	7	2	97.41	111.24	2.7	1.97	3.17	1.99	3.07	2.58	-3.4	1.58E-01	3.94E-04	Soluble	-3.93	4.70E-02	1.17E-04	Soluble	-4.38	1.66E-02	4.13E-05	Moderatelysoluble	High	No	Yes	No	Yes	No	Yes	Yes	-7.35	0	0	0	0	0	0.55	0	0	1	4.4
12.6	Fc1ccc2c(c1)N(C)C(=O)CN=C	C21H	443.	31	12	0.24	5	6	2	126.	125.	2.35	1.92	1.85	2.13	4.15	2.48	-3.	7.79	1.76	Solu	-4.	2.94	6.64	Mode	-6.	1.06	2.40	Poor	High	No	Yes	No	Yes	Yes	No	Yes	-7.	0	0	0	0	0	0.55	0	0	1	4.36

	2C(=O)Nc1ccc(c(c1)CC1CSC(=N1)N)F	19F2N5O2S	47							79	45							76	E-02	E-04	ble	18	E-02	E-05	rately soluble	62	E-04	E-07	ly soluble									64										
12.7	Fc1ccc2c(c1)[nH]c(c2)C(=O)Nc1ccc(c(c1)CC1CSC(=N1)N)F	C19H16F2N4OS	386.42	27	15	0.16	5	4	3	107.01	108.57	2.06	3.25	3.94	3.16	4.71	3.42	-4.36	1.67E-02	4.32E-05	Moderately soluble	-5.2	2.42E-03	6.26E-06	Moderately soluble	-7.08	3.20E-05	8.28E-08	Poorly soluble	High	No	Yes	Yes	Yes	Yes	Yes	Yes	-6.35	0	0	0	0	0	0.55	0	0	1	3.63
12.8	O=C(C1C=CC2C(C1O)OC(O2)(F)F)Nc1ccc(c(c1)CC1CSC(=N1)N)F	C18H18F3N3O4S	429.41	29	6	0.44	5	8	3	102.91	131.47	2.42	1.78	2.31	1.34	2	1.97	-3.45	1.53E-01	3.57E-04	Soluble	-4.16	2.98E-02	6.93E-05	Moderately soluble	-3.36	1.86E-01	4.33E-04	Soluble	High	No	Yes	No	No	No	No	Yes	-7.66	0	0	0	0	0	0.55	0	1	1	4.91
12.9	NC1=NC(CS1)Cc1cc(ccc1F)NC(=O)c1cnn2c1OCC(C2)(F)F	C17H16F3N5O	411.4	28	11	0.35	5	7	2	101.57	119.83	2.49	1.69	2.96	2.34	2.82	2.46	-3.42	1.58E-01	3.84E-04	Soluble	-3.82	6.21E-02	1.51E-04	Soluble	-5.03	3.87E-03	9.42E-06	Moderately so	High	No	Yes	No	Yes	No	No	Yes	-7.61	0	0	0	0	0	0.55	0	0	1	3.91

		2S																											luble																			
12.10	NC1=NC(CS1)Cc1cc(ccc1F)NC(=O)c1ncc2c(c1)sc(c2)C(F)F	C19H15F3N4OS2	436.47	29	15	0.21	6	6	2	113.44	133.91	2.25	3.63	4.96	3.27	5.78	3.98	-4.82	6.61E-03	1.51E-05	Moderately soluble	-6.13	3.23E-04	7.41E-07	Poorly soluble	-7.05	3.87E-05	8.87E-08	Poorly soluble	Low	No	Yes	Yes	Yes	Yes	Yes	Yes	-6.39	0	0	0	1	0	0.55	0	0	2	3.81
12.11	NC1=NC(CS1)Cc1cc(ccc1F)NC(=O)c1ccc2c(c1)C=CC(O2)(F)F	C20H16F3N3O2S	419.42	29	12	0.2	5	6	2	109.81	102.01	2.93	3.77	4.63	3.3	4.48	3.82	-4.79	6.78E-03	1.62E-05	Moderately soluble	-5.61	1.04E-03	2.48E-06	Moderately soluble	-6.54	1.22E-04	2.90E-07	Poorly soluble	High	No	Yes	Yes	Yes	Yes	No	Yes	-6.18	0	0	0	0	0	0.55	0	0	2	3.98
12.12	O=C(C1NC2CC1C12OC(O1)(C)C)Nc1ccc(c(c1)CC1CSC(=N1)N)F	C20H25FN4O3S	420.5	29	6	0.6	5	6	3	116.33	123.27	2.89	1.22	1.09	1.65	2.36	1.84	-3.04	3.85E-01	9.15E-04	Soluble	-3.41	1.65E-01	3.93E-04	Soluble	-4.45	1.51E-02	3.59E-05	Moderately soluble	High	No	Yes	No	Yes	No	Yes	Yes	-8	0	0	0	0	0	0.55	0	0	1	4.97
13	NC1=NC2C(S1)CCCC	C21	428	30	15	0.	4	6	2	115	118	2.	3.5	4.	2.	4.	3.	-4.	6.6	1.5	Mod	-5.	8.9	2.1	Mod	-6.	5.1	1.2	Poo	Low	No	Yes	No	Yes	Yes	Yes	Yes	-6.	0	0	0	0	0	0.	0	0	1	4.

.1	2c1cc(ccc1F)NC(=O)c1ncc2c(c1)cc(o2)F	H18F2N4O2S	.46			29				.03	.81	61		69	79	15	55	81	8E-03	6E-05	erately soluble	68	9E-04	0E-06	erately soluble	92	6E-05	1E-07	rly soluble									43						55				45
13.2	COc1cccc2c1C=C(CO2)C(=O)Nc1ccc(c(c1)C1CCCC2C1N=C(S2)N)F	C24H24FN3O3S	453.53	32	12	0.33	5	5	2	128.08	111.24	3.43	3.42	4.05	3.12	4.24	3.65	-4.75	7.99E-03	1.76E-05	Moderately soluble	-5.44	1.66E-03	3.66E-06	Moderately soluble	-6.55	1.29E-04	2.84E-07	Poorly soluble	High	No	Yes	No	Yes	Yes	Yes	Yes	-6.64	0	0	0	0	0	0.55	0	0	1	4.88
13.3	Fc1ccc2c(c1)C=C(CO2)C(=O)Nc1ccc(c(c1)C1CCCC2C1N=C(S2)N)F	C23H21F2N3O2S	441.49	31	12	0.3	4	5	2	121.55	102.01	3.3	3.55	4.61	3.83	4.6	3.98	-4.84	6.44E-03	1.46E-05	Moderately soluble	-5.38	1.85E-03	4.20E-06	Moderately soluble	-6.71	8.59E-05	1.95E-07	Poorly soluble	High	No	Yes	Yes	Yes	Yes	Yes	Yes	-6.47	0	0	0	0	0	0.55	0	0	2	4.73
13.4	NC1=NC2C(S1)CCCC2c1cc(ccc1F)NC(=O)c1cc2ccc(=O)oc2cc1O	C23H20FN3O	453.49	32	16	0.26	4	6	3	125.57	143.22	2.11	3.58	3.8	3.01	3.87	3.28	-5.01	4.40E-03	9.70E-06	Moderatelys	-6.27	2.41E-04	5.32E-07	Poorly solub	-6.74	8.31E-05	1.83E-07	Poorly solub	Low	No	Yes	No	No	Yes	No	No	-6.52	0	0	1	1	0	0.55	0	1	2	4.53

		4S																			oluble				le				le																			
13.5	O=C(C1CC2C(C1)OC(O2)(F)F)Nc1ccc(c(c1)C1CCCC2C1N=C(S2)N)F	C20H22F3N3O3S	441.47	30	6	0.6	4	7	2	109.72	111.24	3	2.67	4.26	2.67	3.19	3.16	-4.14	3.17E-02	7.19E-05	Moderately soluble	-4.66	9.71E-03	2.20E-05	Moderately soluble	-4.59	1.13E-02	2.57E-05	Moderately soluble	High	No	Yes	No	Yes	No	Yes	Yes	-7.1	0	0	0	0	0	0.55	0	0	1	4.91
13.6	Fc1ccc2c(c1)N(C)C(=O)CN=C2C(=O)Nc1ccc(c(c1)C1CCCC2C1N=C(S2)N)F	C24H23F2N5O2S	483.53	34	12	0.33	4	6	2	139.09	125.45	2.73	2.62	2.94	2.76	4.28	3.06	-4.49	1.58E-02	3.27E-05	Moderately soluble	-4.9	6.03E-03	1.25E-05	Moderately soluble	-6.82	7.28E-05	1.51E-07	Poorly soluble	High	No	Yes	No	Yes	Yes	No	Yes	-7.39	0	2	0	0	0	0.55	0	0	1	5.06
13.7	Fc1ccc2c(c1)[nH]c(c2)C(=O)Nc1ccc(c(c1)C1CCCC2C1N=C(S2)N)F	C22H20F2N4OS	426.48	30	15	0.27	4	4	3	119.32	108.57	2.48	3.96	5.03	3.81	4.84	4.03	-5.08	3.51E-03	8.22E-06	Moderately soluble	-5.94	4.89E-04	1.15E-06	Moderately soluble	-7.29	2.19E-05	5.13E-08	Poorly soluble	High	No	Yes	Yes	Yes	Yes	Yes	Yes	-6.09	0	0	0	0	0	0.55	0	0	2	4.35

13.8	O=C(C1C=CC2C(C1O)OC(O2)(F)F)Nc1ccc(c(c1)C1CCCC2C1N=C(S2)N)F	C21H22F3N3O4S	469.48	32	6	0.52	4	8	3	115.21	131.47	2.78	2.48	3.4	2	2.12	2.56	-4.19	3.05E-02	6.49E-05	Moderately soluble	-4.89	6.11E-03	1.30E-05	Moderately soluble	-3.57	1.27E-01	2.71E-04	Soluble	High	No	Yes	No	Yes	No	Yes	Yes	-7.4	0	0	0	0	0	0.55	0	1	1	5.37
13.9	NC1=NC2C(S1)CCCC2c1cc(ccc1F)NC(=O)c1cnn2c1OCC(C2)(F)F	C20H20F3N5O2S	451.47	31	11	0.45	4	7	2	113.88	119.83	2.97	2.39	4.06	3.02	2.93	3.07	-4.14	3.25E-02	7.19E-05	Moderately soluble	-4.55	1.28E-02	2.83E-05	Moderately soluble	-5.23	2.65E-03	5.87E-06	Moderately soluble	High	No	Yes	No	Yes	No	Yes	Yes	-7.36	0	0	0	0	0	0.55	0	0	1	4.63
13.10	NC1=NC2C(S1)CCCC2c1cc(ccc1F)NC(=O)c1ncc2c(c1)sc(c2)C(F)F	C22H19F3N4OS2	476.54	32	15	0.32	5	6	2	125.75	133.91	2.66	4.33	6.06	3.92	5.9	4.58	-5.54	1.38E-03	2.89E-06	Moderately soluble	-6.86	6.63E-05	1.39E-07	Poorly soluble	-7.25	2.66E-05	5.57E-08	Poorly soluble	Low	No	Yes	No	Yes	Yes	No	Yes	-6.13	0	1	0	2	0	0.55	0	0	2	4.52
13.11	NC1=NC2C(S1)CCCC2c1cc(ccc1F)NC(=O)c1ccc2c(c1)C=C	C23H20F3	459.48	32	12	0.3	4	6	2	122.11	102.01	3.24	4.47	5.72	3.94	4.61	4.4	-5.52	1.39E-03	3.03E-06	Moderate	-6.33	2.14E-04	4.66E-07	Poorlyso	-6.74	8.32E-05	1.81E-07	Poorlyso	Low	No	Yes	Yes	Yes	Yes	No	Yes	-5.93	0	1	0	0	0	0.55	0	0	2	4.7

	C(O2)(F)F	N3O2S																			lysoluble				luble				luble																			
13.12	O=C(C1NC2CC1C1C2OC(O1)(C)C)Nc1ccc(c(c1)C1CCCC2C1N=C(S2)N)F	C23H29FN4O3S	460.56	32	6	0.65	4	6	3	128.64	123.27	3.36	1.93	2.18	2.3	2.49	2.45	-3.79	7.54E-02	1.64E-04	Soluble	-4.14	3.32E-02	7.20E-05	Moderately soluble	-4.65	1.03E-02	2.24E-05	Moderately soluble	High	No	Yes	No	Yes	No	Yes	No	-7.74	0	0	0	0	0	0.55	0	0	1	5.43
14.1	NC1=NC(CC2(S1)CCCCC2)c1cc(ccc1F)NC(=O)c1ncc2c(c1)cc(o2)F	C23H22F2N4O2S	456.51	32	15	0.35	4	6	2	124.68	118.81	2.95	4.48	5.5	3.21	5	4.23	-5.58	1.21E-03	2.66E-06	Moderately soluble	-6.7	9.21E-05	2.02E-07	Poorly soluble	-7.9	5.76E-06	1.26E-08	Poorly soluble	Low	No	Yes	Yes	Yes	Yes	No	Yes	-5.9	0	0	0	0	0	0.55	0	0	2	4.75
14.2	COc1ccc2c1C=C(CO2)C(=O)Nc1ccc(c(c1)C1N=C(N)SC2(C1)CCCCC2)F	C26H28FN3O3S	481.58	34	12	0.38	5	5	2	137.74	111.24	3.82	4.41	4.86	3.53	5.1	4.35	-5.54	1.40E-03	2.92E-06	Moderatelysolub	-6.46	1.66E-04	3.44E-07	Poorly soluble	-7.53	1.44E-05	2.99E-08	Poorly soluble	High	No	Yes	No	No	Yes	No	Yes	-6.11	0	2	0	0	0	0.55	0	0	2	5.16

																					le																																								
14.3	Fc1ccc2c(c1)C=C(CO2)C(=O)Nc1ccc(c(c1)C1N=C(N)SC2(C1)CCCCC2)F	C25H25F2N3O2S	469.55	33	12	0.36	4	5	2	131.2	102.01	3.75	4.54	5.41	4.24	5.46	4.68	-5.62	1.14E-03	2.42E-06	Moderately soluble	-6.4	1.85E-04	3.94E-07	Poorly soluble	-7.69	9.59E-06	2.04E-08	Poorly soluble	High	No	Yes	Yes	No	Yes	No	Yes	-5.94	1	1	0	0	0	0.55	0	0	2	5													
14.4	NC1=NC(CC2(S1)CCCCC2)c1cc(ccc1F)NC(=O)c1cc2ccc(=O)oc2cc1O	C25H24FN3O4S	481.54	34	16	0.32	4	6	3	135.22	143.22	2.68	4.57	4.61	3.43	4.73	4	-5.79	7.83E-04	1.63E-06	Moderately soluble	-7.3	2.41E-05	5.00E-08	Poorly soluble	-7.71	9.28E-06	1.93E-08	Poorly soluble	Low	No	Yes	Yes	No	Yes	No	Yes	-5.99	0	2	1	1	0	0.55	0	1	2	4.85													
14.5	O=C(C1CC2C(C1)OC(O2)(F)F)Nc1ccc(c(c1)C1N=C(N)SC2(C1)CCCCC2)F	C22H26F3N3O3S	469.52	32	6	0.64	4	7	2	119.37	111.24	3.41	3.66	5.07	3.1	4.04	3.86	-4.93	5.50E-03	1.17E-05	Moderately soluble	-5.69	9.69E-04	2.06E-06	Moderately soluble	-5.57	1.27E-03	2.70E-06	Moderately soluble	High	No	Yes	No	Yes	No	No	Yes	-6.57	0	0	0	0	0	0.55	0	0	2	5.41													
14.6	Fc1ccc2c(c1)N(C)C(=O)CN=C2C(=O)Nc1ccc(	C26H27	511.59	36	12	0.38	4	6	2	148.75	125.45	3.25	3.61	3.75	3.16	5.13	3.78	-5.27	2.75E-	5.39E-	Modera	-5.93	5.99E-	1.17E-	Modera	-7.8	8.15E-	1.59E-	Poorly	High	No	Yes	No	No	Yes	No	Yes	-6.86	1	2	0	0	0	0.55	0	0	2	5.34													

	c(c1)C1N=C(N)SC2(C1)CCCCC2)F	F2N5O2S																	03	06	tely soluble		04	06	tely soluble		06	08	soluble																			
14.7	Fc1ccc2c(c1)[nH]c(c2)C(=O)Nc1ccc(c(c1)C1N=C(N)SC2(C1)CCCCC2)F	C24H24F2N4OS	454.54	32	15	0.33	4	4	3	128.97	108.57	2.86	4.94	5.84	4.23	5.7	4.71	-5.85	6.37E-04	1.40E-06	Moderately soluble	-6.96	5.01E-05	1.10E-07	Poorly soluble	-8.27	2.44E-06	5.37E-09	Poorly soluble	Low	No	Yes	Yes	No	Yes	No	Yes	-5.57	1	1	0	0	0	0.55	0	0	2	4.67
14.8	O=C(C1C=CC2C(C1O)OC(O2)(F)F)Nc1ccc(c(c1)C1N=C(N)SC2(C1)CCCCC2)F	C23H26F3N3O4S	497.53	34	6	0.57	4	8	3	124.87	131.47	3.26	3.47	4.21	2.43	2.97	3.27	-4.98	5.24E-03	1.05E-05	Moderately soluble	-5.91	6.08E-04	1.22E-06	Moderately soluble	-4.54	1.42E-02	2.86E-05	Moderately soluble	Low	No	Yes	No	Yes	No	No	Yes	-6.87	0	1	0	0	0	0.55	0	1	1	5.88
14.9	NC1=NC(CC2(S1)CCCCC2)c1cc(ccc1F)NC(=O)c1cnn2c1OCC(C2)(F)F	C22H24F3N5O2S	479.52	33	11	0.5	4	7	2	123.53	119.83	3.24	3.38	4.86	3.45	3.78	3.74	-4.93	5.70E-03	1.19E-05	Moderately solu	-5.58	1.28E-03	2.66E-06	Moderately solu	-6.21	2.96E-04	6.18E-07	Poorly soluble	Low	No	Yes	Yes	No	No	Yes	Yes	-6.83	0	0	0	0	0	0.55	0	0	1	4.91

																					ble				ble																							
14.10	NC1=NC(CC2(S1)CCCCC2)c1cc(ccc1F)NC(=O)c1ncc2c(c1)sc(c2)C(F)F	$C_{24}H_{23}F_3N_4OS_2$	504.59	34	15	0.38	5	6	2	135.4	133.91	3.09	5.32	6.86	4.34	6.76	5.27	-6.32	2.43E-04	4.82E-07	Poorly soluble	-7.88	6.59E-06	1.31E-08	Poorly soluble	-8.23	2.97E-06	5.89E-09	Poorly soluble	Low	No	Yes	Yes	No	Yes	No	Yes	-5.6	2	3	0	2	1	0.17	0	0	2	4.81
14.11	NC1=NC(CC2(S1)CCCCC2)c1cc(ccc1F)NC(=O)c1ccc2c(c1)C=CC(O2)(F)F	$C_{25}H_{24}F_3N_3O_2S$	487.54	34	12	0.36	4	6	2	131.76	102.01	3.64	5.46	6.53	4.34	5.46	5.09	-6.3	2.45E-04	5.02E-07	Poorly soluble	-7.36	2.13E-05	4.37E-08	Poorly soluble	-7.72	9.31E-06	1.91E-08	Poorly soluble	Low	No	Yes	Yes	No	Yes	No	Yes	-5.4	1	3	0	1	1	0.55	0	0	2	4.99
14.12	O=C(C1NC2CC1C1C2OC(O1)(C)C)Nc1ccc(c(c1)C1N=C(N)SC2(C1)CCCC2)F	$C_{25}H_{33}FN_4O_3S$	488.62	34	6	0.68	4	6	3	138.29	123.27	3.45	2.92	2.98	2.71	3.34	3.08	-4.58	1.30E-02	2.66E-05	Moderately soluble	-5.17	3.30E-03	6.76E-06	Moderately soluble	-5.63	1.15E-03	2.36E-06	Moderately soluble	High	No	Yes	No	Yes	No	Yes	Yes	-7.21	0	2	0	0	0	0.55	0	0	1	6.01
15.1	NC1=NC(CC2(S1)CCCC2)c1cc(ccc1F)NC(=O)c1ncc2c(c1)cc(o2)F	$C_{22}H_{20}F_2N_4O_2S$	442.48	31	15	0.32	4	6	2	119.87	118.81	2.78	3.94	5.11	3	4.77	3.92	-5.16	3.06E-03	6.92E-06	Moderately sol	-6.13	3.24E-04	7.33E-07	Poorly soluble	-7.63	1.03E-05	2.32E-08	Poorly soluble	Low	No	Yes	Yes	Yes	Yes	Yes	Yes	-6.2	0	0	0	0	0	0.55	0	0	2	4.64

																				uble																												
15.2	COc1cccc2c1C=C(CO2)C(=O)Nc1ccc(c(c1)C1N=C(N)SC2(C1)CCCC2)F	C25H26FN3O3S	467.56	33	12	0.36	5	5	2	132.93	111.24	3.76	3.86	4.47	3.33	4.87	4.06	-5.11	3.63E-03	7.77E-06	Moderately soluble	-5.89	5.99E-04	1.28E-06	Moderately soluble	-7.26	2.56E-05	5.48E-08	Poorly soluble	High	No	Yes	Yes	Yes	Yes	Yes	Yes	-6.41	0	1	0	0	0	0.55	0	0	2	5.04
15.3	Fc1ccc2c(c1)C=C(CO2)C(=O)Nc1ccc(c(c1)C1N=C(N)SC2(C1)CCCC2)F	C24H23F2N3O2S	455.52	32	12	0.33	4	5	2	126.4	102.01	3.6	3.99	5.02	4.04	5.23	4.37	-5.19	2.93E-03	6.44E-06	Moderately soluble	-5.83	6.68E-04	1.47E-06	Moderately soluble	-7.42	1.71E-05	3.76E-08	Poorly soluble	High	No	Yes	Yes	No	Yes	No	Yes	-6.25	0	0	0	0	0	0.55	0	0	2	4.89
15.4	NC1=NC(CC2(S1)CCCC2)c1cc(ccc1F)NC(=O)c1cc2ccc(=O)oc2cc1O	C24H22FN3O4S	467.51	33	16	0.29	4	6	3	130.41	143.22	2.53	4.03	4.22	3.22	4.5	3.7	-5.37	1.98E-03	4.24E-06	Moderately soluble	-6.74	8.49E-05	1.82E-07	Poorly soluble	-7.45	1.65E-05	3.54E-08	Poorly soluble	Low	No	Yes	Yes	No	Yes	No	Yes	-6.29	0	1	1	1	0	0.55	0	1	2	4.73
15.5	O=C(C1CC2C(C1)OC(O2)(F)F	C21H	451.	31	6	0.62	4	7	2	114.	111.	3.29	3.12	4.68	2.89	3.81	3.56	-4.	1.41	3.10	Mode	-5.	3.42	7.50	Mode	-5.3	2.26	4.97	Mode	High	No	Yes	No	Yes	No	Yes	Yes	-6.	0	0	0	0	0	0.55	0	0	1	5.3

	)Nc1ccc(c(c1)C1N=C(N)SC2(C1)CCCC2)F	24F3N3O3S	49							56	24							51	E-02	E-05	rately soluble	12	E-03	E-06	rately soluble		E-03	E-06	rately soluble									86										
15.6	Fc1ccc2c(c1)N(C)C(=O)CN=C2C(=O)Nc1ccc(c(c1)C1N=C(N)SC2(C1)CCCC2)F	C25H25F2N5O2S	497.56	35	12	0.36	4	6	2	143.94	125.45	2.93	3.06	3.36	2.96	4.9	3.44	-4.84	7.15E-03	1.44E-05	Moderately soluble	-5.36	2.17E-03	4.35E-06	Moderately soluble	-7.54	1.45E-05	2.92E-08	Poorly soluble	High	No	Yes	No	No	Yes	No	Yes	-7.16	0	2	0	0	0	0.55	0	0	1	5.22
15.7	Fc1ccc2c(c1)[nH]c(c2)C(=O)Nc1ccc(c(c1)C1N=C(N)SC2(C1)CCCC2)F	C23H22F2N4OS	440.51	31	15	0.3	4	4	3	124.16	108.57	2.71	4.4	5.45	4.03	5.47	4.41	-5.44	1.61E-03	3.65E-06	Moderately soluble	-6.4	1.77E-04	4.01E-07	Poorly soluble	-8.01	4.35E-06	9.89E-09	Poorly soluble	Low	No	Yes	Yes	No	Yes	No	Yes	-5.86	0	0	0	0	0	0.55	0	0	2	4.56
15.8	O=C(C1C=CC2C(C1O)OC(O2)(F)F)Nc1ccc(c(c1)C1N=C(N)SC2(C1)CCC2)F	C22H24F3N3O	483.5	33	6	0.55	4	8	3	120.06	131.47	3.33	2.93	3.82	2.22	2.74	3.01	-4.55	1.35E-02	2.79E-05	Moderately so	-5.35	2.15E-03	4.44E-06	Moderately so	-4.28	2.54E-02	5.25E-05	Moderately so	Low	No	Yes	No	Yes	No	Yes	Yes	-7.17	0	1	0	0	0	0.55	0	1	1	5.77

		4S																			luble				luble				luble																			
15.9	NC1=NC(CC2(S1)CCCC2)c1cc(ccc1F)NC(=O)c1cnn2c1OCC(C2)(F)F	C21H22F3N5O2S	465.49	32	11	0.48	4	7	2	118.72	119.83	3.13	2.84	4.47	3.23	3.55	3.45	-4.51	1.45E-02	3.12E-05	Moderately soluble	-5.01	4.50E-03	9.67E-06	Moderately soluble	-5.95	5.28E-04	1.14E-06	Moderately soluble	High	No	Yes	Yes	No	No	Yes	Yes	-7.12	0	0	0	0	0	0.55	0	0	1	4.79
15.10	NC1=NC(CC2(S1)CCCC2)c1cc(ccc1F)NC(=O)c1ncc2c(c1)sc(c2)C(F)F	C23H21F3N4OS2	490.56	33	15	0.35	5	6	2	130.59	133.91	2.95	4.78	6.47	4.13	6.53	4.97	-5.9	6.19E-04	1.26E-06	Moderately soluble	-7.32	2.33E-05	4.75E-08	Poorly soluble	-7.97	5.29E-06	1.08E-08	Poorly soluble	Low	No	Yes	Yes	No	Yes	No	Yes	-5.9	0	3	0	2	0	0.55	0	0	2	4.7
15.11	NC1=NC(CC2(S1)CCCC2)c1cc(ccc1F)NC(=O)c1ccc2c(c1)C=CC(O2)(F)F	C24H22F3N3O2S	473.51	33	12	0.33	4	6	2	126.96	102.01	3.47	4.92	6.14	4.14	5.23	4.78	-5.88	6.24E-04	1.32E-06	Moderately soluble	-6.8	7.52E-05	1.59E-07	Poorly soluble	-7.46	1.66E-05	3.51E-08	Poorly soluble	Low	No	Yes	Yes	No	Yes	No	Yes	-5.7	0	1	0	1	0	0.55	0	0	2	4.88
15.	O=C(C1NC2CC1C1C	C24	474	33	6	0.	4	6	3	133	123	3.	2.	2.	2.	3.	2.	-4.	3.	7.1	Mod	-4	1.1	2.5	Mod	-5.	2.0	4.3	Mod	Hi	No	Yes	No	Yes	No	Yes	Yes	-7.	0	1	0	0	0	0.	0	0	1	5.

12	2OC(O1)(C)C)Nc1ccc(c(c1)C1N=C(N)SC2(C1)CCCC2)F	H31FN4O3S	.59	67	.49	.27	19	37	59	51	11	75	15	9E-02	4E-05	erately soluble	.6	9E-02	2E-05	erately soluble	36	6E-03	4E-06	erately soluble	gh	51	55	89

Table S3. Molecular Docking interactions of reported compounds

amgen	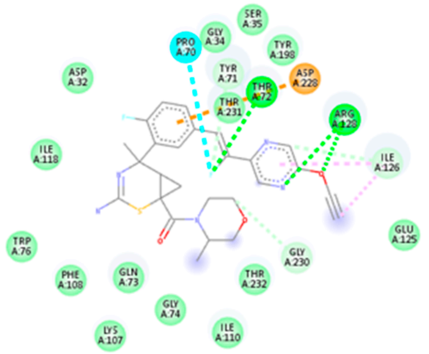	PF06751979	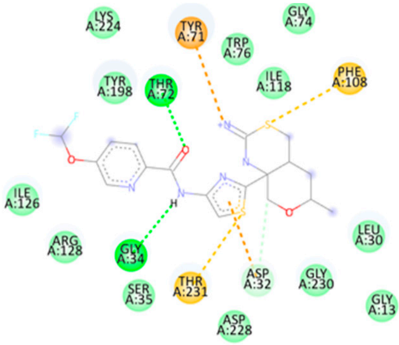
atabecestat	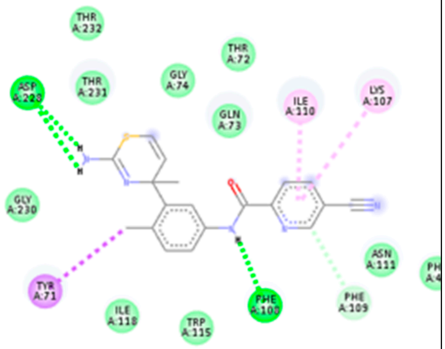	COMPOUND22	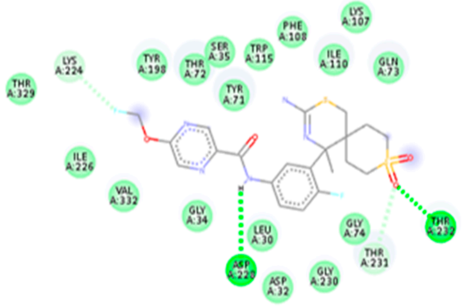
BI1181181	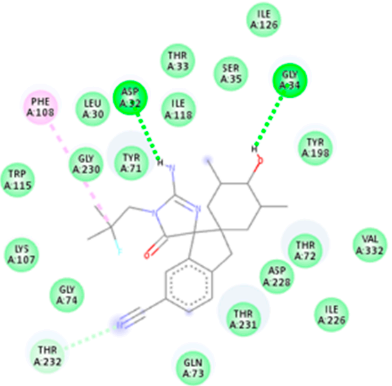	CTS21166	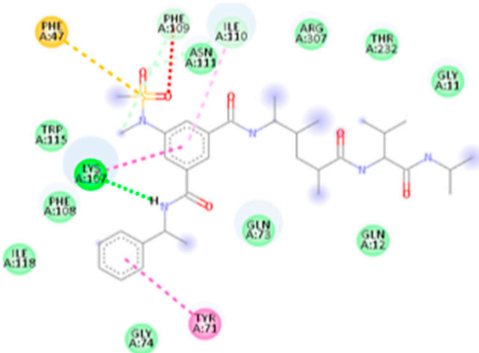
LY3202626	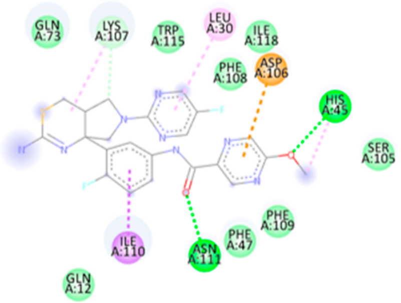	ELENBECESTAT	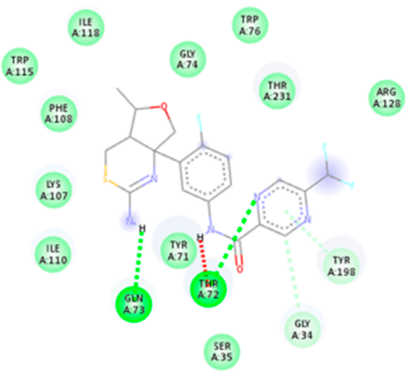

ELILILY	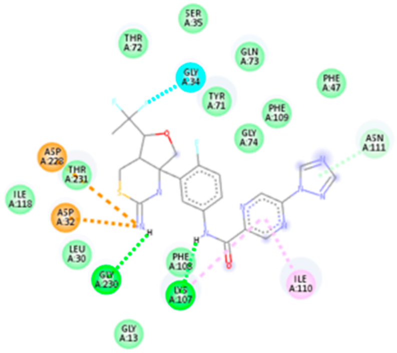	LANABECESTAT	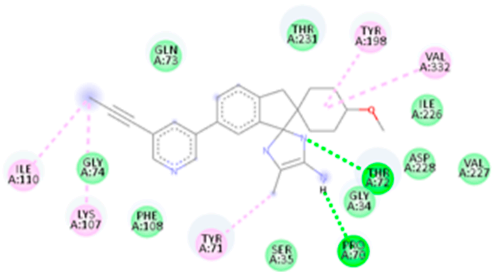
LY2811376	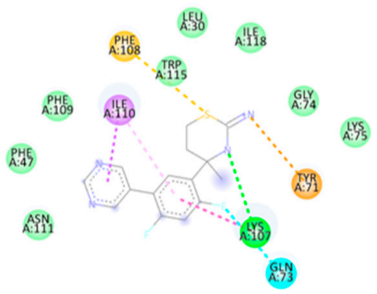	LY2886721	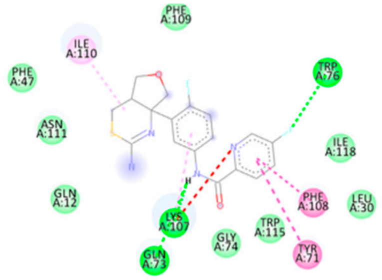
RG7129	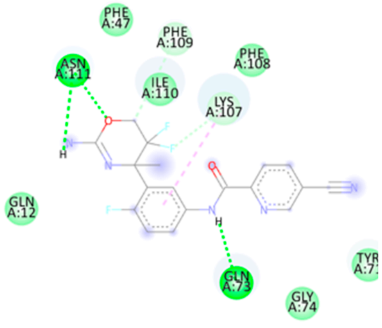	Umibecestat	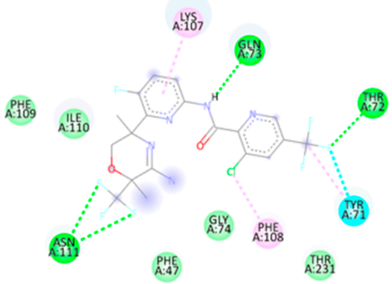
verubecestat	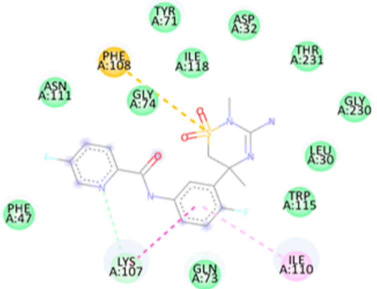	Wyeth bace-1 inhibitor	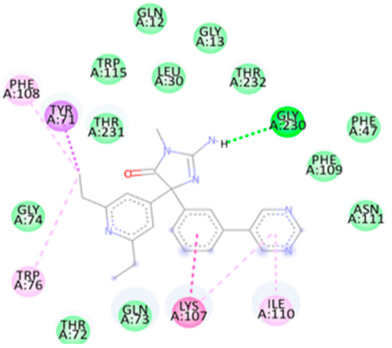

<table>
<tr>
<td>Compound 6 (10s loop)</td>
<td>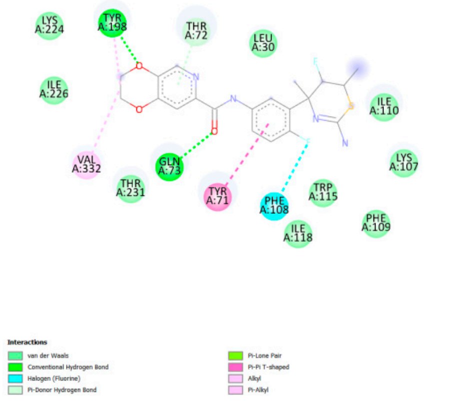
</td>
<td>JNJ_67569762</td>
<td>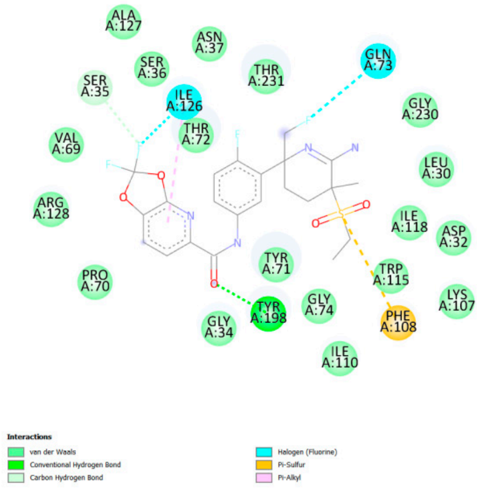
</td>
</tr>
<tr>
<td>Fujimoto</td>
<td>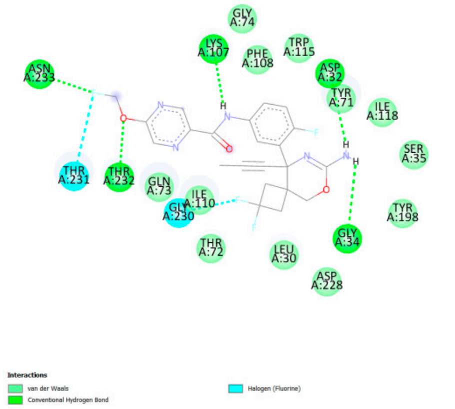
</td>
<td>Compound 8 (10s loop)</td>
<td>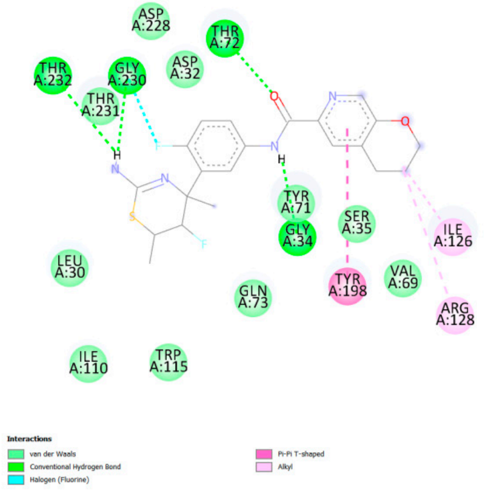
</td>
</tr>
</table>

Table S4. Molecular docking of 9.7 with BACE-1 and BACE-2. Molecule 9.7 was observed to bind appropriately within the key residues of the BACE-1 binding pocket. Important hydrogen-bond interactions were formed with Gln73, Asp228 and Thr231.The corresponding BACE-2 residues Gln89, Asp241 and Thr244 were not found to form similar interactions with the molecule, indicating its selectivity for BACE-1. Additionally, π-alkyl and π–π stacking interactions were contributed by Lys107 and Tyr71. Lys 107 was also found to engage in bonding with the fluorine atom of the indole moiety. However, the corresponding BACE-2 residues Asn123 and Tyr87were not observed to interact with molecule 9.7.

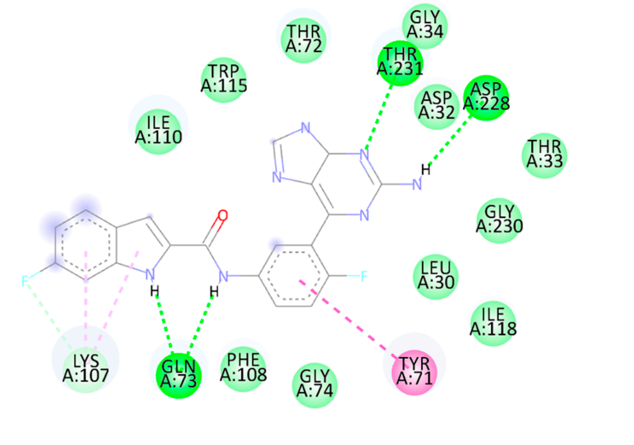

2-D interaction of 9.7 with BACE-1 (1FKN)

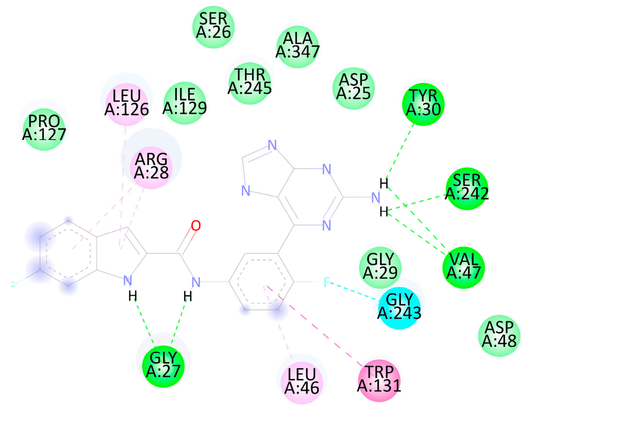

2-D interaction of 9.7 with BACE-2 (PDB: 7N4N)

Table S5. Binding Energy of reported BACE-1 compounds

**Compound**	**Binding energy (kcal/mol)**
LY2811376	-4.63
CTS21166	-5.97
Lanabecestat	-6.37
LY2886721	-5.26
LY3202626	-6.03
PF06751979	-5.11
RG7129	-5.6
Umibecestat	-4.35
Verubecestat	-5.87
Wyeth inhibitor	-6.42
Amgen inhibitor	-9.68
BI1181181	-10.61
Compound 6	-8.88
Compound 22	-9.2
Elenbecestat	-7.66
Elilily inhibitor	-8.94
Atabecestat	-9.09
Compound 6 (10s Loop)	-5.2
Compound 8 (10s loop)	-5.9

Fujimoto	-3.9
JNJ-67569762	-5.01

Table S6. Minimum and maximum fluctuating residues range in 1FKN-INH1 and 1FKN-VER

**Category**	**1FKN-INH1 (INH)**	**1FKN-VER (VER)**	**Observation**
Low flexibility (< 0.1 nm)	136 (35.45%)	140 (36.46%)	VER slightly increases rigidity in core/structured regions,
Medium flexibility (0.1–0.3 nm)	224 (58.42%)	219 (57.03%)	INH results in slightly more moderate dynamics
High flexibility (> 0.3 nm)	25 (6.52%)	25 (6.51%)	No change in highly dynamic regions

Table S7. Minimum and maximum fluctuating residues in 1FKN-INH1 and 1FKN-VER

	Low flexibility (< 0.1 nm) , green	Medium flexibility (0.1–0.3 nm), grey	High flexibility (> 0.3 nm), red
1FKN-INH1 (INH)	4-6,14,15-16,21,28-29,30,31-36,37,38-41,50,52,57,59-60,63,66,76,78,80-83,85,97-98,100-102,105,109,115-118,119-124,127,135,138,139-141,147-149,150-151,154,155,172-173,175-186,188-193,199,203-204,206,208,210,213,217,220,231,234,236-237,240-241,243-244,269-272,283-284,285-286,287,289,295-296,297-299,301-302,307-310,324,330-332,334-346,347-348,351-356,357-358,370-372<br>Total Residues136 (35.45%)	1-3,7-13,17-20,22-27,43-49,51,53-56,58,61-62,64-65,67-69,70-75,77,79,84,86-87,88,89-96,99,103-104,106-108,110-114,125-126,128-129,130-134,136-137,142-146,152-153,156-158,160-161,162-164,165-170,174,176-182,183,187-189,194-196,198-202,205,209,211-212,214-216,218-219,221-223,224-230,232-233,235-239,242-248,250-252,254-255,257-258,259-260,261-263,264-266,267-270,275-277,278-280,282,288-290,291-294,300-306,307-309,317-318,319-321,322-323,325-326,328-329,333,336-339,350,359-361,366-367,368-369,371,373-375,376-377,381-383<br>224 (58.42%)	159,163,255-256,271-274,310-316,361-365,378-380,384-385<br>25 (6.52%)
1FKN-VER (VER)	13,15-16,21,25,27-31,32-37,38-39,41-42,52,66-67,69,71,76,78,81-83,97-98,100-102,115-118,119-	2–12, 14, 17–20, 22–24, 26, 40, 43–51, 53–65, 68, 70, 72–75, 77, 79–80, 84–96, 99, 103–114, 125–126, 128–134, 136–	1,159-170,273,311-315,363-

	124,127,<br><br>135,138-141,147-149,151,172-173,175,177-178,183-184,186,188,190-193,195-197,198-199,200-202,203-204,<br><br>206,208,210,213,217,220-221,225-226,234,236-237,240-241,243-245,252,253,254,255,256,257,258,259,260,<br><br>261,262,263,264,265,266,267,268,269,270,271,272,273,274,275,276,277,278,279,280,281,282,283,284,285,<br><br>286-287,289,295-296,297,298-299,301-302,305,323-324,327,330-332,334,335,340-346,348,350-356,357-359,<br><br>370,382	137, 142–146, 150, 152–158, 171, 174, 176, 179–182, 185, 187, 189, 194, 205, 207, 209, 211–212, 214–216, 218–219, 222–224, 227–233, 235, 238–239, 242, 246–272, 274–282, 288, 290–294, 300, 303–304, 306–310, 316–321, 325–326, 328–329, 333, 336, 338–339, 349, 360–362, 368–369, 371–379, 381, 383–385	367,380
